# Unraveling single-molecule reactions via multiplexed in-situ DNA sequencing

**DOI:** 10.1038/s41467-026-77308-3

**Published:** 2026-08-29

**Authors:** Jagadish Prasad Hazra, Rebecca Andrews, Piers Turner, Qing Zhao, Horst Steuer, Afaf H. El-Sagheer, Abhishek Mazumder, Hafez El Sayyed, Mirjam Kümmerlin, Tom Brown, Achillefs N. Kapanidis

**Affiliations:** 1 Department of Physics, University of Oxford, Oxford, UK; 2 Kavli Institute for Nanoscience Discovery, Dorothy Crowfoot Hodgkin Building, University of Oxford, Oxford, UK; 3 Department of Chemistry, University of Oxford, Oxford, UK; 4 School of Chemistry & Chemical Engineering, Institute for Life Sciences, University of Southampton, Southampton, UK; 5 Trivedi School of Biosciences, Ashoka University, Sonepat, Haryana India; 6 Present Address: Max-Planck-Institute for Astronomy, Königstuhl 17, Heidelberg, Germany

**Keywords:** Single-molecule biophysics, Transcription

## Abstract

DNA sequence regulates complex reactions and protein-DNA interactions, yet sequence effects remain poorly understood due to the lack of direct, high-throughput approaches to study sequence-dependence at the single-molecule level. Here, we introduce Single-molecule Phenotyping and In-Situ Sequencing (SPIN-Seq), a DNA-based, protein-free method that links functional and structural properties of a single DNA molecule with the sequence of that same molecule. After performing functional assays on immobilized DNA molecules, SPIN-Seq uses sequencing-by-transient-hybridization on the same surface and instrument to read sequences in each immobilized DNA molecule, directly linking phenotype and genotype. Applying SPIN-Seq, we dissected the interaction of a transcription factor with its target sequence, and revealed the sequence-dependence of RNA polymerase pausing and reaction-path branching during initial transcription. Our method provides powerful, systematic ways to understand complex molecular mechanisms and their sequence dependence.

## Abstract (web summary)

The authors develop SPIN-Seq, a method that directly links the sequence of individual DNA molecules to their functional behavior, revealing how DNA sequence impacts protein-DNA interactions and the kinetics of biological reactions on nucleic acids.

## Introduction

Interactions of nucleic acids with proteins and other biomolecules are key to genetic processes and biotechnology. Analysis of such interactions relies heavily on structural, biochemical, and biophysical approaches; single-molecule methods, in particular, offer key insight into nucleic-acid interactions, often capturing them in real-time^1–6^. However, single-molecule experiments typically study one sequence at a time and do not offer a global view of how DNA sequence affects protein–DNA interactions.

An attractive route to explore sequence-dependence at the single-molecule level couples single-molecule DNA sequencing (nanopore- or fluorescence-based) to analysis of protein-driven processes on DNA strands being sequenced^7–9^; however, these approaches are confined to proteins translocating on nucleic acids, and some require special surfaces.

Recently, two approaches enabled multiplexing on DNA–DNA and protein–DNA interactions^10,11^; both involved single-molecule fluorescence imaging on custom-built microscopes, DNA amplification, Illumina sequencing, and linking of single-molecule and sequence readouts. While these powerful approaches offered insight into how sequence controls the structure and dynamics of folded DNA and protein–DNA complexes, they had not addressed labeled proteins or non-equilibrium reactions and their kinetics, in part due to their dependence on a solid support (MiSeq chips) suboptimal for single-molecule imaging of labeled proteins, and in part due to the confinement of such methods to a single field-of-view while monitoring a non-equilibrium reaction in real-time.

**Fig. 1:**
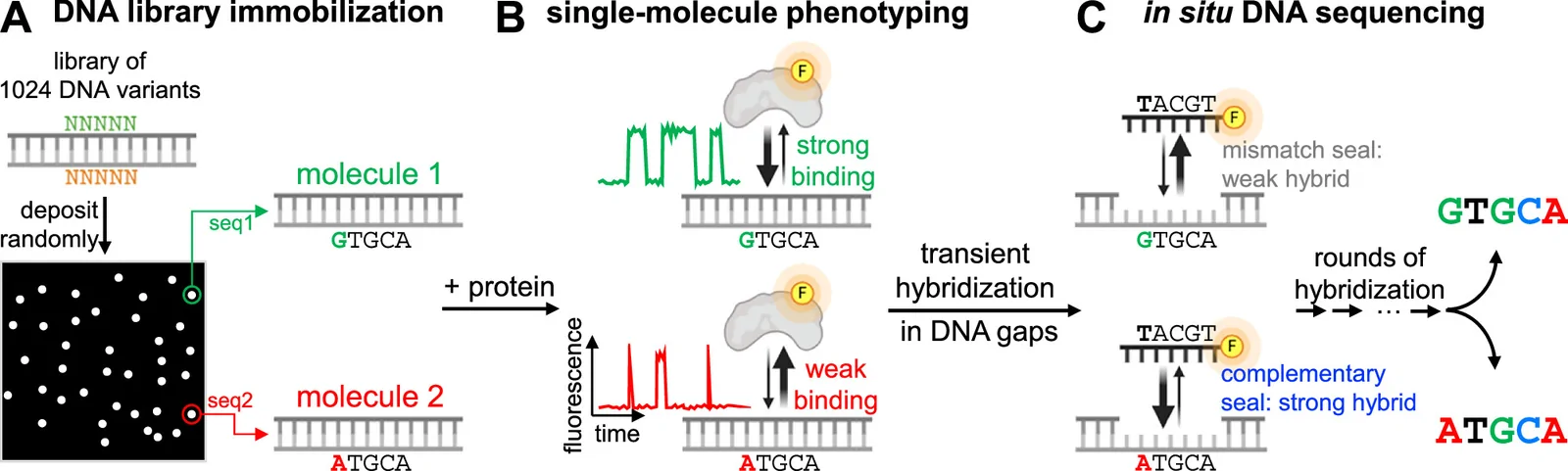
A single-molecule method that links protein–DNA interactions to DNA sequence. **A** A library of DNA molecules (e.g., with 4^5^ DNA sequences, reflecting a 5-nt randomized sequence NNNNN) is immobilized randomly on a surface, and imaged using widefield single-molecule fluorescence microscopy. Different molecules (white filled circles) contain different DNA sequences. **B** Proteins are added, and the single-molecule kinetics of protein–DNA interactions (its “single-molecule phenotype”) are recorded using fluorescence signals (here, due to binding of labeled proteins). **C** Surface-immobilized DNA molecules are converted into gapped DNA molecules in situ. Short oligonucleotides (DNA “seals”) are added and bind transiently to the gap; the extent of binding differs between fully complementary seals and mismatched seals, forming the basis for recovering the sequence of each molecule and linking it to single-molecule phenotypes. Some elements in the figure were created in BioRender. Kapanidis, A. (2026) https://BioRender.com/oxjtian.

Here, we introduce single-molecule phenotyping and in situ sequencing (SPIN-Seq; Fig. 1), a method that links the functional properties of a single DNA molecule (its “single-molecule phenotype”) with the sequence of the *same* molecule, on the same surface and instrument. This phenotype can involve both structure and dynamics, e.g., distances within protein–DNA complexes, or kinetics of reactions and interactions. SPIN-Seq starts by immobilizing DNA libraries on standard glass coverslips; DNAs then interact with proteins, and dynamics are recorded. After protein removal, a new sequencing-by-hybridization approach reads sequences in each DNA molecule, and links them to its single-molecule phenotype to gain mechanistic insight. Our assay can read up to 5 nucleotides in length, covering libraries of up to 1024 DNA sequences and opening a new avenue for exploring protein–DNA interactions.

## Results

### Single-molecule hybridization on DNA gaps

To perform in situ single-molecule sequencing, we leveraged the transient binding of fluorescent single-stranded (ss) DNA oligonucleotides (“seals”) to surface-immobilized DNAs featuring short ssDNA gaps^12^ (Figs. 1, right and 2A, B). Such transient binding is used in high-throughput endeavors, such as DNA-PAINT^13^. We selected gapped-DNA templates instead of ssDNA, since the coaxial stacking at the gap ends stabilizes the bound seal; the gap also defines the segment to be sequenced. Base-calling relies on distinguishing which DNA seals are fully complementary to the gap (as opposed to seals that carry one or more mismatches). The short gap length allows bound seals to thermally dissociate and be replaced by seals from the surrounding solution; repeated seal binding increases statistics and interrogation accuracy, while eliminating photobleaching.

**Fig. 2:**
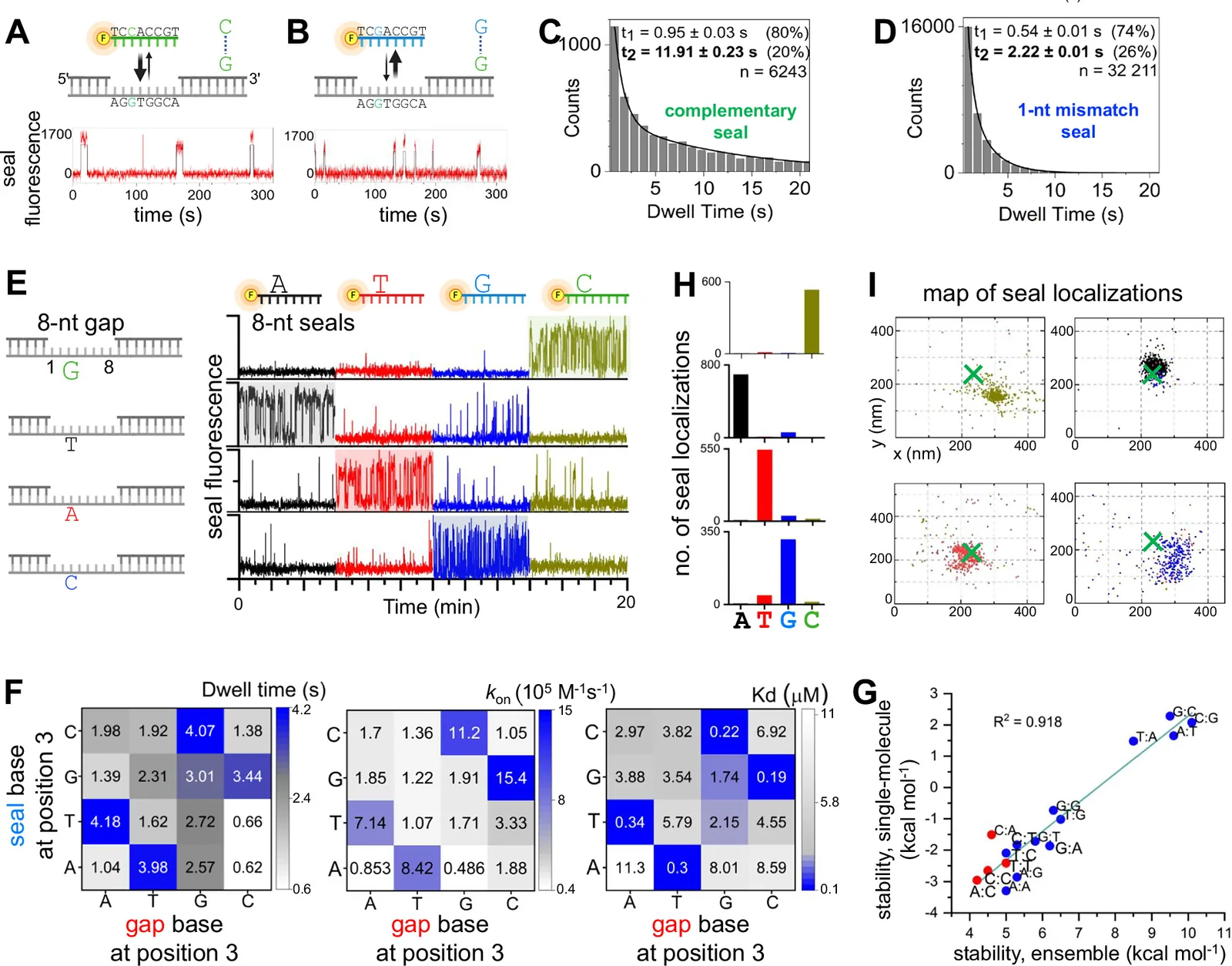
Reading single DNA bases via transient DNA binding of short DNA oligos to gapped DNA. **A** Top: Schematic of a fully complementary 8-nucleotide (nt) seal binding in 8-nt gapped-DNA. Bottom: fluorescence intensity time-traces (red) for ATTO647N-labeled DNA seal binding to gapped DNA. Traces are fitted (black) using Hidden Markov Modeling (HMM), identifying bound states (high-intensity dwells) and their dwell times. **B** As in panel (**A**), but for a G:G mismatch at gap position 3 due to the use of a different seal; the mismatch drastically destabilizes gap-seal binding. **C**, **D** Dwell-time distributions for bound states for the two seals. Data fit best to double-exponential distributions, likely as a result of base stacking of seals in the gap. n, number of binding events. **E** First row: an 8-nt DNA gap within an immobilized molecule is sequentially interrogated at gap position 3 (where the base is a G) using four seals that differ only on the base opposed to the interrogated position. Only the seal featuring a C at position 3 (green DNA) shows strong gap binding; the remaining seals show negligible binding. Rows 2–4: as in row 1, but for gaps featuring a T, A, or C at gap position 3. Only the fully complementary seal shows substantial binding. **F** Kinetic and thermodynamic analysis of interactions of complementary and 1-nt mismatched seals with gapped-DNA. Left, dwell times of different 8-nt seals (each featuring a different standard base corresponding to gap position 3 on 8-nt gaps (each featuring a different standard base at gap position 3). Fully complementary pairs exhibit longer dwell times compared to 1-nt mismatched pairs. Number of molecules is *Ν* = 395 for Gap8-G^3^; *N* = 376 for Gap8-C^3^; *N* = 515 for Gap8-A^3^; and *N* = 412 for Gap8-T^3^. Middle, binding on-rates for the same pairs as in the left panel. Fully complementary pairs exhibit faster on-rates compared to 1-nt mismatched pairs. Right, equilibrium dissociation constants (*K*_d_ values) for the same pairs as in the left panel. Fully complementary pairs exhibit lower *K*_d_ (higher affinity) compared to 1-nt mismatched pairs. **G** Comparison of thermodynamic stabilities obtained from single-molecule analysis with the stability obtained from spectrophotometric thermodynamic analysis (15), which examined mismatches of base X in an A-X-T context; our sequence context is C-X-A. The results from both analyses are highly correlated (*R*^2^ = 0.918). Red points denote higher uncertainty in the ensemble analysis. **H** Number of single-molecule seal localizations per seal interrogation. **I** Map of seal localizations clustering around the interrogated gap DNA molecule (marked by a green cross). Some elements in the panels (**A**, **B**) were created in BioRender. Kapanidis, A. (2026) https://BioRender.com/adu87tb. Source data are provided as a Source Data file.

To read sequence information in gapped DNA via transient seal binding, we examined the binding between a surface-immobilized 8-nt gapped DNA and labeled 8-nt seals fully complementary to the gap (Fig. 2A and Supplementary Table 1 for all DNA sequences). When a seal binds to the DNA gap, the fluorescence intensity in the vicinity of the gapped DNA molecule increases to a high value lasting until a seal dissociates (Fig. 2A, bottom). Analysis of the dwell-time distribution for the high-intensity state (Fig. 2C) revealed two characteristic lifetimes for seal binding, the longer of which signifies stable binding (t_2_ ~12 s). Experiments using an 8-nt seal with a 1-nt mismatch (G:G) to gap position 3 (Fig. 2B) showed a large decrease for both lifetimes, and especially for the t_2_ lifetime, which becomes >5-fold shorter (t_2_ ~2.2 s; Fig. 2D). These results confirmed that a single-nt mismatch drastically decreases seal binding, as was seen for other short hybrids^14^, offering a basis for short-read single-molecule sequencing.

### Sequencing single bases via transient DNA hybridization

We then explored reading one base on a single immobilized gapped-DNA molecule (as opposed to detecting differences between ensembles of single molecules in the previous section). Using a modified hybridization buffer (to increase sampling of seal binding; Methods), we interrogated a G base at position 3 in an 8-nt gap molecule (Gap8-G^3^, where the superscript denotes the interrogated gap position; Fig. 2E, left) by sequentially incubating it with four seals; each seal differs only in the base opposed to the interrogated gap base. Each seal with a 1-nt mismatch (seals S8-A^3^, S8-T^3^, S8-G^3^) showed negligible or very weak gap binding (Fig. 2E, top row and Supplementary Fig. 1). In contrast, we observed extensive binding of the seal fully complementary to the 8-nt gap (seal S8-C^3^, forming a C:G pair at gap position 3; Fig. 2E, top row, green trace). We obtained similar results for all bases at gap position 3 (Fig. 2E and Supplementary Fig. 1), supporting the generality of the approach.

To characterize gap-seal interactions, we measured seal dwell times and binding frequency for all gap-seal combinations (Fig. 2F). Complementary seals bound 1.3- to 5.5-fold longer and 4- to 23-fold faster than seals with 1-nt mismatches. Overall, affinity differences between complementary and 1-nt mismatch seals ranged from 8- to 45-fold. Different mismatches disrupted binding to a different degree. Mismatches containing guanines are the least disruptive, with G:G and T:G reducing affinity only by 8- and 10-fold, respectively. In contrast, mismatches containing cytosines are the most disruptive, with C:A and C:C reducing affinity by 36- and 45-fold, respectively (Fig. 2F). Our results agree with ensemble measurements^15^ (Fig. 2G), and computational predictions of thermodynamic stability^16,17^ (Supplementary Fig. 2), and offer insight into canonical and non-canonical base-pairings and nucleic-acid interactions in the context of DNA gaps.

To “call” an interrogated base, we first quantified the extent of gap-seal binding by counting single-molecule localizations of different seals in close proximity to a single gap molecule (i.e., by counting the number of times the fluorescence of a bound seal is detected within a small area centered at the location of a gap molecule). For Gap8-G^3^, we detected a large number of localizations for the complementary seal (~550 in Fig. 2H, and 250–750 in the examples of Supplementary Fig. 3A), whereas the three mismatched seals gave a very small number of localizations, typically less than 10% of those seen for the complementary seal. The complementary seal localizations also clustered well in the nanoscale, whereas any localizations of the mismatched seals were more scattered (Fig. 2I). This pattern was similar for the remaining three bases in gap position 3 (Fig. 2H, I and Supplementary Fig. 3B–D).

**Fig. 3:**
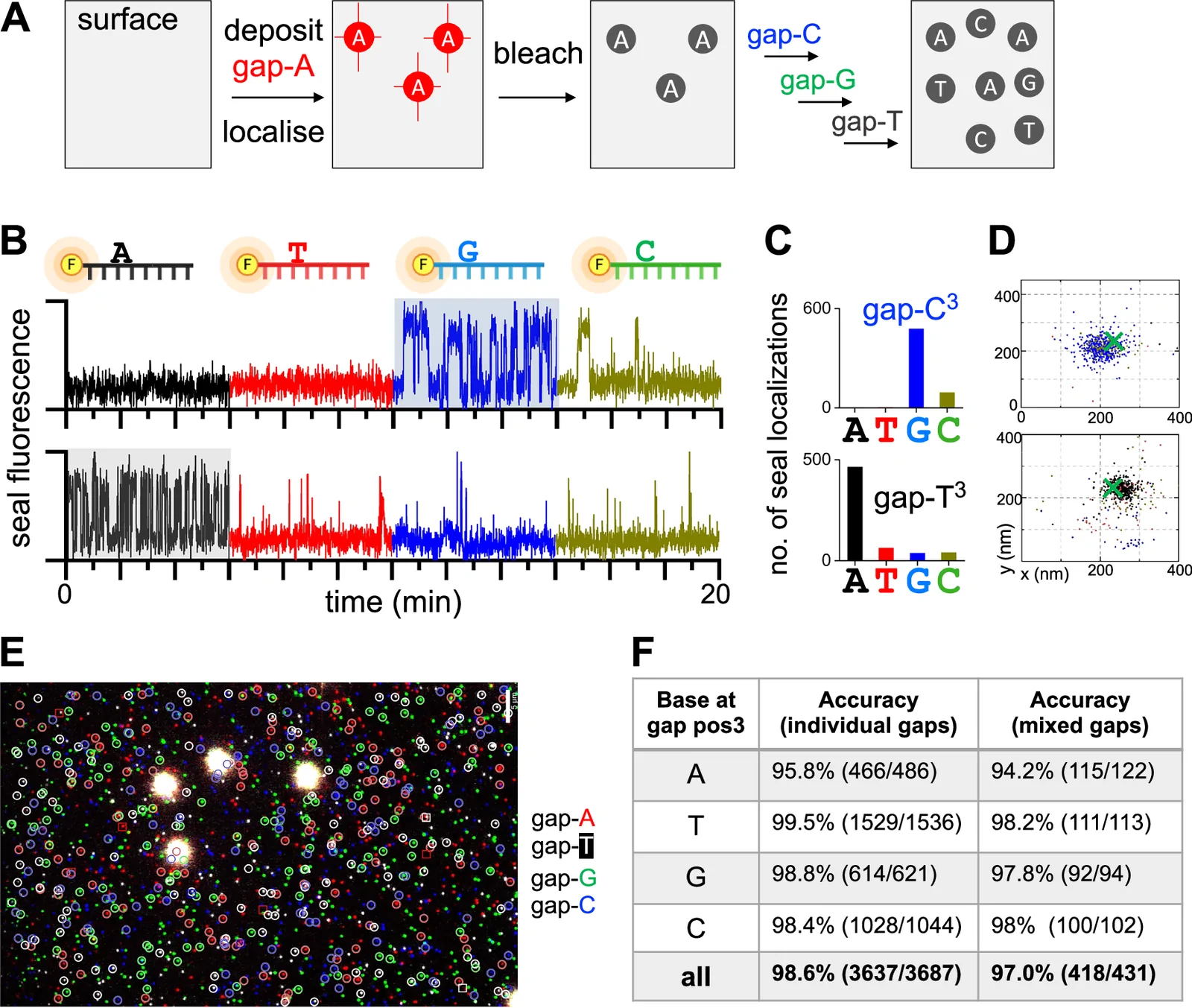
Validation of sequencing by transient binding to DNA gaps. **A** Analysis of surfaces with mixed sequences. Sequential deposition (using cycles of deposition, localization, and bleaching) of 4 different DNA gap molecules of known sequence. **B**, **C** Sequential interrogation of the deposited DNA by seals specific for gap position 3. The extent of binding (based on seal localization counts) in the top and bottom traces identifies a Gap-C^3^ and a Gap-A^3^ molecule, respectively. **D** Map of seal localizations clustering around the two interrogated gap DNA molecules in panel B. Green cross: location of a gap molecule. **E** Field-of-view (50 × 80 μm) of molecules sequenced for gap position 3 in the experiment in panel (**A**). Circled molecules were identified correctly; in-square molecules were assigned incorrectly. White spots: fiducial markers. Scale bar, 5 μm. **F** Base-calling accuracies for interrogation of single sequences (as in panel 2**E**) and of a mixture of four sequences (as in panels 3**A**, **B**). Some elements in panel 3**B** were created in BioRender. Kapanidis, A. (2026) https://BioRender.com/adu87tb. Source data are provided as a Source Data file.

We then proceeded by selecting gap molecules that are sufficiently sampled by transient binding (using a “base-calling threshold” that relied on the total number of seal localizations per gap molecule) and choosing as the complementary seal the one with the most localizations (Supplementary Fig. 4; Methods). This analysis was ~98.8% accurate (614 out of 621) in detecting a G at position 3 and similarly accurate for the remaining 3 bases (~98.6%; Fig. 3F).

To demonstrate our ability to address a mixed population of sequences, we sequentially deposited each single-nucleotide variant for gap position 3 on the same surface, generating the “ground truth” for the sequence of each molecule (Fig. 3A). We interrogated the surface as before (Fig. 3B–D), mapped read bases to the pre-registered base identities (Fig. 3E), and demonstrated a ~97% base-calling accuracy (Fig. 3F), establishing that we can read any standard base at a single position with high accuracy.

We also examined how well our sequencing approach works on A/T-rich sequences, which may be challenging due to weaker base-pairing potential. We constructed an 8-nt-gapped DNA where all gap bases were adenines (AAAAAAAA, or “8Α”), and interrogated it using the same sequential approach we used for mixed sequences. We observed much higher binding for the fully complementary seal (i.e., having T at seal position 3), and little binding for the three 1-nt mismatched seals (Supplementary Fig. 5), identifying the A base at position 3 with ~97% accuracy, and demonstrating our ability to read A/T-rich sequences.

### Sequencing single bases using competitive inhibition of gap hybridization

**Fig. 4:**
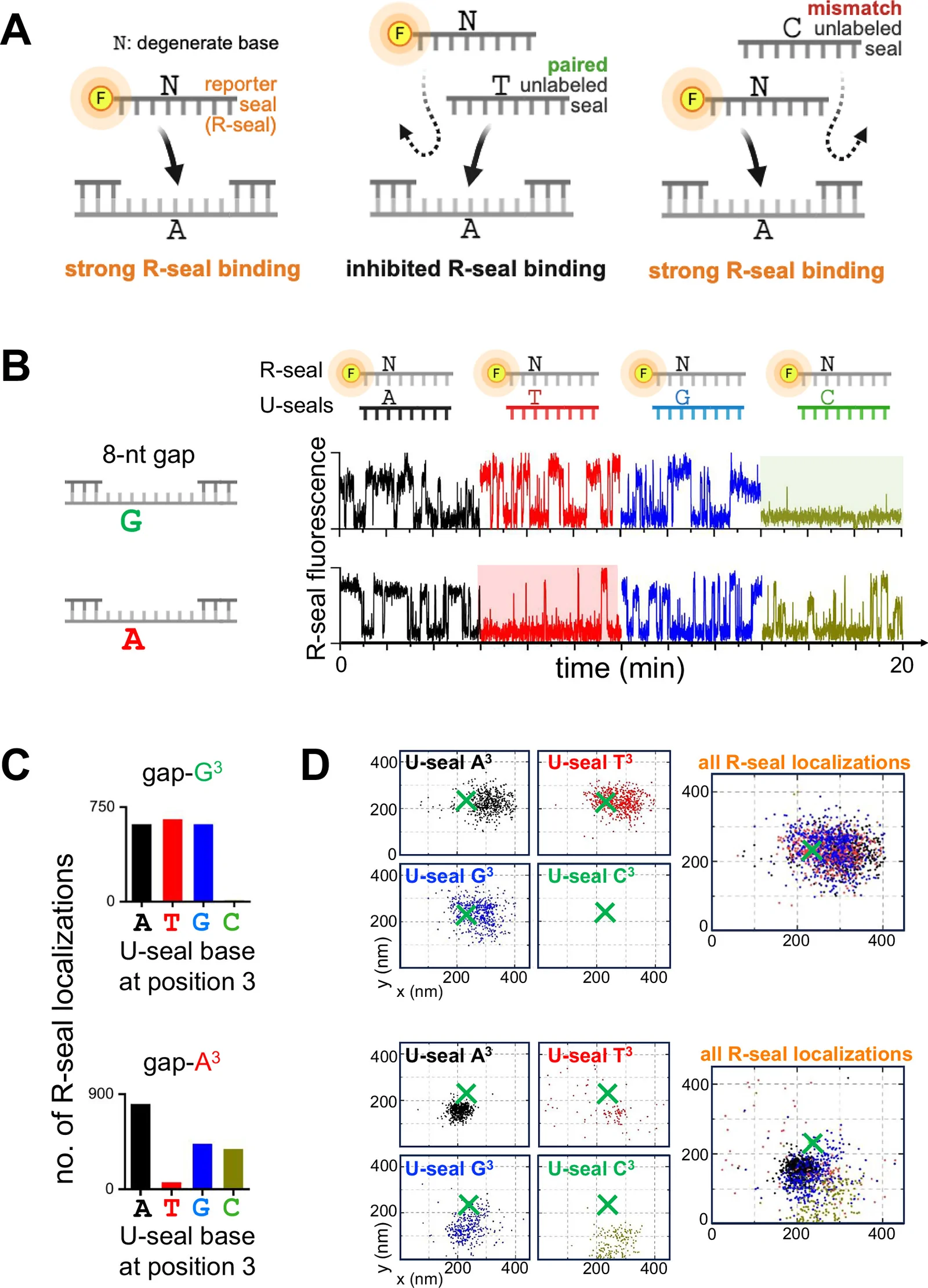
Single-molecule sequencing via competitive inhibition of transient hybridization. **A** Concept. Left: a labeled seal (R-seal; containing a degenerate base N) binds transiently to a gap and gives a fluorescence signal. Middle: in the presence of a complementary (“paired”) unlabeled seal, R-seal binding is reduced, and fluorescence is suppressed. Right: in the presence of a mismatched unlabeled seal, R-seal binding is maintained, and fluorescence is observed. **B** Reading single nucleotides. Top row: examining gap position 3 in Gap8-G^3^ using 200 nM of labeled R-seal and a set of four unlabeled U-seals (added sequentially, all at 600 nM); the complementary U-seal (U8-C^3^, in green) suppresses R-seal binding, enabling base identification. Bottom row, same as above, but for Gap8-A^3^. **C** Counts of R-seal localizations. The count for the complementary U-seals is very low compared to the three 1-nt mismatch seals. **D** Left: mapped R-seal localizations from single U-seals; green crosses denote the location of the interrogated gap molecule. Right: superimposed localizations from all four seals. Some elements in panels (**A**, **B**) were created in BioRender. Kapanidis, A. (2026) https://BioRender.com/26ou6mh. Source data are provided as a Source Data file.

To sequence multiple bases, we had to account for the unknown sequence context around each interrogated position and simplify the set of seals to make the assay more scalable. We thus introduced a competitive-inhibition version of our assay that uses DNAs containing degenerate bases (“N”) that feature 25% of each standard base at a single position. Each interrogation uses a mixture of two seal DNAs competing for gap binding: a reporter seal (“R-seal”; either singly labeled and fluorescent prior to binding, or fluorogenic and quenched prior to binding) that contains degenerate bases at all positions examined and binds the gap efficiently in order to generate a strong signal (Fig. 4A, left). The second seal is unlabeled (“U-seal”); when the U-seal is fully complementary to the gap, it outcompetes the R-seal, suppressing its gap binding and the associated fluorescence (Fig. 4A, middle). However, if the U-seal contains even a single-nucleotide mismatch to the gap sequence, it is outcompeted by R-seal, leading to high fluorescence (Fig. 4A, right). For every position examined, we perform four sequential interrogations, each involving a mix of the R-seal with one of four U-seals (each U-seal containing one of the four standard bases at the position opposite to the interrogated site); only the complementary U-seal reduces R-seal binding, thus reading the base. This version of the assay has the advantage of requiring only one labeled DNA strand (the R-seal) for the entire interrogation, making it more practical and inexpensive.

To test our approach, we examined an 8-nt gap (Gap8-G^3^) using an 8-nt R-seal with a degenerate base opposed to gap position 3 (R8-N^3^), and four 8-nt U-seals, each featuring one of the standard bases opposed to gap position 3 (Fig. 4B, top). Using optimized R-seal and U-seal concentrations (Supplementary Figs. 6 and 7), we sequentially added different R-seal/U-seal mixtures to the gap and tracked R-seal binding; we observed very low R-seal binding for the mixture containing the complementary U-seal (U8-C^3^), and substantial binding for the 1-nt mismatched seals (Fig. 4B, top and Supplementary Fig. 8C). Using localization counting for base-calling (Methods), we detected 500 out of 502 molecules exhibiting lower R-seal binding for U8-C^3^ compared to the other U-seals, yielding a ~99.6% base-calling accuracy. Overall, we obtained high accuracy for all bases at gap position 3 (~97.1%; Fig. 4B–D, bottom, Supplementary Fig. 8, and Supplementary Table 2), as well as for mixed sequences (~98.5%; Supplementary Fig. 9 and Supplementary Table 2), establishing that competitive inhibition can be used for reading single positions with high accuracy.

### Sequencing of up to five bases

**Fig. 5:**
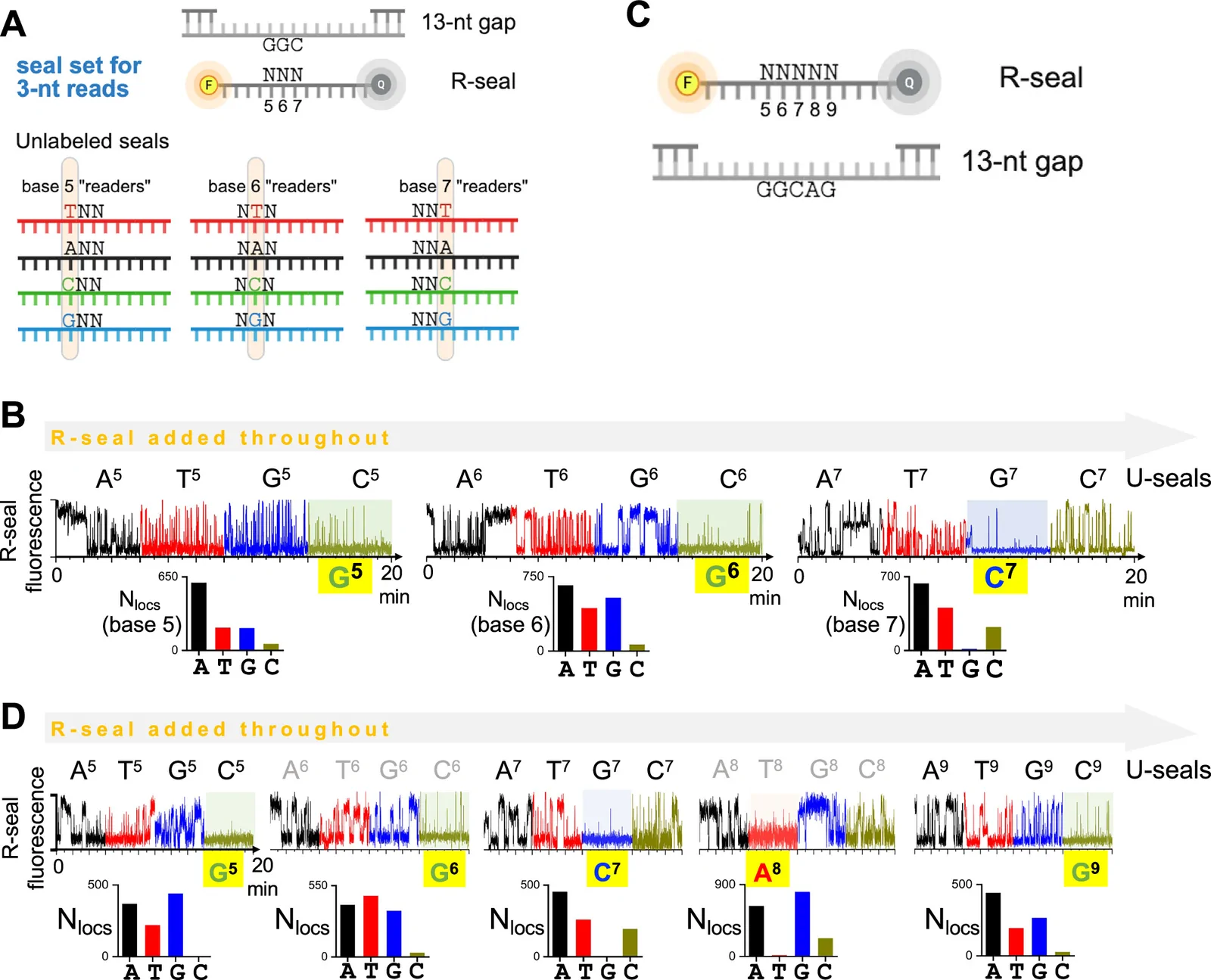
Single-molecule sequencing via competitive inhibition: multi-base sequencing. **A** DNA design for reading a 3-base sequence (here, G^5^G^6^C^7^) using competitive inhibition. For details, see the main text. **B** Demonstration of ability to read three bases using R-seal/U-seal combinations. R-seal (R13-N^5^N^6^N^7^) concentration was kept at 500 nM throughout all interrogations. Each of the three bases was evaluated on a different surface. A single base is read via a series of four sequential interrogations with four U-seals (1.5 μM); here, we refer to each seal using only the identity and position of the interrogating U-seal base (e.g., “Α^5^” instead of “U13-A^5^N^6^N^7^”). Only complementary U-seals substantially decrease R-seal binding (highlighted traces). Bottom, number of localizations for each seal interrogation. **C** DNA design for sequencing a 5-base sequence (here, G^5^G^6^C^7^A^8^G^9^) using competitive inhibition. **D** Demonstration of ability to read five bases. Experiments as for 3-base sequencing, apart from that, the R-seal (R13-N^5^N^6^N^7^N^8^N^9^) and U-seals were both added at 1 and 2 μM, respectively. Results shown as in panel (**B**). Some elements in panels (**A**, **B**) were created in BioRender. Kapanidis, A. (2026) https://BioRender.com/6z5vuh4. Source data are provided as a Source Data file.

Sequencing three bases using competitive inhibition required increasing both the number of degenerate bases in the R-seal (from 1 to 3) and the gap length (to 13 nt; Fig. 5A). Adding degenerate bases decreases the concentration of the fully complementary seal (e.g., for seals with three degenerate bases, only one in 64 sequences is fully complementary), effectively lowering the hybridization on-rate. To maintain frequent but short R-seal binding to the gap, and maximize the assay contrast, we adjusted our R-seal and U-seal concentrations, added a quencher to the R-seal to suppress fluorescence of unbound seals, and modified our hybridization buffer (Supplementary Figs. 10–12 and Methods). We also modified our U-seals to include degenerate bases; e.g., for reading position 5 within a gap with unknown sequence at 5, 6, and 7 (Gap8-N^5^N^6^N^7^), we used 4 U-seals: U13-A^5^N^6^N^7^, U13-C^5^N^6^N^7^, U13-G^5^N^6^N^7^, and U13-T^5^N^6^N^7^ (Fig. 5A).

To read a base at position 5, we performed four sequential interrogations using mixtures of the R-seal and each of the four U-seals. Our results showed that R-seal binding decreases only with U13-C^5^N^6^N^7^ (abbreviated as “C^5^”; Fig. 5B, green trace), and that we can read a G at gap position 5 with ~96.4% accuracy (240 out of 249 molecules). We obtained similarly high accuracies for gap positions 6 and 7 (~96.5%; Fig. 5B, Supplementary Fig. 13A–C, and Supplementary Table 2), successfully identifying all three bases. To establish our ability to read multiple bases sequentially, we also sequentially interrogated a three-base sequence (GGC) on the same set of immobilized DNA gap molecules (Supplementary Fig. 13D), achieving ~99% base-calling accuracy (188 out of 189 molecules).

We also examined a 5-base sequence by using the same 13-nt gap, increasing the number of degenerate bases in the R-seal and U-seals (to 5 and 4, respectively; Fig. 5C), and adjusting the R-seal and U-seal concentrations (Supplementary Figs. 14 and 15). For gap position 5, only seal U13-C^5^N^6^N^7^N^8^N^9^ (abbreviated as “C^5^”) substantially decreased R-seal binding (Fig. 5D and Supplementary Fig. 16), detecting the G base with ~95% accuracy (195 out of 205 molecules). We achieved similar accuracies (~97%) for the remaining 4 positions (Fig. 5D, Supplementary Fig. 16, and Supplementary Table 2).

Our results show that we can sequence up to 5 bases with high accuracy, paving the way for sequencing 4^5^ = 1024 sequences on the same surface (requiring 4 × 5 = 20 interrogation rounds), and linking them to single-molecule function.

### Linking protein–DNA interactions to DNA sequence for single molecules

**Fig. 6:**
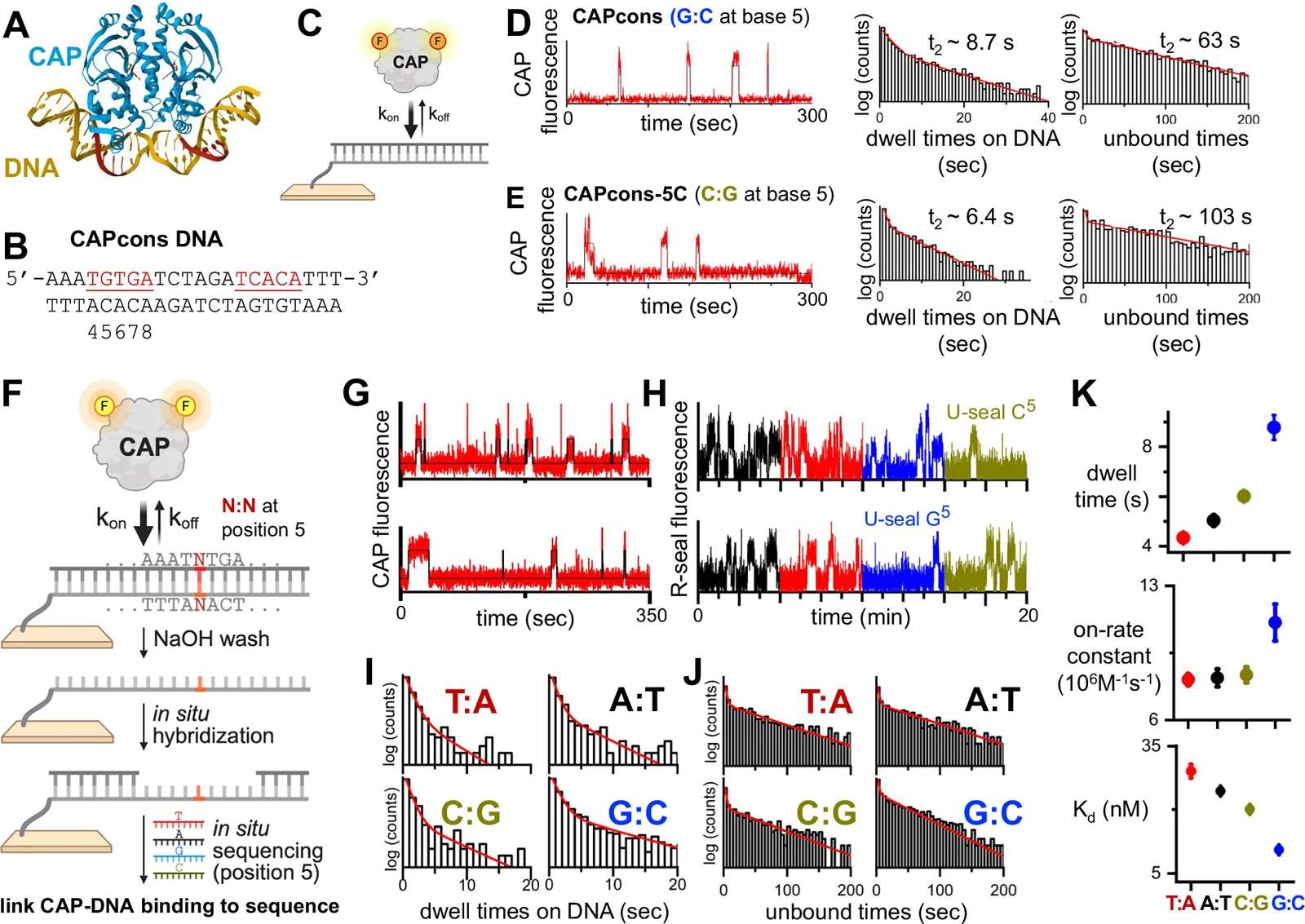
Linking kinetics of protein–DNA interactions to DNA sequence: transcription factor binding to its cognate DNA. **A** Structure of *Escherichia coli* catabolite activator protein (CAP) bound to its consensus DNA site (1CGP); red segments denote the 5-bp sequences conferring sequence specificity. **B** CAPcons sequence. **C** Labeled CAP binds to DNA variants transiently, yielding on- and off-rates. **D** Left: CAP binding to CAPcons DNA. HMM analysis identifies a high-intensity state (CAP–DNA complex), and a low-intensity state (free DNA). Right: dwell-time and off-time distributions fit best to double-exponential distributions; the longer lifetimes (t_2_) identify the lifetimes for stable binding and the frequency of stable complex formation. **E** As for (**D**), but for the CAPcons variant with C:G at position 5. **F** Linking kinetic phenotype to sequence. After evaluating CAP binding to surface-immobilized CAPcons variants, the DNA is denatured, and gapped-DNA is formed via in situ hybridization of two flanking strands; gap position 5 is then read. **G** CAP binding to the variant CAPcons library. **H** Gap interrogation of the two DNA molecules in panel (**G**) using competitive inhibition; the variants are identified as 5G (top) and 5C (bottom). **I** Dwell-time histograms for bound CAP for CAPcons variants. **J** Dwell-time histograms for unbound CAP for CAPcons variants. **K** Dwell times, association rate constants *k*_on_, and *K*_d_ values for CAPcons variants; error bars: SEM from three experiments. Some elements in panels (**C**, **F**) were created in BioRender. Kapanidis, A. (2026) https://BioRender.com/n0v65jc. Source data are provided as a Source Data file.

To link single-molecule phenotypes to sequence, we examined DNA interactions of transcription factor catabolite activator protein (CAP^18^; Fig. 6A). In the presence of cyclic adenosine monophosphate (cAMP), CAP binds with high affinity to consensus sequence 5′-AAATGTGATCTAGATCACATTT-3′ (CAPcons^19^), with the underlined sequences controlling specificity (Fig. 6B).

To observe CAP–DNA binding, we surface-immobilized CAPcons DNA, added labeled CAP in the presence of cAMP, and observed transient CAP binding to DNA (Fig. 6C, D, left). CAP dwell times on DNA (Fig. 6E, middle and Supplementary Table 3) showed a lifetime *t*_2_ ~8.7 s, which reflects stable CAP binding to CAPcons DNA; this assignment was supported by control experiments in the absence of DNA or in the absence of cAMP (Supplementary Fig. 17). Binding-frequency analysis (using unbound-time distributions; Fig. 6D, right) yielded an association rate constant *k*_on_ ~1.3 × 10^7^ M^−1^ s^−1^ and a *K*_d_ ~9.1 nM. A variant featuring a C:G pair at position 5 (as opposed to the consensus G:C) showed weakened CAP–DNA interactions (*t*_2_ ~6.4 s; *k*_on_ ~7.7 × 10^6^ M^−1^ s^−1^; Fig. 6E and Supplementary Table 3), and twofold reduced affinity (*K*_d_ ~20 nM).

We then linked CAP–DNA binding for a single DNA molecule to the sequence of the same DNA molecule (Fig. 6F) for a library of surface-immobilized CAPcons DNA molecules, where position 5 of CAPcons was randomized. In step 1, we evaluated CAP–DNA binding as above. In step 2, the dsDNA was denatured, leaving only the biotinylated bottom DNA strand on the surface; subsequently, two DNA strands were hybridized efficiently to the surface-immobilized strand in situ (Supplementary Fig. 18) to form a surface-immobilized gapped DNA with a 9-nt gap matching the first nine bases of CAPcons (5′-AAATGTGAT-3′).

In step 3, we analyzed the same field-of-view and sequenced position 5 using competitive inhibition. Using the surface locations of each protein–DNA binding and sequencing event, we linked CAP–DNA binding events (from step 1; Fig. 6G) to sequencing traces for single molecules (Fig. 6H and Supplementary Fig. 19). Binding analysis showed that G:C (known to form two hydrogen bonds from guanosine N7 and O^6^ atoms to CAP Arg180 side chain^18,20^) leads to a slower off-rate and a faster on-rate relative to the other variants (Fig. 6I–K and Supplementary Table 3), leading to a *K*_d_ 1.9- to 2.7-fold lower than the non-consensus variants, corresponding to a ~0.65–1.0 kT (~0.4–0.6 Kcal/mole) stabilization of the complex versus non-consensus variants. Our results reproduced those from pure sequences (Fig. 6D, E) and established our ability to work with labeled proteins and to link protein–DNA interactions with DNA sequence.

### Sequence-dependence of initial transcription

**Fig. 7:**
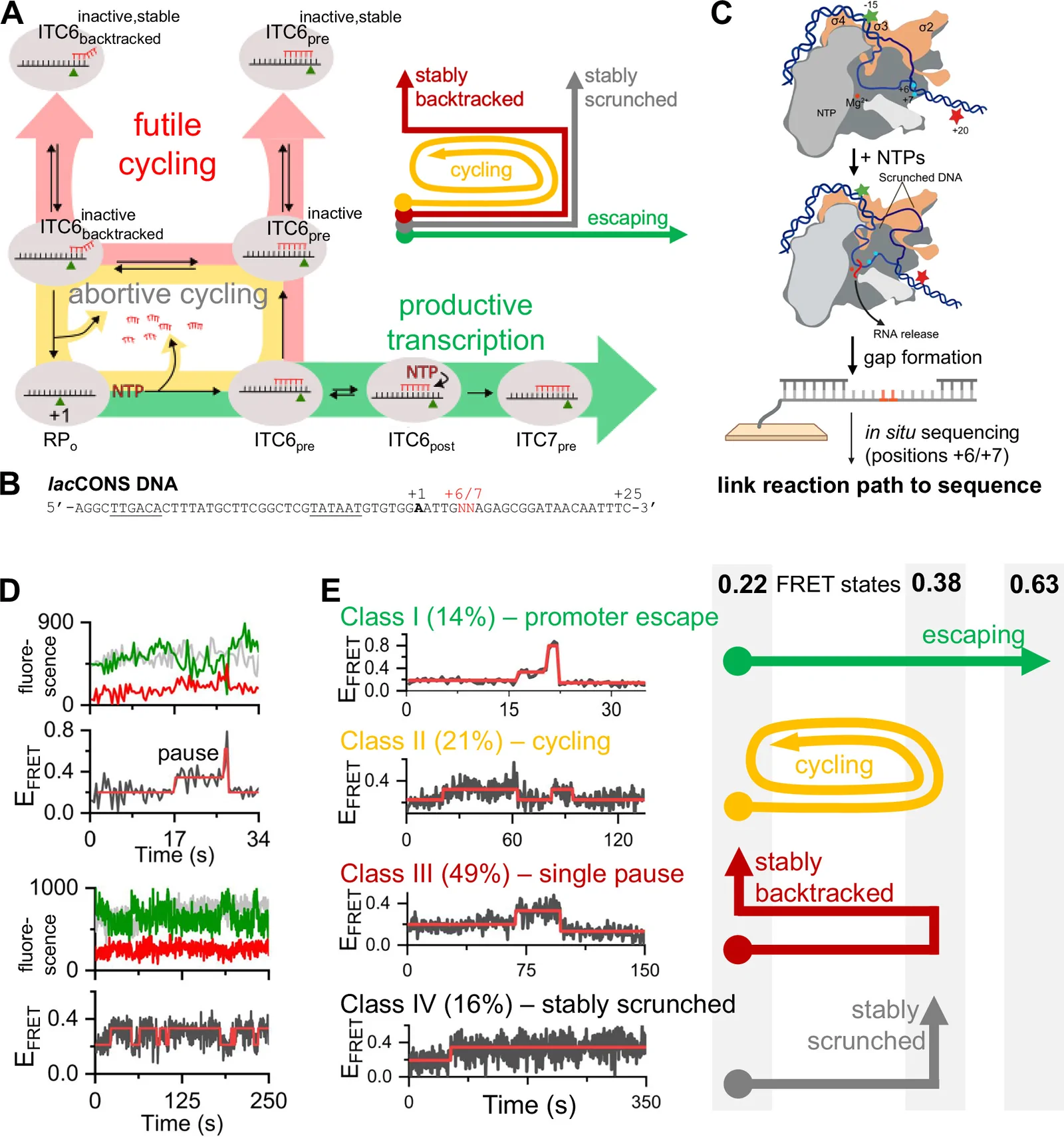
Linking kinetics of protein-driven reactions to DNA sequence: initial transcription mechanism. **A** Schematic of the three main paths for the short RNAs synthesized during initial transcription. Green path: productive RNA extension and promoter escape to elongation. Yellow path: abortive RNA release and restart of RNA synthesis. Pink path: futile-cycling between a stably scrunched and RNA-backtracked state; RNAP tends to pause at ITC6 (initially transcribing complex with 6-nt RNA). Top left: simplified schematic of four main paths during initial transcription. **B** Sequence of the non-template strand of *lac*CONS promoter 16-variant library, generated by randomizing positions +6 and +7 (in red). Underlined sequences: −35 and −10 promoter elements. **C** Transcription complexes on the 16-variant library. After NTP addition to open complexes (top), transcription leads to FRET changes due to movement of the DNA downstream of the transcription start site (middle). After recording FRET changes, the protein and template strand are removed, and positions +6 and +7 are read using competitive inhibition (bottom). **D** Examples of anticorrelated donor (green) and acceptor (red) fluorescence changes, consistent with single-molecule FRET fluctuations. Red line in FRET trace: HMM-based states and transitions. **E** Left, examples of traces belonging to the four main functional classes of active transcription complexes (see main text for descriptions). Right, schematics that map the four main FRET transitions to functional pathways. Some elements in panels (**A**, **C**) were created in BioRender. Kapanidis, A. (2026) https://BioRender.com/bx3ofho. Source data are provided as a Source Data file.

To test whether SPIN-Seq can address complex non-equilibrium reactions, we applied it to initial transcription, largely controlled by sequence-specific interactions between gene promoters and RNA polymerase (RNAP). Single-molecule studies^21–23^ showed that, during bacterial transcription, RNAP pauses after synthesizing a 6-mer RNA, and enters a branched pathway (Fig. 7A) involving a productive path (where the 6-mer is extended to full-length transcripts), an abortive path (where short RNAs dissociate and synthesis restarts), or a “futile-cycling” path (where the 6-mer fluctuates between backtracked and non-backtracked states^22^). The initial transcribed sequence affects initiation pausing^22^; next-generation sequencing (NGS) work on sequence libraries^24^ identified a pause-inducing motif, TNYG (Y: pyrimidine), operational when the A-site of the RNAP active center is at positions +3, +4, +5 and +6 from the transcription start site^25,26^. However, the sequence-dependence of initiation-pausing kinetics and path branching during initial transcription are unknown.

To examine how DNA sequence affects initiation pausing, we generated a library of 16 *lac*CONS promoter variants (Fig. 7B) with degenerate bases at positions +6 and +7 (Methods; Supplementary Fig. 20). To prepare the variant library, a biotinylated and labeled fragment of the transcription non-template (top) strand was annealed to a fragment of the template strand having degenerate positions at positions +6 and +7, and the top strand was extended using Q5 DNA polymerase; the primer-extended product was then ligated to a dsDNA corresponding to positions from +11 to +49 from the transcription start site.

To monitor pausing, we applied an existing single-molecule FRET strategy^21^ by labeling each variant with a donor at position −15 on the non-template strand, and an acceptor at position +20 on the template strand (Fig. 7C). As the acceptor approaches the donor during initial transcription (due to DNA scrunching^21,27^), the apparent FRET efficiency E_FRET_ increases from ~0.2 to ~0.4, where pausing occurs upon synthesis of a 6-mer RNA. Further synthesis past the pause, and promoter escape leads to a transient E_FRET_ of ~0.6 before FRET returns to a low value^21^.

To capture initial transcription kinetics, we formed RNAP-DNA open complexes (RPo) on the promoter variants, immobilized and imaged them (Methods); we observed a mean E_FRET_ of ~0.2, as in our previous work (Supplementary Fig. 21A). After adding 500 µM ApA and 200 µM of all NTPs, most molecules show FRET changes, indicating transcriptional activity (Fig. 7D and Supplementary Fig. 21B); these changes disappeared upon adding rifampicin (an antibiotic that blocks extension beyond a 3-nt RNA), confirming they were specific to transcription (Supplementary Fig. 21C).

After monitoring transcription for ~6.6 min, we removed the protein and template strand, annealed DNAs to create an 8-nt gap centered around positions +6 and +7, and sequenced them using competitive inhibition (Supplementary Fig. 22A–C). We observed that 84% (1370 out of 1633) of the transcriptionally active molecules exhibited a pause at E_FRET_ ~0.38. These pausing molecules displayed 4 main classes of behavior (Fig. 7E), which were characterized by transitions between four main FRET states (Supplementary Fig. 22D, E). Class I molecules (14%) showed stepwise E* changes up to ~0.63 and decreased to a low-FRET state, consistent with promoter escape and completion of transcription on the template^21^. Class II molecules (21%) displayed extensive cycling between the ~0.22 and ~0.38 FRET states, suggesting abortive cycling, during which a short RNA (4- to 6-mer) is released from initial transcribing complexes (ITCs), allowing RNAP to restart RNA synthesis. Class III molecules (49%) showed only a single pause at E_FRET_ ~0.38, after which they return to ~0.22 and stay there for the rest of the trace; these molecules are likely to be inactive ITCs with backtracked RNA^22,23^. Finally, Class IV molecules (16%) reached the ~0.38 FRET state and stayed there for the rest of the trace (~5 min); these molecules are stably scrunched complexes, major intermediates during *lac*CONS initial transcription^21,22^. The convolution of all signals is visualized in heatmaps of FRET temporal evolution as a function of sequence, providing an overview of differences between sequences (Supplementary Fig. 23).

**Fig. 8:**
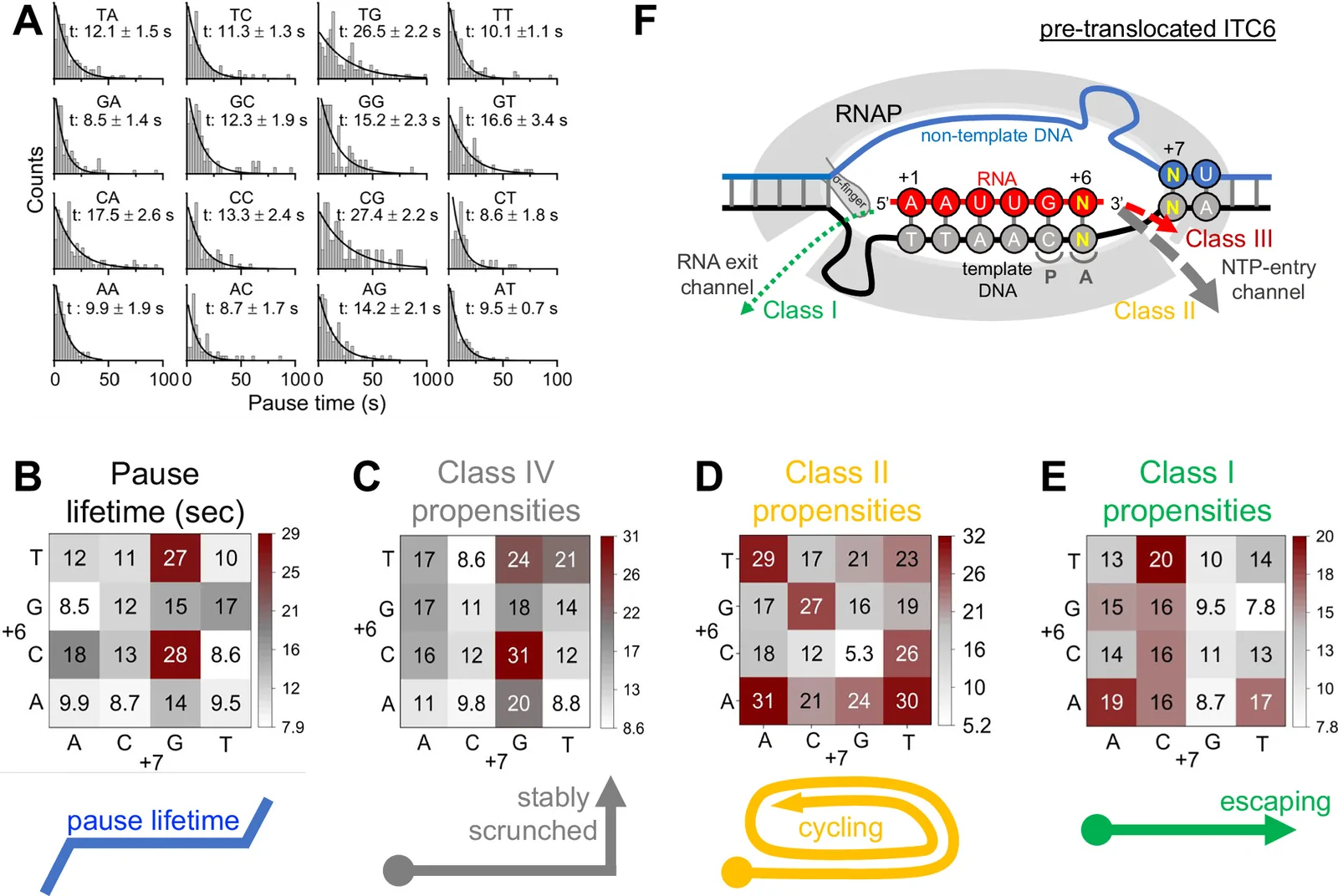
Sequence dependence of pausing kinetics and reaction paths. **A** Distribution of initiation-pause lifetimes, and single-exponential decay fitting to obtain the pause lifetime for each variant. **B** Heatmap of pause lifetimes for different sequences. **C**–**E** Heatmap of propensity for stable-scrunching, cycling, and promoter escape, respectively, for different sequences, shown as percentage of molecules in the population belonging to this class. **F** Schematic of the pre-translocated ITC6 state. The 6-mer RNA (in red) can overcome the barrier of σ-finger to escape to elongation (Class I events), backtrack into the NTP-entry channel and dissociate (Class II events), or backtrack and remain arrested in the NTP-entry channel (Class III events). Class IV events involve an inactive state of ITC6, likely reflecting an inactive RNAP conformation. P and A: P-site and A-site of the RNAP active center. Source data are provided as a Source Data file.

To determine the pause lifetimes of all 16 variants, we measured the duration of each dwell at E_FRET_ ~0.38 for Classes I-III (Fig. 7E), plotted the first-pause dwell-time distributions for each sequence and determined pause lifetimes (Fig. 8A, B). Consistent with earlier work^22,24^, sequence T^+6^G^+7^, found both on the natural *lac* promoter and on synthetic *lac*CONS promoters, showed a long pause (~27 s, *n* = 110). Sequence C^+6^G^+7^ led to similar pausing (~27 s, *n* = 80), while G^+6^A^+7^ showed the shortest pause (~8.5 s, *n* = 88); hence, just a 2-nt sequence can lengthen the pause by >3-fold. In general, promoters with pyrimidines (Y; C or T, the preference being stronger for C) at +6, and a G at +7, exhibited long pausing, supporting an initiation pause element of (C/T)^+6^G^+7^ or Y^+6^G^+7^ (Fig. 8A, B). This sequence matches the consensus for pausing in transcription elongation^25,26^, pausing in initial transcription^24^, and σ-dependent pausing in early elongation^28^, as determined using NGS; in all cases, the pause-inducing sequence preferences reflected the tendency of specific nucleotides to disfavor RNAP translocation from its pre- to post-translocated state, with Y^+6^ preferences reflecting RNAP active-site binding preferences for specific nucleotides, and G^+7^ preferences reflecting effects of duplex stability and active-site binding preferences^26,29^.

To verify our findings, we prepared the C^+6^G^+7^ and A^+6^T^+7^ variants, associated with long and short pausing, respectively. Analyzing the variants separately recapitulated the findings from the library, validating our approach (Supplementary Fig. 24).

We also examined how sequence impacts the transcription profile by looking at the sequence dependence of the tendency to belong to a given class (Fig. 8C–E and Supplementary Fig. 23). Sequences T^+6^G^+7^ and C^+6^G^+7^ (showing the longest pauses in Classes I-III) were linked to stable-scrunching (Class IV; Fig. 8C), strongly suggesting that Y^+6^G^+7^ sequences favor formation of a common reaction intermediate that precedes entry into either a long but transient paused state, or an exceptionally long paused state. The latter intermediate is likely an inactive pre-translocated ITC6 (Figs. 7A and 8F), the branchpoint entry to Class II, III, and IV molecules.

In contrast, cycling molecules (Class II) show a striking preference for A/T at positions +6 and +7 (e.g., 31% of A^+6^A^+7^ traces cycle, where only 5% of C^+6^G^+7^ traces cycle; Fig. 8D). It has been proposed^21,22^ that cycling molecules leave the paused state via RNA backtracking, followed by RNA entry in the NTP-entry channel, RNA release, and restart of RNA synthesis (Fig. 8F). Our results strongly support this proposal, since the observed A/T preference indicates low duplex stability and easier opening of the RNA-DNA hybrid at the transcript 3′-end, matching a well-known determinant for RNA backtracking^30,31^. Notably, many complexes with A/T sequences at positions +6 and +7 will synthesize a 7-mer (since they pause for a shorter time), and have two A/U nucleotides at the RNA 3′-end, while still encountering the σ^70^ finger (a structure blocking the path of growing RNA, shown not to be fully displaced on lacCONS until 10-nt RNAs are synthesized^32^). Efficient cycling also requires that short RNAs dissociate rapidly from the NTP-entry channel to allow synthesis to restart; consistent with this, A/T sequences at positions +6 and +7 are under-represented in single-pause events (Class III; Supplementary Fig. 25), which reflect RNA backtracking into the NTP-entry channel and transcriptional arrest (Fig. 8F).

Finally, promoter-escape events (Class I) disfavor the presence of G at +7, while showing a slight preference for a C at +7, with T^+6^C^+7^ leading to the most productive transcription (Fig. 8E). This preference reflects the fact that “productive” sequences must avoid the long pausing and stable-scrunching favored by a G at +7, and the abortive cycling favored by A/T at +6/+7; as such, our analysis can offer guidance for promoter design.

## Discussion

We introduced SPIN-Seq, a method that leverages protein-free in situ sequencing to link functional and structural phenotypes of single DNA molecules with their sequence. The sequencing aspect of the method relies on direct and highly accurate detection of single DNA mismatches via transient hybridization. Our sequencing approach maintained a high base-calling accuracy (~97%) for all positions within a 5-nt sequence, and remained highly accurate on an all-A sequence, demonstrating its ability to work with A/T-rich sequences.

SPIN-Seq quantifies DNA interactions across a broad range of affinities (sub-nM to 10 μΜ), is applicable to a wide repertoire of DNA-interacting biomolecules, and is compatible with diverse libraries of nucleic acids and DNA-encoded molecules, such as aptamers and DNA-encoded drugs.

### Insight into transcription mechanisms

Our work demonstrates that even a modest library of 16 DNAs offers rich mechanistic insight into a complex non-equilibrium reaction, showing directly that just two nucleotides can dramatically alter transcription initiation pausing and the distribution between productive and non-productive paths. We also reveal that initial transcription pausing has further similarities with elongation pausing, with additional tuning contributed by initiation factors and by different extents of DNA melting and scrunching. Given the homology of bacterial and eukaryotic RNAPs, sequence-dependent branching may also shape eukaryotic transcription, where a finger-like element in TFIIB causes initiation pausing at positions +7 to +9^33^, complementing regulation through promoter-proximal pausing^34^.

### Compatibility with other biological systems

Although both systems studied here were involved in bacterial transcription, the method is fully compatible with a much wider repertoire of systems and interactions, including proteins, nucleic acids, and small molecules. These include other types of protein interactions with nucleic acids (e.g., binding of eukaryotic DNA-binding proteins on nucleosomal DNA templates), interactions of nucleic-acid aptamers with their targets, and interactions of RNAs with RNA-binding proteins.

The prerequisite for SPIN-Seq is the ability to prepare a variant library compatible with the functional assay, and to form a DNA gap around the variable region after the functional assay is complete. If the DNA variant sequence is processed chemically during the interaction with a protein (e.g., due to protein-driven DNA cleavage or DNA modification) and thus cannot be used for gap-based sequencing, sequencing can be performed first, followed by the formation of the reaction substrate on the surface, followed by the local chemical reaction.

Extensions of SPIN-seq can also analyse epigenetic modifications, e.g., by bar-coding DNA variants that carry different sequences and modifications to create a coded variant library, performing the functional assay, and then decoding the individual molecules to connect sequence and modification to function. In general, the use of bar-coding will enable exploration of sequence complexity much beyond what is available by randomizing short DNA regions.

### Comparison with MUSCLE/SPARXS

As we show by analyzing the interaction of a transcription factor with its cognate DNA site, SPIN-Seq uses standard glass surfaces for single-molecule imaging. This addresses a limitation of multiplexed single-molecule methods that rely on MiSeq chips^10,11^, which are surfaces not optimal for imaging labeled proteins. Further, the ability to use standard glass surfaces removes the need to re-optimize existing surface immobilization and functional assay protocols for use on MiSeq surfaces, and decouples the multiplexed assay from the commercial availability of MiSeq chips (which have recently been discontinued). Use of SPIN-Seq also removes the need for DNA amplification and for sequencing on a separate instrument, steps required for MUSCLE/SPARXS.

Regarding throughput, SPIN-Seq has a lower throughput (see also next section) compared to MUSCLE/SPARXS when examining dynamics of DNA structures and complexes, since the latter methods rapidly scan large areas of DNA molecules and subsequently leverage a well-developed commercial technology that produces a massive number of DNA reads. However, SPIN-Seq and MUSCLE/SPARXS are expected to have the same throughput when studying non-equilibrium reactions, due to the confinement of such studies (for both types of methods) to a single field-of-view (FOV) per surface interrogation. This issue has also been extensively discussed in a recent review on sequence-multiplexed single-molecule methods^35^.

### Throughput and extensions

Currently, SPIN-Seq examines a single FOV of 120 × 50 μm for functional activity and DNA sequence. For the systems we studied, out of 2000–2500 detected molecules per FOV, 300–600 molecules provide usable time-series for both function and sequence (with the remaining molecules representing mainly free DNA, inactive species, or low signal-to-noise events). For a 16-variant library, the assay takes ~1 h/FOV (with ~40 min required for 2-nt sequencing), offering a coverage of ~100 molecules per variant in four replicates (~4 h microscopy time). To cover a larger library (e.g., 1024 sequences) at read depth of ~50 molecules per sequence (sufficient for identifying patterns of reactions and performing basic kinetic analysis), ~50,000 usable molecules per library will be required. With the current throughput, this is possible by working at lower read depth (e.g., 10 molecules per sequence), which can still provide useful information if sequences are pooled; however, pooling complicates seeing the effect of individual sequences on function.

We foresee several ways to cover large libraries. Automated microfluidics will allow operation for extended periods, as shown for other multiplexed methods^10,11^. Gap interrogation can be shortened; in fact, 50% shortening of that step still maintained both high molecular throughput and high base-calling accuracies (>98%; Supplementary Fig. 26A). Shortening the interrogation time will also help address a small, time-dependent loss of molecules (~3% after 40 min of interrogation; Supplementary Fig. 26B–D), most likely due to photo-induced depletion of gapped substrates, an issue in DNA-PAINT^36^. Additional improvements include multi-color versions to read bases in a single interrogation, and imaging larger FOVs that can capture up to 15,000 molecules per FOV^37^. For transient protein binding or cycling reactions, throughput can be increased by working at much higher densities of gapped molecules, which are still resolvable using high-precision localization^13,38^, already present in our analysis (Figs. 2I and 4D). SPIN-Seq can also operate by sequencing only functionally important molecules of large libraries, e.g., by scanning the surface rapidly to identify the top 5–10 functionally important molecules, sequence only these molecules, and use their features to further engineer a library.

SPIN-Seq can also be integrated with other single-molecule approaches that incorporate fluorescence, broadening opportunities for biophysical analysis. Together with recent single-molecule multiplexed methods^10,11,35,39–41^, SPIN-Seq offers a powerful framework to systematically decode how DNA sequence shapes molecular dynamics, interactions and mechanisms.

## Methods

### Preparation of DNA fragments

A complete list of all DNA fragments is found in Supplementary Table 1. Oligodeoxyribonucleotides (“oligos”) used in Fig. 2 were synthesized on an Applied Biosystems 394 automated DNA/RNA synthesizer using a standard 1.0 μmole phosphoramidite cycle of acid-catalyzed detritylation, coupling, capping, and iodine oxidation. Stepwise coupling efficiencies and overall yields were determined by the automated trityl cation conductivity monitoring facility and were >98.0%. All β-cyanoethyl phosphoramidite monomers were dissolved in anhydrous acetonitrile to a concentration of 0.1 M immediately prior to use, with a coupling time of 50 s for normal A, G, C, and T monomers and was extended to 600 s for 5′-amino, 5′-biotin and 5-amino C6 dT phosphoramidite monomers (from Link Technologies Ltd.). 5′-Amino and internal 5-amino-C6-dT modified oligonucleotides on resin were treated with a solution of diethylamine (10% in acetonitrile) for 20 min to selectively remove the cyanoethyl protecting groups. Cleavage and deprotection of oligonucleotides were achieved by exposure to concentrated aqueous ammonia solution for 60 min at room temperature, followed by heating in a sealed tube for 5 h at 55 °C.

Oligonucleotides labeled with either an ATTO647N or Cy3B were prepared by NHS-ester coupling. The unlabeled precursor oligonucleotide (40 nmol) was freeze-dried and resuspended in sodium bicarbonate buffer (18 μl, 0.5 M, and pH 8.5). Separately, Cy3B or ATTO647N NHS-ester (6 μl, 20 nmol/μl in DMSO) was mixed with DMSO (6 μl). The NHS-ester solution was then added to the oligonucleotide solution and mixed vigorously before shaking (800 rpm, room temperature). After 3 h, reactions were diluted with water (970 μl) and purified by desalting on a disposable gel-filtration column (NAP10, GE Healthcare Life Sciences, cat. no. GE17-0854-02) according to the manufacturer’s instructions to remove most of the free dye. Labeled oligonucleotides were further purified by reverse-phase HPLC using a Gilson HPLC system with ACE® C8 column (10 mm × 250 mm, pore size 100 Å, particle size 10 μm) with a gradient of buffer A (0.1 M TEAB, pH 7.5, where TEAB is triethylammonium bicarbonate) to buffer B (0.1 M TEAB, pH 7.5 containing 50% v/v acetonitrile) and flow rate of 4 ml/min over a total run time of 17.5 min. For all ATTO647N-labeled oligonucleotides, a gradient of 70–100% buffer B was used. For Cy3B-labeled oligonucleotides, a gradient of 20–35% buffer B was used. The TEAB buffer and acetonitrile in the collected fractions were removed in vacuo or by freeze drying.

All oligonucleotides were characterized by negative-mode electrospray using a UPLC-MS Waters XEVO G2-QTOF mass spectrometer and an Acquity UPLC system with a BEH C18 1.7-μm column (Waters).

Oligonucleotides used in all remaining figures were procured from Metabion and Biomer Corporation. Commercially procured oligonucleotides were dissolved in nuclease-free water to a final concentration of 100 μM and stored at −20 °C.

With regard to our DNA nomenclature (Table S1), in a strand named Gap-(1–27)-Top^Cy3B,18^, “Gap” refers to the aim of the experiment (here, sequencing experiments with a gapped DNA); the term “(1–27)” refers to the start and the end of the strand with respect to the 5′ end, “Top” denotes the top strand, and the superscript (^Cy3B, 18^) specifies a Cy3B fluorophore attached to position 18. For ease of reference and brevity, we also use a shorter abbreviation for DNAs, which denotes the type of seal (or gap); the length of the seal (or gap); and the gap position and base interrogated. For example, “U8-A^3^” refers to a U-seal that is 8-nt long and has an A base at the position that corresponds to gap position 3 in the gap (i.e., interrogating for the presence of a T in the gap).

To form gapped-DNA substrates, as well as fully duplexed DNA substrates, oligonucleotides were annealed by mixing complementary strands in a ratio of 1:1 in hybridization buffer (200 mM Tris-HCl, pH 8.0, 500 mM NaCl, 1 mM EDTA). The mixture was heated to 95 °C for 5 min to denature the strands, followed by gradual cooling to 25 °C at a rate of 2 °C per min to facilitate annealing. The annealed DNA strands were then placed on ice until further use.

### Preparation of CAPcons DNA library

To prepare the variant library for position 5 of the 22-bp CAPconsensus sequence (CAPcons), 1 µM of biotinylated 49-nt ssDNA having a degenerate base at position 25 (CAPcons-(1–49)-N^25^-B^Bio,49^) was annealed with 2 µM of complementary Cy3B-labeled 20-nt ssDNA (CAPcons-(1–20)-Top^Cy3B,11^). The top strand was then extended using Q5 DNA Polymerase (Thermo Fisher Scientific) and dNTPs (NEB) using melting, annealing and extension temperatures of 95, 62, and 72 °C, respectively. The variant library was purified using the QiAquick purification kit (QIAGEN). Success of the primer extension was verified by running the purified product in a 20% bi-acrylamide/acrylamide gel and imaged using a Typhoon gel scanning system. The band corresponding to the 49-bp dsDNA was excised from the gel and soaked in TE buffer overnight at room temperature. To concentrate the product, the DNA was precipitated by mixing the solution with 3X volume of ice-cold ethanol, 1 µl of 1 mg/ml RNA-grade glycogen (Thermo Fisher Scientific) and 20 mM sodium acetate. The mixture was kept at −20 °C for 4 h, followed by centrifugation and removal of the supernatant. The precipitated product was dissolved in 50 µl of assay buffer (AB: 20 mM HEPES pH 7.7, 80 mM MgCl_2_, 200 mM NaCl) and was stored at −20 °C. The final DNA concentration was measured using UV-Vis spectrophotometry on a Nanodrop ND1000 spectrophotometer.

### Preparation of lacCONS DNA library

To prepare the variant library for positions +6 and +7 of *lac*CONS promoter (Supplementary Fig. 20), 2 µM of a biotinylated and labeled fragment of the transcription non-template strand (lacCONS(−39/ −10)-Top^Cy3B,-15; Bio,-39^) was first annealed with 1 µM of a fragment of the template strand having degenerate positions at positions +6 and +7 (lacCONS(−39/ +10)-N^+6^N^+7^-B). The top strand was then extended using Q5 DNA polymerase (Thermo Fisher Scientific) and dNTPs (NEB), using melting, annealing and extension temperatures of 95, 62, and 72 °C, respectively. A second fragment of the template strand (lacCONS(+11/ +49)-B^Atto647N,+20^) was then annealed to a second fragment of the non-template strand (lacCONS(+11/ +49)-Top), and the annealed strand was ligated to the primer-extended product using 0.5 µl of 2,000,000 units/ml T4 DNA ligase (NEB) and incubating overnight at 16 °C. Success of the primer extension was verified by running the purified product in a 20% bi-acrylamide/acrylamide gel and imaged using a Typhoon gel scanning system. The band corresponding to 86-bp sequences containing both Cy3B and ATTO647N fluorophores was excised from the gel and soaked in TE buffer overnight. The gel-extracted product was precipitated by mixing the solution with 3X volume of ice-cold ethanol, 1 µl of 1 mg/ml glycogen, and 20 mM sodium acetate, and keeping at −20 °C for 4 h. The precipitated DNA was finally dissolved in 50 µl of AB and stored at −20 °C.

### Preparation of labeled CAP derivative (Alexa647-CAP)

We used a CAP derivative with two reactive Cys residues (Cys17; Ser178), that has been extensively characterized in terms of function and labeling^1,2^. CAP overexpression and purification was performed using plasmid pHSCRP-His6-H17CC178S [constructed from plasmid pAKCRP-His6^3^ using site-directed mutagenesis (Q5 Site-Directed Mutagenesis Kit, NEB)]. Fluorescence labeling was performed by incubating 20 μM CAP-(Cys17; Ser178) with 400 μM Alexa647 maleimide in buffer A [40 mM HEPES-NaOH pH 7.3, 100 mM NaCl, 2 mM TCEP and 5% glycerol] for 12 h on ice. The fluorescently labeled protein (Alexa647-CAP) was purified using a G25 Sephadex gel-filtration column pre-equilibrated with buffer B (40 mM Tris-HCl pH 8, 100 mM NaCl, 0.1 mM TCEP and 5% glycerol), followed by a purification step on Ni:NTA agarose affinity column (and elution with 1 M imidazole in buffer B), followed by buffer exchange (five cycles of concentration to 0.5 ml volume and dilution with 5 ml buffer B) using a 3 kDa MWCO Amicon Ultra-15 centrifugal ultrafilter (Millipore). The purified Alexa647-CAP was stored in aliquots at −80 °C. The labeling efficiency was 40%. The sequence-specific DNA-binding activity for the CAP dimer (measured using EMSA assays) was ~20%, within the range of 15–25% typically obtained for purified and labeled CAP^4,5^.

### Preparation of RNA polymerase holoenzyme

*Escherichia coli* RNAP holoenzyme was expressed and purified by co-expressing genes of RNAP β, β’, α, ω, and σ^70^. A primary culture of 20 mL LB broth was inoculated using a single colony of BL21 DE3 *E. coli* strain transformed with plasmid pV10 ^6^, which encodes the *E. coli rpoA-rpoB -rpoC* [His6] and *rpoZ* ORFs under control of T7 promoter, and plasmid pRSFduet-sigma^7^. Ampicillin (100 μg/ml) and kanamycin (50 μg/ml) were used as selection antibiotics. A second culture (2 × 1 L) was started using 10 mL of primary culture, using the same antibiotic level and was grown at 37 °C until O.D. reached 0.6. Inducer IPTG was added at 1 mM, and the cultures were further incubated at 37 °C for 3 h. Cells were harvested and lysed, followed by precipitation of the cell lysate using polyethyleneimine and ammonium sulfate. Purification of the protein was performed using a Ni-NTA agarose affinity column with elution using 200 mM imidazole in buffer A. The affinity-purified sample was passed through anion-exchange chromatography equipped with Mono-Q 10/100 GL. Fractions containing the protein of interest were pooled together and concentrated using a 30 kDa MWCO Amicon Ultra-15 centrifugal ultrafilter. The activity of this RNAP preparation has been characterized extensively previously^8–10^.

### Preparation of surface-immobilized DNA for imaging

Microscope coverslips and slides were prepared essentially as described (6). Briefly, borosilicate coverslips (#1.5 Menzel/Thermo Fisher) were first burned in a furnace at 500 °C, and were plasma-cleaned; subsequently, the coverslips were treated sequentially with acetone, 2% Vectabond aminosilane (Vector Labs) in acetone, and water, dried under nitrogen, and bonded to 6-mm silicone gaskets (GBL103280; Grace Bio-Labs), creating observation wells with aminosilane-functionalized glass. Wells were coated with 20 µl of 30 mM mPEG-SVA (Laysan Bio) and 0.75 mM Biotin-PEG-SVA (Laysan Bio) in MOPS-NaOH (pH 7.5) for 90 min at 22 °C, washed with PBS, then treated with 30 μl of 10 μM NeutrAvidin (Thermo Fisher) in 0.5x PBS for 10 min at 22 °C. After PBS washes, wells had NeutrAvidin-biotin-PEG/mPEG-functionalized glass floors. The biotinylated DNA constructs were added to the observation wells to a final concentration of 50 pM, and were incubated until achieving an immobilization density of ~0.2 molecules per μm^2^, and then were washed with AB. Next, we added to the observation wells 40 µl of FluoSpheres™ (Biotin-Labeled Microspheres, 0.2 μm, yellow-green fluorescent (505/515), 1% solids) diluted to 1:2000 volume in AB as fiducial markers, and incubated for 20–40 s. We then washed the wells three times with AB, added 20 µL of 50 pM biotinylated gapped-DNA constructs in imaging buffer 1 (IB1: 20 mM HEPES, pH 7.7, 200 mM NaCl, 80 mM MgCl_2_, 6 mM BSA, 1 mM TROLOX, 1% glucose, 40 µg/ml catalase and 0.1 mg/ml glucose oxidase; for a list of all assay buffers, see Table S4), incubated for 10–30 s, until achieving an immobilization density of 0.2 molecules per µm^2^ on average and washed three times with 200 µl of AB.

To surface-immobilize DNAs for single-base sequencing in a mixed population with prior information (Fig. 2E), four different 8-nt DNA-gapped molecules were sequentially immobilized on the surface at a concentration of 50 pM. First, we immobilized Gap8-A^3^ (prepared by annealing Gap-(1–65)-A^30^-B^Bio,1^, Gap-(1–27)-Top^Cy3B,18^, and Gap-(36–65)-Top) and registered the locations of the DNA molecules by exciting with 0.2 mW of 532 nm laser, followed by photobleaching using 532 nm laser at 2 mW power. We then immobilized Gap8-T^3^ (prepared as above but using Gap-(1–65)-T^30^-B^Bio,1^ as a bottom strand) registered the DNA locations, and photobleached the fluorophores. We repeated the same procedures for Gap8-G^3^ and Gap8-C^3^. We then interrogated the gapped molecules using four rounds of transient seal hybridization.

### Single-molecule imaging

All single-molecule measurements were performed using a Nanoimager-S microscope (Oxford Nanoimaging), equipped with 100x 1.4 NA objective, and a sCMOS camera (Orca Flash4 v3). The emission channel was split into two channels, one corresponding to wavelengths 498–551 nm and 576–620 nm, and the other corresponding to a 685/40 nm band (using 640LP channel splitter dichroic). An objective-based total internal reflection fluorescence (TIRF) illumination mode at a TIRF-lens setting angle of 56˚ (which corresponds to TIRF excitation conditions) was used. All sequencing-related imaging experiments were carried out with continuous-wave excitation of 532 nm laser at 0.2 mW power, and 638 nm laser at 0.6 mW power, using a field-of-view of ~80 μm × 50 μm. For the experiments in Fig. 2A, B, 100-ms exposures were used; for experiments in Fig. 2E, the localization image was collected using 200-ms exposures, and the sequence-interrogation movies using 300-ms exposures for 1000 frames. For Figs. 4–6 and 5–19, all movies (1200 frames) and localization images were collected using 250-ms exposures. For Fig. 7 and Supplementary Figs. 21–25, 532/638 nm alternating laser excitation and 200-ms exposures for 1000 frames were used.

### Real-time in situ sequencing assay

To call individual bases, we immobilized gapped-DNA molecules on the surface and registered their locations by exciting them with a 532-nm laser and detecting them using 200-ms exposures. In a non-competitive approach of sequencing, 40 µl of 100 nM labeled seal was added on the surface in imaging buffer 2 (IB2: 20 mM HEPES, pH 7.7, 200 mM NaCl, 80 mM MgCl_2_, 5% dextran sulfate, 10% formamide, 1 mM TROLOX, 1% glucose, 40 µg/ml catalase, and 0.1 mg/ml glucose oxidase). High MgCl_2_ concentrations increase DNA duplex stability^12^ and use of 5–10% dextran sulfate as a crowding agent accelerates DNA hybridization^13,14^. The molecules were then excited using 532/638 nm laser excitation, and 1000 frames of 300-ms exposures were collected. Next, the surface was washed using 40 µl of AB three times and incubated for 1 min with 40 µL of 100 nM of the labeled seal featuring a different standard base at the interrogated position; we then collected 1000 frames as described above. We repeated the process for the remaining two labeled seals.

### Optimization of sequencing by competitive inhibition

To optimize the seal concentrations for reading one base using competitive inhibition (Supplementary Fig. 6), we used an R-seal that comprises an 8-nt ATTO647N-labeled oligo with a degenerate base complementary to gap position 3 (R8-N^3^; Supplementary Table 1). Binding of the R-seal to an 8-nt gapped DNA (Gap8-G^3^, which features a G base at gap position 3, and was studied in Fig. 2A) leads to the appearance of fluorescent signals (Fig. S6; see points where no U-seal was added).

To optimize the concentration of the U-seal complementary to the 8-nt gap (seal U8-C^3^, fully complementary to Gap8-G^3^), we monitored the gap-binding of 100 nM R8-N^3^ in the presence of 100–600 nM U8-C^3^ in imaging buffer 3 (IB3: 20 mM HEPES, pH 7.7, 200 mM NaCl, 80 mM MgCl_2_, 10% dextran sulfate, 10% formamide, 1 mM TROLOX, 1% glucose, 40 µg/ml catalase, and 0.1 mg/ml glucose oxidase). Competition due to increasing U8-C^3^ leads to gradual loss of R-seal binding, as seen in fluorescence time-traces (Supplementary Fig. 6A), and in the markedly decreased R-seal dwell-times in the gap (which provides evidence for “active” displacement of the R-seal from the complementary U-seal; Supplementary Figs. 6B, C), and in the markedly decreased mean number of R-seal localizations in the gap (Supplementary Fig. 6D). To compare the stability of different gap-seal pairs, we used the mean lifetime of the bound state, obtained from the first moment of the dwell-time distribution and accounting for both kinetic components and their relative populations: $< \tau > ={\int }_{0}^{\infty }{tP}(t){dt}=\frac{{a}_{1}{\tau }_{1}+{a}_{2}{\tau }_{2}}{{a}_{1}+{a}_{2}}$. To compare the competition due to the complementary U-seal with the competition due to the U-seal with a 1-nt mismatch to the 8-nt gap (seal U8-T^3^), we monitored the gap-binding of 100 nM R-seal in the presence of 100–600 nM U8-T^3^ in IB3 (i.e., same concentration range as for U8-C^3^). R-seal binding also decreases with increasing U8-T^3^ (Supplementary Fig. 7) but to a much lower degree. We chose to use 100–200 nM R-seal and 300–600 nM U-seal (i.e., a 3:1 concentration ratio) for the competitive-inhibition assays, since they offer optimal contrast between complementary and 1-nt mismatched U-seals.

To optimize the seal concentrations for reading three bases using the competitive inhibition assay, we used a 13-nt gapped DNA (Gap13-G^5^G^6^C^7^, which features G, G, and C at gap positions 5, 6, and 7), prepared by annealing Gap-(1–65)-B^Bio,1^ with Gap-(1–27)-Top^Cy3B, ^^25^ and Gap-(41–65)-Top. We also used an R-seal that comprises a 13-nt ATTO647N-labeled oligo with three degenerate bases complementary to gap positions 5, 6, and 7 (R13-N^5^N^6^N^7^; Supplementary Table 1). Since the 13-nt R-seal contains three degenerate bases (leading to the effective concentration of the exact complementary seal sequence in the mixture to be 64-fold lower than the total concentration used), we increased the R-seal concentration to 500 nM; this was possible using dark quencher BHQ1, which suppresses ATTO647N fluorescence until the R-seal binds to the gap (7). Since binding of a 13 nt R-seal will lead to longer dwells and more stable seal binding, this increased stability may lead to information loss (due to fluorophore bleaching while the seal is stably bound), to difficulties in discriminating between stably hybridized molecules and non-specifically surface-adsorbed molecules, and to the need for more extensive washing for seal removal for each interrogation. Making binding more transient addresses these issues; we thus decreased the R-seal binding stability by increasing the formamide concentration. Since too much destabilization makes binding difficult to detect, we tested R-seal binding in the presence of 10–25% formamide in IΒ3 (Fig. S10), and identified 15–20% formamide as the optimal concentration range for R-seal sampling, since it maximizes the number of binding events while keeping the dwell time detectable with our current temporal resolution.

To optimize the concentration of the U-seal complementary to interrogated position 5 in the 13-nt gap (seal U13-C^5^N^6^N^7^), we followed the same optimization procedure as for the 1-nt competitive inhibition assay, and we monitored the gap-binding of 500 nM R-seal in the presence of 0.5–3.5 μM U-seal in IB3 with 15% formamide (Supplementary Fig. 11A) and observed a substantial decrease in R-seal binding at 0.5 μΜ U-seal and a more gradual decrease thereafter (Supplementary Fig. 11B–D). After performing an analogous experiment with the 1-nt mismatched U-seal (U13-T^5^N^6^N^7^; Supplementary Fig. 12), we observed that significant R-seal binding remains at 1.5 μM U-seal concentration. We thus chose to use 500 nM R-seal and 1.5 μM U-seal for the competitive inhibition assay, since they offer optimal contrast in R-seal binding between complementary and 1-nt mismatched U-seals.

To optimize the seal concentrations for reading five bases using competitive inhibition, we used the same gapped DNA as for 3-base sequencing. We also used an R-seal that comprises a 13-nt ATTO647N-labeled oligo with five degenerate bases complementary to gap positions 5, 6, 7, 8, and 9 (R13-N^5^N^6^N^7^N^8^N^9^, Supplementary Table 1). Since the 13-nt R-seal contains five degenerate bases (leading to the effective concentration of the exact complementary seal sequence in the mixture to be 1024-fold lower than the total concentration used), we increased the R-seal concentration to 1 μM while maintaining the same buffer conditions. To optimize the concentration of the U-seal complementary to interrogated position 5 in the 13-nt gap (seal U13-C^5^N^6^N^7^N^8^N^9^), we monitored the gap-binding of 1 μΜ R-seal in the presence of 1–5 μM U-seal (Supplementary Fig. 14A) and observed a substantial decrease in R-seal binding at 1 μΜ U-seal and a more gradual decrease thereafter (Supplementary Figs. 14B–D). After performing analogous experiments with the 1-nt mismatched U-seal (U13-T^5^N^6^N^7^N^8^N^9^; Supplementary Fig. 15), we observed that optimum contrast is achieved at 2 μM mismatch U-seal concentration. We thus chose to use 1 μM R-seal and 2 μM U-seal (a 1:2 concentration ratio) for the competitive inhibition assay.

### Sample preparation for real-time CAP–DNA interactions

CAPcons DNA was prepared by annealing a 5′-biotinylated ssDNA (CAPcons-(1–49)-C^25^-B^Bio,49^) with its complementary ssDNA (CAPcons-(1–49)- G^25^-Top^Cy3B,13^) containing Cy3B at position 13; the Cy3B fluorophore allowed detection and localization of the DNA molecules on the surface. To prepare the G:C variant at position 5 of the CAPcons sequence, we altered the base at the 25th position of the biotinylated ssDNA sequence to G (CAPcons-(1–49)-G^25^-B^Bio,49^) and annealed with its complementary ssDNA sequence (CAPcons-(1–49)-C^25^-Top^Cy3B,13^).

To study CAP–DNA interactions in real-time (Fig. 6B, C), we immobilized one or more CAPcons variants on the surface by incubating 50 pM of 40 µl DNA on a PEGylated surface and washing three times with AB, followed by two washes with CAP-binding buffer (40 mM Tris, pH 8, 100 mM KCl, 10 mM MgCl_2_, 5% glycerol, and 0.2 mM cAMP). We then added 1.25 nM AF647-CAP, and imaged binding in CAP binding buffer supplemented with 1 mM TROLOX, 1% glucose, 40 µg/ml catalase and 0.1 mg/ml glucose oxidase. We used 250-ms exposures and collected 1800 frames.

### In situ DNA gap preparation and sequencing

To sequence the CAPcons variant library after the CAP-binding assay, we removed the non-biotinylated top strand, and created a gap around the positions of interest. We washed the surface three times using MQ water, incubated the surface with 20 mM NaOH for 30 s to de-hybridize the dsDNA, and washed three times with AB. To create a 9-nt gap around position 25, flanking strands CAPcons-(30–49)-Top and CAPcons-(1–20)-Top^Cy3B,11^ were incubated on the surface for 15 min at a final concentration of 2 µM in AB supplemented with 10% dextran sulfate.

To measure the efficiency of in situ formation of DNA gaps, we first deposited the 8-nt gapped DNA used in Fig. 2A and localized the gapped DNA molecules (Supplementary Fig. 18A). After performing transient hybridization with the complementary seal, we removed the strands flanking the gap by washing the surface three times with MQ water, incubating it with 20 mM NaOH for 20 s, and washing it three times using AB. We reformed the DNA gaps in situ by incubating the surface with 100 nM of flanking strands Gap-(1–27)-Top^Cy3B,18^ and Gap-(36–65)-Top) in AB supplemented with 10% dextran sulfate for 15 min, and imaged the surface again (Supplementary Fig. 18B). Colocalization analysis between the initial localization image (Supplementary Fig. 18C) and the re-annealed image shows a ~70% colocalization (at a distance threshold of 7 pixels) of the initially deposited DNA gaps and the in situ-formed DNA gaps. Non-colocalized molecules in the in situ-formed gaps likely stem from non-specific binding of the Cy3B-modified flanking strand on the surface, or the absence of the Cy3B-containing flanking strand from the initially deposited DNA gap. To test this, we determined what fraction of a pair of flanking strands on a surface-immobilized DNA gap reforms after a cycle of strand removal and reannealing. For this, we first formed the gapped DNA in situ by annealing the bottom DNA strand with the two flanking strands, each labeled with a spectrally distinct dye (Cy3B and Atto655), imaged the resulting hybrids using 532/640 nm excitation, and performed colocalization analysis. We then removed the flanking strands (by incubating with 20 mM NaOH for 30 s, followed by washing) and re-annealed the labeled flanking strands (incubating for 15 min with 100 nM of each strand in assay buffer with 10% dextran sulfate), followed by imaging. The colocalization between green and red channels was 72% for imaging round 1 and 68% for imaging round 2. However, the cross-colocalization efficiency of gap molecules showing green-red colocalization in both imaging rounds was 94% (Supplementary Fig. 18D), even at 100 nM flanking strands; this value represents the true efficiency in forming the DNA gap in situ.

Since the actual SPIN-Seq experiment uses 10- to 20-fold higher concentration of flanking strands than in the characterization experiment here (with the latter being limited to 100 nM due to the presence of a label on the flanking strand), we conclude that, during in situ DNA gap formation using unlabeled flanking strands, 95–99% of DNAs are converted to gapped-DNA molecules.

To verify the functionality of the in-situ-formed DNA gap, we performed transient hybridization of the complementary seal on the in-situ-formed DNA gap molecules and observed similar seal binding for the preformed and in-situ-formed DNA gap molecules (Supplementary Fig. 18E).

To read position 25 using competitive inhibition, we used sequential interrogations with 200 nM of R-seal R9-CAP-N^5^-Cy5 and 600 nM of each of the 4 U-seals in IB3. In each round of interrogation, we collected 1200 frames using 250-ms exposures.

### Preparation and single-molecule imaging of transcription complexes and reactions

Open complexes were formed by incubating 100 nM of the lacCONS DNA variant library with 150 nM RNAP holoenzyme in KG7 buffer (40 mM HEPES, pH 8, 100 mM potassium glutamate, 10 mM MgCl_2_, 1 mM DTT, 100 µg/mL BSA, 5% glycerol) at 37 °C for 30 min. Subsequently, 1 μl of 1 mg/mL heparin was incubated in the solution for 1 min, followed by centrifuging for 2 min at 2000 RPM. The supernatant was collected in a separate centrifuge tube, and 0.5 μl of the open complex mixture was incubated on a neutravidin-coated slide having 30 μL of KG7 as prepared for single-molecule DNA imaging. After washing off the unbound open complex from the surface, we incubated the surface with 500 μM ApA dinucleotide (Jena Bioscience) diluted in KG7 buffer for 10 min followed by adding an oxygen scavenging system (1% glucose, 40 µg/ml catalase, and 0.1 mg/ml glucose oxidase). We started imaging the complexes using 532/638 nm ALEX excitation and exposures of 200 ms; we then added 200 μM NTPs in real-time using an in-house-made fluidic system equipped with magnetically controlled tubing positioner at ~15 s after starting imaging. We tracked the signal for 6.6 min, followed by washing the surface three times with 40 µl of MQ H_2_O.

### In situ DNA gap preparation and sequencing after transcription assay

After imaging transcription reactions, we washed the surface with MQ H_2_O, added 40 µL of 20 mM freshly prepared NaOH for 30 s to de-hybridize the dsDNA, and washed the surface three times with 40 µl of AB. To create an 8-nt gap around the sequence of interest (positions +6 and +7), two flanking sequences of 39-nt and 41-nt (lacCONS(+11/+49)-B and lacCONS(−39/+2)-B) were incubated on the surface for 15 min at 2 µM final concentration in IB3. Sequencing was performed using 200 nM of 8-nt R-seal and eight sequential interrogations using a mixture of 200 nM R-seal (R8-lacCONS(+3/+9)-N^+7^N^+6^-B^A647N^) and 600 nM of each of the 8 U-seals (Supplementary Table 1) in IB4 (20 mM HEPES pH 7.7, 150 mM MgCl_2_, 200 mM NaCl, 10% dextran sulfate, 5% formamide, 200 µM of freshly prepared spermidine hydrochloride, 1 mM TROLOX, 1% glucose, 40 µg/mL catalase, 0.1 mg/mL glucose oxidase).

### Image and time-series analysis

For all movies, drift correction was performed by first identifying localizations of the fiducial markers (beads) on the surface using in-house Python algorithm (https://github.com/Z-Qing/GapSeq-repo), followed by applying the AIM algorithm^16^ to calculate the drift, and shifting the movies in the opposite direction to compensate for it. Acceptor and donor channels in the movies were aligned to the donor channel of the localization movie using the pystackreg library^17^ with a rigid-body transformation. The transformation matrix between donor channels, as well as the transformation between the donor and acceptor channels, was computed using fiducial markers from the initial frames.

The aligned and undrifted movies were further analyzed using in-house software *napari-molseeq* (https://github.com/piedrro/napari-molseeq). First, gapped-DNA molecules were localized using the integrated Picasso algorithm^18^ with a gradient of 500 and a bounding box size of 5 pixels. For all gapped-DNA localizations, seal DNA binding traces were determined in a bounding box mask of 4 pixels and a background mask of 1 pixel, keeping 1 pixel as a buffer zone between the bounding box and background mask. For the intensity determination, the sum and mean of all the 4 pixels inside the bounding box mask was used. Traces were exported in JSON format and visualized using in-house software *Traceanalyser* (https://github.com/piedrro/TraceAnalyser).

To generate the scatter plots for the localization maps, we used the following procedure. Reference localizations were first identified from a master Picasso.hdf5 file and used as coordinates of interest for region-specific super-resolution reconstruction. For each selected reference index, the corresponding x–y coordinates were extracted and defined as the center of a spatial neighborhood. Raw single-molecule movies (.tif) were loaded using the one_channel_movie interface and localized using Picasso’s least-squares fitting routine (lq_fitting) with GPU acceleration (when available) and a fitting window of 5 pixels. Previously processed localization files (.hdf5) were loaded directly without re-fitting. All analyses were restricted to a predefined rectangular region of interest to reduce computational load and exclude irrelevant image areas. The x and y coordinates of the localizations were compiled into a two-dimensional array, and a KD-Tree spatial index was constructed to enable efficient spatial queries. For each reference coordinate, localizations within a radius of 2 pixels were identified using a radius-based neighbor search (query_ball_point). The matched localizations were extracted while preserving associated metadata (e.g., frame number, photon counts, and localization precision) and exported as independent HDF5 files for each region and movie. The resulting per-region localization datasets were subsequently rendered using Picasso’s visualization tools and OriginPro 2021 (OriginLab). For comparative analysis, localizations corresponding to different DNA sequences were plotted using distinct color channels to enable direct visual discrimination in the reconstructed super-resolution images.

For all dwell-time analysis, traces for each experiment were fitted using a 2-state Hidden Markov model (integrated in traceanalyser) for 1-base sequencing and an automatic state-selection model for 3-base and 5-base sequencing. Histograms and traces were plotted in OriginPro 2021 (OriginLab).

### Base calling

For base calling on each gapped-DNA molecule (identified in the green channel), we used in-house Python-based tools (https://github.com/Z-Qing/GapSeq-repo) to count the number of localizations due to the binding of labeled seal-DNA in the red channel. Localizations in the green channel were determined using Picasso with a gradient threshold of 400. Localizations in the red channel were found using a gradient threshold of 1000, with the following camera parameters: baseline = 400, box size = 5, EM gain = 1, quantum efficiency = 0.82, sensitivity = 2.5, and pixel size = 117 nm. We used the same parameters for all movies collected throughout all the experiments.

For each localization in the donor channel, the total number of all four seal localizations in the red-emission channel within a radius of 2 pixels was counted. To define a threshold number of localizations above which only we will call the bases (a “base-calling threshold”), we first counted all localizations from all the interrogation movies for one position and acquired the histogram of localization counts (LC) per molecule, and the probability density function (PDF) of the localization counts using Gaussian kernel density estimation (Supplementary Fig. 4A). The histogram of localization counts is typically bimodal or skewed, consisting of a low-count population corresponding to background or weak-binding (as for mismatch seal binding in the non-competition experiment) and of a higher-count population due to repeated specific binding of the complementary seal. As we move from low LC values to high LC values, we encounter a transition point (i.e., an LC value) where the frequency stops decreasing fast, marking the transition from weak to strong binding, and defining an LC threshold that can differentiate between mismatch seal binding and complementary seal binding.

To identify the transition point in a robust way, we first compute the first derivative of the LC histogram; the global minimum in the first derivative corresponds to the point of steepest decline between mismatch-dominated and complementary-dominated populations. We then fit the first derivative function with an exponential decay (Ae^-Bx^, where B is the decay-length parameter obtained from the exponential fit) from its global minimum (x_start_) onward. We then use x_start_ + 3*B as the final base-calling threshold LC value (Supplementary Fig. 4B). At least one LC value should be higher than this threshold for the non-competitive version of the assay; further, at least three LC values should be higher than this threshold for the competitive inhibition version of the assay.

Subsequently, we performed additional filtering to remove molecules irreversibly adsorbed to the surface near the gapped DNA, but not interacting with DNA; such molecules show persistent localization clustering over time (signifying non-specific binding of a seal to the surface, which can artificially inflate localization counts and bias base calling). To remove such molecules (that show at least 100 localizations), we first assigned localizations in consecutive frames to a “localization bundle”, representing a contiguous temporal binding event. The bundle thresholds were chosen based on the different gap lengths used for single- and multiple-base calling experiments. Specifically, we require at least five bundles for single-base calling, or two bundles for sequencing multiple bases; testing of higher bundle thresholds did not significantly improve base-calling accuracy, but instead substantially reduced the number of analyzable molecules. We also require that the longest bundle should be smaller that 50% of the total movie frames for single-base calling, or 80% of the total movie frames for sequencing multiple bases. If the longest bundle occupies more than this fraction of the total movie frames, it likely corresponds to a surface-adsorbed fluorophore (or labeled seal) rather than a transiently hybridized seal. When both conditions related to the number of bundles and the relative duration of the longest bundle are met, the molecules are accepted, otherwise rejected.

For every gap localization, we found the four localization counts for each of the seal-interrogations and took the difference between the largest and second-largest localization counts. Confidence of calling the base is defined as following:

1 $${{difference}}_{{non}-{comp}}={largest}\,{count}-{second}\,{largest}\,{count}$$

2 $${{confidence}}_{{non}-{comp}}=\frac{{difference}}{(95\,{percentile}\,{of}\,{difference})}$$

This logarithmic transformation enhances the dynamic range and stability of the confidence metric. Selecting the gap molecules with higher confidence increases the accuracy of sequencing (to ~98%) while still maintaining most molecules (Supplementary Fig. 4C).

In the competitive-inhibition approach, three out of four traces exhibit more prominent binding. Using a similar base-calling threshold, we chose the trace exhibiting the least number of localizations as the one complementary to the sequence at the corresponding gap position. To determine a confidence metric for base-calling, we used the difference between the second-lowest localization count and the lowest localization count values out of the four U-seal interrogations.

3 $${{difference}}_{{comp}}={second}\,{least}\,{count}-{least}\,{count}$$

4 $${{confidence}}_{{comp}}=\frac{{difference}}{(95\,{percentile}\,{of}\,{difference})}$$

### Data analysis for CAP-binding assay and in situ sequencing

All movies were undrifted and aligned, and traces were determined as described above. Traces were then fitted using an HMM model with automatic state-selection, and diagonal covariance fitting with two-state detection method; incorporated in the traceanalysis software and described previously^19–21^. All traces exhibiting binding were exported in CSV format. Base calling for the localized CAP-binding DNA molecules was done using the base-calling algorithm in the previous section. Bases are called above a confidence threshold value of 0.4.

Dwell times and identified bases were aligned using in-house MATLAB script. Dwell-time and unbound-time histograms were fitted using double-exponential and mono-exponential decay functions, respectively, in Origin (OriginPro2022). We then determined the rates of binding, unbinding, and the equilibrium binding constant using following equations:

5 $${k}_{{off}}=1/{mean}\,{dwell}\,{time}$$

6 $${k}_{{on}}=1/{{\mathrm{concentration}}}\,{{\mathrm{of}}}\,{{\mathrm{CAP}}}\times {{\mathrm{mean}}}\,{{\mathrm{unbound}}}\,{{\mathrm{time}}}$$

7 $${K}_{{{{\rm{d}}}}}=\frac{{k}_{{off}}}{{k}_{{on}}}$$

### Single-molecule FRET data analysis for transcription assay and in situ sequencing

All movies were undrifted and aligned, and traces were determined as described above. For all further traceanalysis and sequence calling, we used localizations in the green channel (which is also the donor channel for the smFRET measurements) collected immediately after the immobilization of the promoter library. All sequencing movies were aligned using the fiduciary marker positions in the green channel during the transcription assay movie. Using a 4-pixel mask and 1-pixel background mask, we determined intensity vs time trajectories of the intensity of donor emission upon donor excitation (*I*_DD_), the intensity of acceptor emission upon donor excitation (*I*_DA_), and the intensity of acceptor emission upon acceptor excitation (*I*_AA_). Traces obtained for transcription assay were further visualized and analyzed using “Traceanalysis” software. Intensity vs. time trajectories were curated to exclude multiple-step donor and acceptor photobleaching, and trajectories exhibiting donor and acceptor photoblinking before or during E_FRET_ reaching 0.4.

Apparent FRET efficiency (E_FRET_) was determined using the following formula

8 $${E}_{{FRET}}={I}_{{DA}}/\left({I}_{{DA}}+{I}_{{DD}}\right)$$

Intensity time-traces exhibited transitions between various distinct E_FRET_ states possessing anticorrelated *I*_DD_ and *I*_DA_ changes. Single-molecule FRET trajectories were idealized using the hidden Markov model (HMM) hmmlearn (as implemented in Traceanalysis) with diagonal-covariance Gaussian emissions. A single HMM was trained on all trajectories, with shared emission and transition parameters and learned from the full dataset. As such, while emission means were allowed to vary during training, they were not fitted independently for each trajectory. Instead, they were optimized globally, resulting in a single set of emission means common to all molecules. Emission means were initialized near the peaks of the empirical FRET efficiency distribution (Fig. 22D), and transition probabilities were initialized uniformly. To sample all main FRET states from open complex to an elongation complex (which also includes the main states occupied by cycling or paused molecules), we combined all traces corresponding to escape events (i.e., the ones showing transitions that sampled the E_FRET_ ~0.6 state) and plotted the E_FRET_ histogram prior to donor or acceptor bleaching; we then fitted the distribution using a sum of Gaussian distributions. Since the E_FRET_ values corresponding to different initial-transcription stages were known from our previous work (21), we used them as initial values for fitting. Since emission means are defined globally, individual E_FRET_ values falling between idealized state levels were assigned to the nearest state based on the emission distributions.

To ensure robustness, the fitting procedure was repeated with multiple random initializations (100 restarts), and the solution with the highest likelihood was selected. We kept a minimum state length of two frames or 0.4 s. The state number was chosen based on biologically interpretable FRET levels and validated for robustness, since automatic state-selection methods frequently overfit noise in single-molecule FRET trajectories. We fixed the number of states to four based on known E_FRET_ levels observed in the data (E_FRET_ ~0.22, 0.38, 0.51, and 0.63).

Verification of the four-state model has been carried out using the metric of log-likelihood (logL), change in log-likelihood (∆L), Akaike Information Criterion and Bayesian Information Criterion (BIC). LogL measures how well a given model explains the observed FRET data, and is computed as the logarithm of the probability of observing the data under the fitted model; higher log-likelihood indicates a better fit to the data. On the other hand, ∆L quantifies the improvement in model fit when increasing the number of states: a large and positive ∆L implies that the addition of a state significantly improves the model, a ∆L ~0 means little or no improvement, and a negative ∆L indicates overfitting. AIC and BIC quantify the trade-off between goodness-of-fit and model complexity. Both criteria are defined as a function of the log-likelihood and include a penalty term that increases with the number of model parameters (i.e., the number of states).

9 $${AIC}=-2log L+2p$$

10 $${BIC}=-2{log}L+p{log}\left(N\right)$$

where *p* is the number of independent parameters, and *N* is the number of observations. Following HMM idealization, individual trajectories were classified manually based on quantitative features extracted from the idealized state sequence. Overall, we found four distinct classes of traces. Class I molecules give traces where E_FRET_ starts from ~0.22 and reaches ~0.63. Class II molecules give traces where E_FRET_ cycles between ~0.22 and ~0.38 values; these molecules can have high or low frequency cycling, but we didn’t further separate them into different subclasses; we required at least two cycles during the trace in order to place a molecule in this Class. Class III molecules give traces where E_FRET_ shows a transition from ~0.22 to ~0.38 and then returns to ~0.22 without exhibiting further transitions. Class IV molecules give traces that exhibit a transition from ~0.2 to ~0.38 without returning to ~0.2 or showing any other transition during the time of data acquisition (or before photobleaching). For the functional assignment of these classes, see the main text. A few trajectories (17 traces, ~0.8% of all transcriptionally active molecules) exhibited paused E_FRET_ values in the 0.45–0.50 range without pausing at the usual ~0.38 state. These traces were classified as “ambiguous”. Given their low abundance and lack of clear functional state assignment, these traces were excluded from further analysis; inclusion of these traces did not affect the overall distribution of FRET states or any of the conclusions.

Base-calling was performed using the competition-assay base-calling algorithm described earlier, and sequences were linked to FRET time-traces. We excluded FRET time-traces for which the base-calling was not possible due to the localization count being below the base-calling threshold, or due to U-seal interrogations exhibiting a similar localization count for all four.

Time-traces for FRET and sequencing interrogations were aggregated from 24 separate movies (collected from independent experiments), which gave 1948 transcriptionally active molecules. Out of the 1710 transcriptionally active molecules that gave a usable sequencing read, 1447 were used for the pause-time analysis, and 263 were stably scrunched.

For each time-trace with an assigned sequence, the pause time was determined using the dwell-time at E_FRET_ ~0.4 (corresponding to the paused complex at ~6-nt RNA) for Classes I, II and III. For Class II molecules, we considered only the first E_FRET_ ~0.4 dwell. Pause-time histograms (Fig. 8A and Supplementary Fig. 24) were plotted, binned, and fitted using mono-exponential decays in Origin2021. Propensities for each class of molecules for different sequences were determined by dividing the molecules falling into each class by the total number of transcribing sequenced molecules for that sequence. Class-propensity heatmaps (Fig. 8B–E) were plotted in Origin2021.

### Reporting summary

Further information on research design is available in the Nature Portfolio Reporting Summary linked to this article.

## Supplementary information

Supplementary Information

Reporting Summary

Transparent Peer Review file

## Source data

Source Data

## Data Availability

All data supporting the conclusions of this study are included in the main figures, Supplementary Figs., and associated Source Data files. Raw movies supporting the proof-of-principle experiments for the sequencing technique, together with representative data for transcription sequencing, have been deposited in the Figshare repository at https://doi.org/10.6084/m9.figshare.31963782. All raw data underlying the main figures are provided in the Source Data file. Owing to their large file size, additional raw movies relating to the transcription and sequencing experiments are available from the corresponding authors upon request. Requests should be directed to Achillefs N. Kapanidis (kapanidis@physics.ox.ac.uk) or Jagadish Hazra (jagadish.hazra@physics.ox.ac.uk), and use of data would be governed by policies mentioned in Oxford Research Archive for data (https://libguides.bodleian.ox.ac.uk/ld.php?content_id=35709477). Responses will normally be provided within 4 weeks. Source data are provided with this paper.

## Code availability

All custom-developed codes used for this study have been deposited in GitHub. Movie processing and trace determination have been carried out using molseeq (https://github.com/piedrro/napari-molseeq); trace visualization and state fitting have been performed using traceanalyser (https://github.com/piedrro/TraceAnalyser); localization counting and base calling have been performed using a custom-developed Python script (https://github.com/Z-Qing/GapSeq-repo). All source code is available at https://doi.org/10.5281/zenodo.20848891.

Any additional requests for materials and datasets should be addressed to A.N.K. (kapanidis@physics.ox.ac.uk).
