# Supplementary material for: Unraveling single-molecule reactions via multiplexed in-situ DNA sequencing
Source: Nat Commun. 2026 Aug 29;17:10639. doi: 10.1038/s41467-026-77308-3 (PMC13649778; doi:10.1038/s41467-026-77308-3)
Supplement: Supplementary file 1 [file 41467_2026_77308_MOESM1_ESM.pdf]

# Supplementary Information for

## Unraveling single-molecule reactions via multiplexed in-situ sequencing

Jagadish Prasad Hazra, Rebecca Andrews, Piers Turner, Qing Zhao, Horst Steuer, Afaf H. El-Sagheer, Abhishek Mazumder, Hafez El Sayyed, Mirjam Kümmerlin, Tom Brown, Achillefs N. Kapanidis

Corresponding authors: kapanidis@physics.ox.ac.uk; jagadish.hazra@physics.ox.ac.uk

**The PDF file includes:**

Supplementary Figures 1 to 26
Supplementary Tables 1 to 4
Supplementary References

## Supplementary Figures

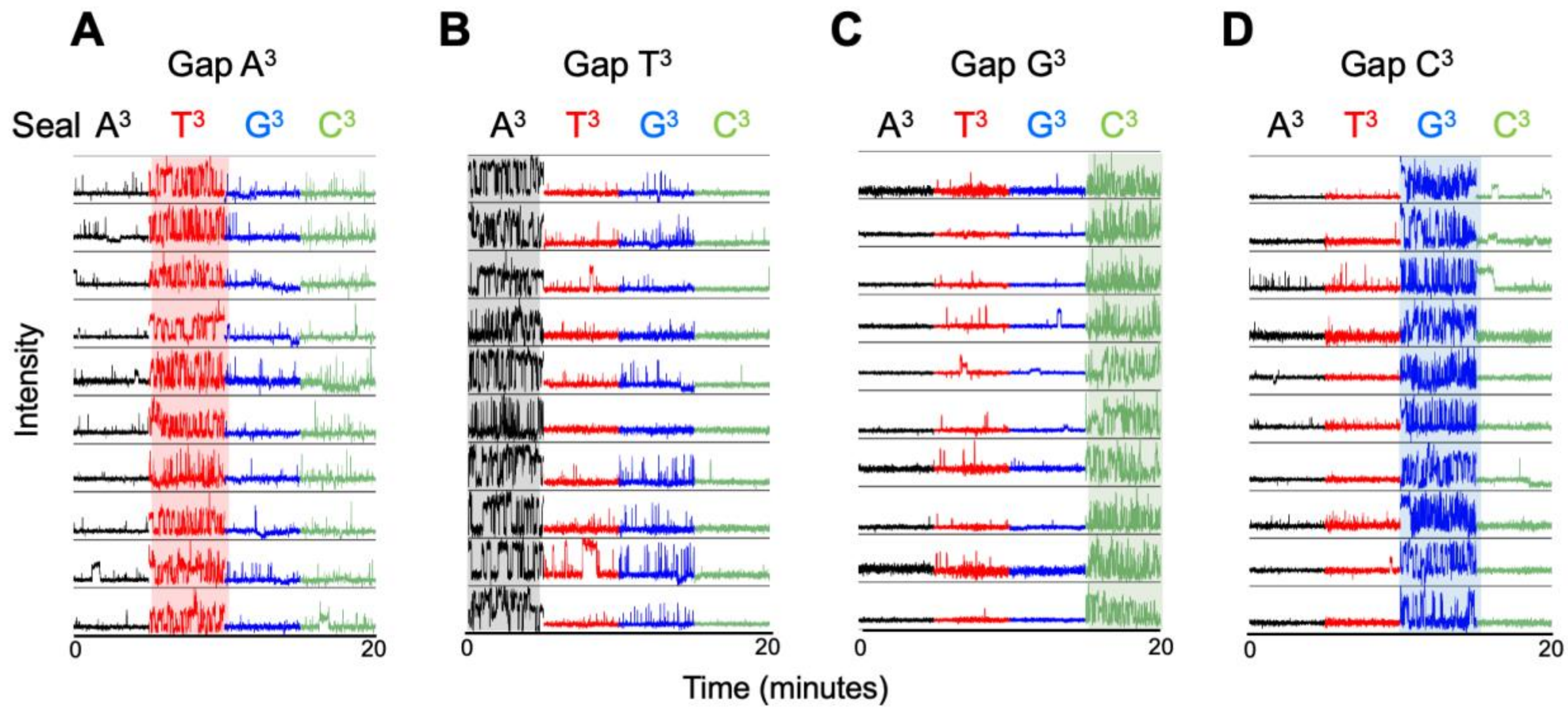


**Supplementary Figure 1. Additional example traces for identification of single nucleotide differences using transient DNA hybridization.**

**A**. An 8-nt DNA gap is sequentially interrogated at gap position 3 (where the base is an A) using 4 seals that differ only on the base opposed to the interrogated position. Only the seal featuring A at position 3 (red traces) shows strong gap binding; the remaining seals show negligible binding.

**B-C.** Analysis as in panel A, but for gaps featuring a T, G, and C at gap position 3.

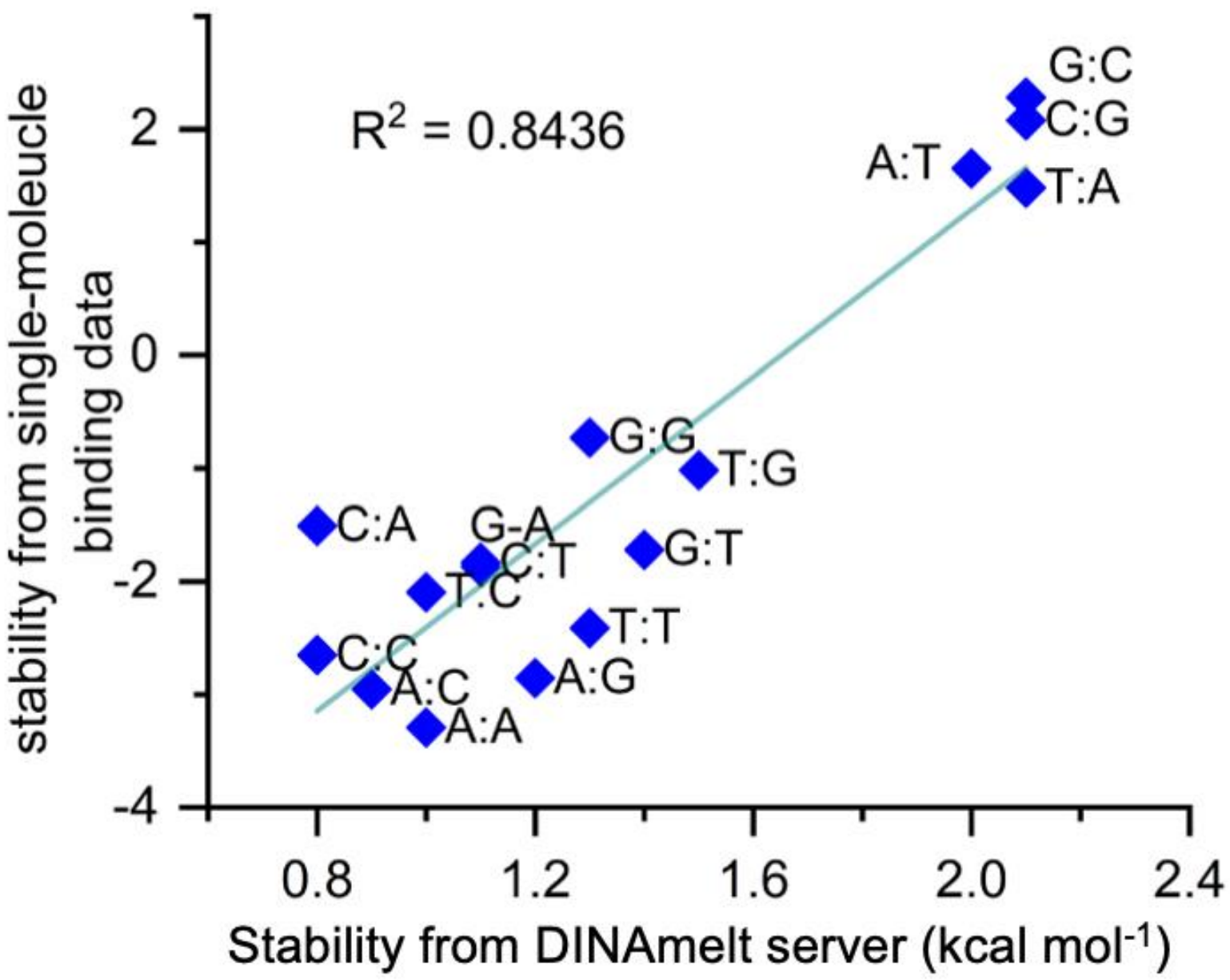


**Supplementary Figure 2. Comparison of thermodynamic stability of transient gap-seal duplexes as measured at the single-molecule level with prediction from computational approaches.**

Comparison of stabilities obtained from single-molecule analysis with stability estimates the DINAmelt server *(17)* using the context used in our experiments. An additional base-pair was added to the 8-mer to emulate the effect of stacking provided by the two ends of the gap. The results are highly correlated ($R^2 = 0.844$).

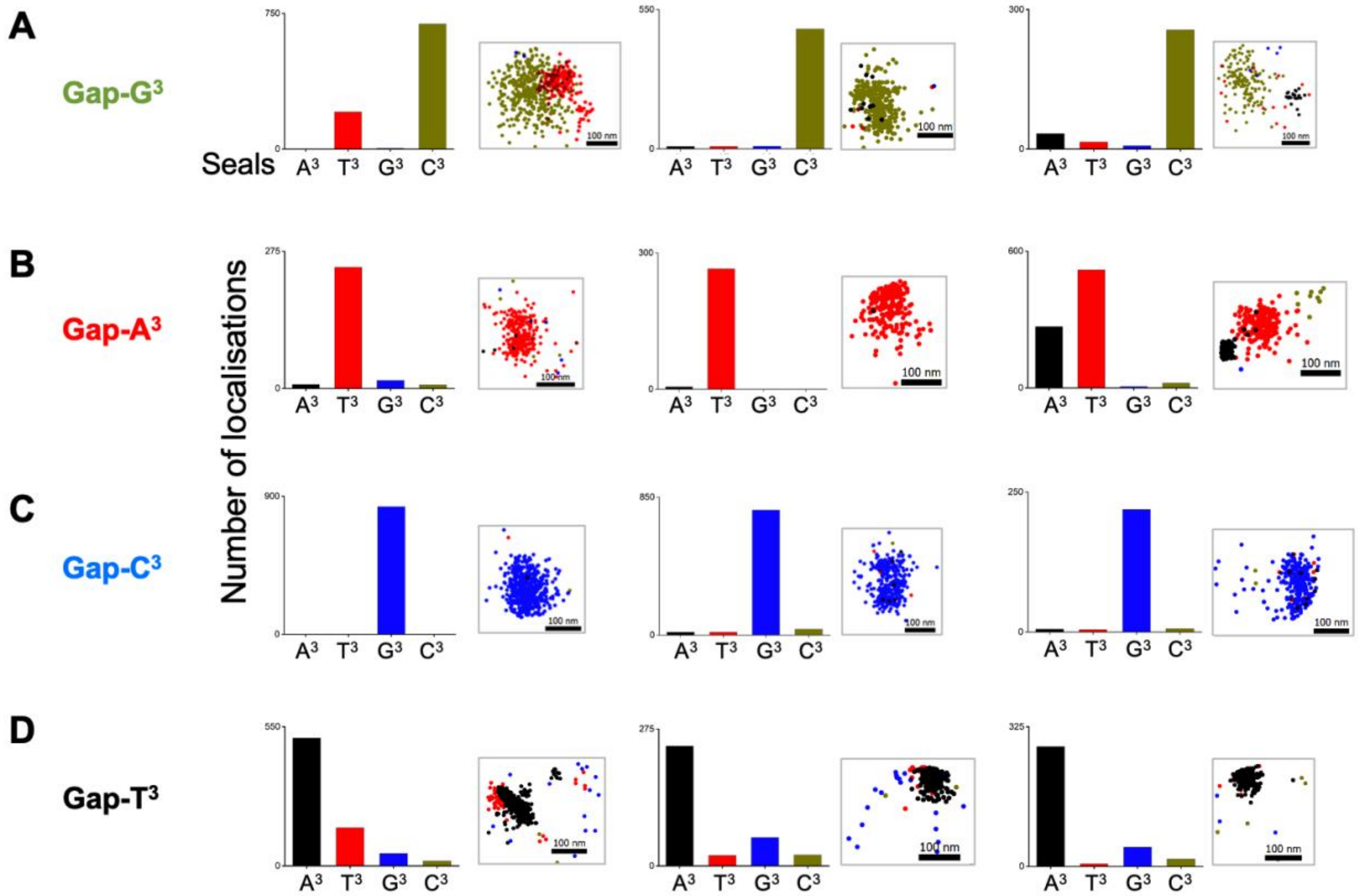


**Supplementary Figure 3. Quantification of seal binding using localization counting.** Representative plots for single-molecule localization counts and locations for seal binding to gap molecules featuring a different base at gap position 3 (Gap-$G^3$, Gap-$A^3$, Gap-$C^3$, and Gap-$T^3$).

**A.** Bar plots: single-molecule localisation count for three example Gap-$G^3$ molecules sequentially interrogated with 4 labelled seals; in all three cases, we observe many more localizations for the complementary seal (seal $C^3$). Scatter plots: maps of seal-binding localizations; localisations due to the complementary seal are clustered in near-circular pattern; occasionally, mismatch seals bind to a proximal (but different) location (e.g., in the first example, seal $T^3$ binds ~100 nm away from the main cluster), leading to background; these results point to the possibility of using spatial filtering to improve base-calling accuracy.

**B-D.** As in A, but for gap molecules Gap-$A^3$, Gap-$C^3$, and Gap-$T^3$.

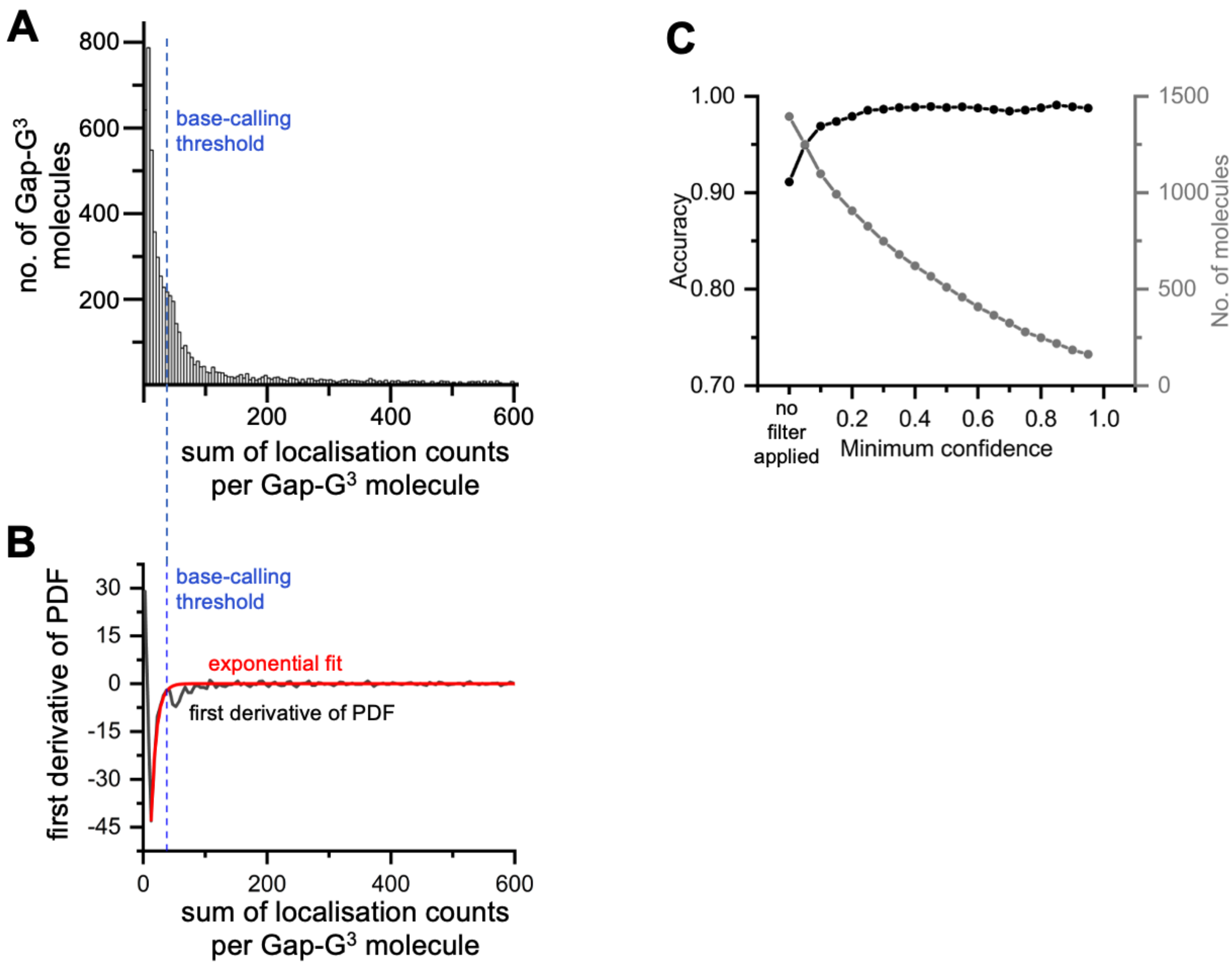


**Supplementary Figure 4. Base-calling method and accuracy test.**

**A.** Frequency histogram of all localization counts due to the four seals binding to Gap-$G^3$. Total number of events in the histogram N = 1640.

**B.** First derivative of the probability density function of the frequency of localisation counts (black line) and fit to an exponential decay function (red line). Blue line, threshold value of localization count; the threshold is defined as the localisation count of first derivative minimum (in this example, 13 localisations) plus three times the decay length (in this example, 8 localisations) obtained from fitting. So, the final base-calling threshold in this example is set to 37 localisations. After filtering, we keep only molecules for which at least one localisation count (out of four interrogations) is higher than the threshold.

**C.** Accuracy and number of correctly called molecules with increasing confidence value (see SI text for the definition of confidence values).

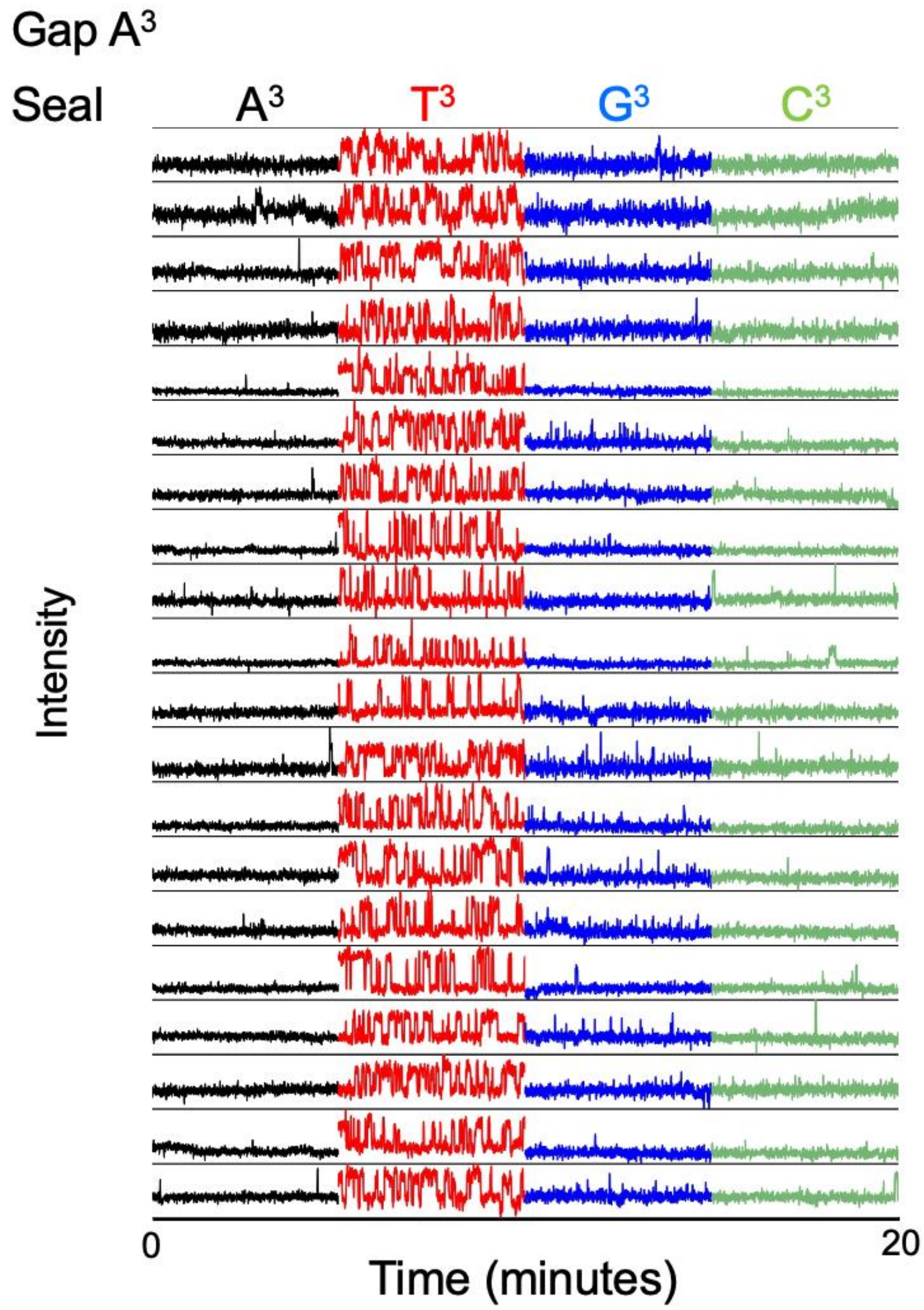


**Supplementary Figure 5. Sequencing an 8-nt gap-DNA containing a stretch of 8 A bases.**

**A.** Representative traces obtained from four sequential interrogations using four seals possessing A, T, G and C at gap position 3, and all T bases at all other positions. We observed many more binding-unbinding cycles for seal $T^3$ (complementary seal) compared to the other three 1-nt mismatched seals.

**B.** We performed localization counting and base calling for all gapped DNA molecules, showing an ~97.3% accuracy at a confidence of 0.3, calling ~850 molecules.

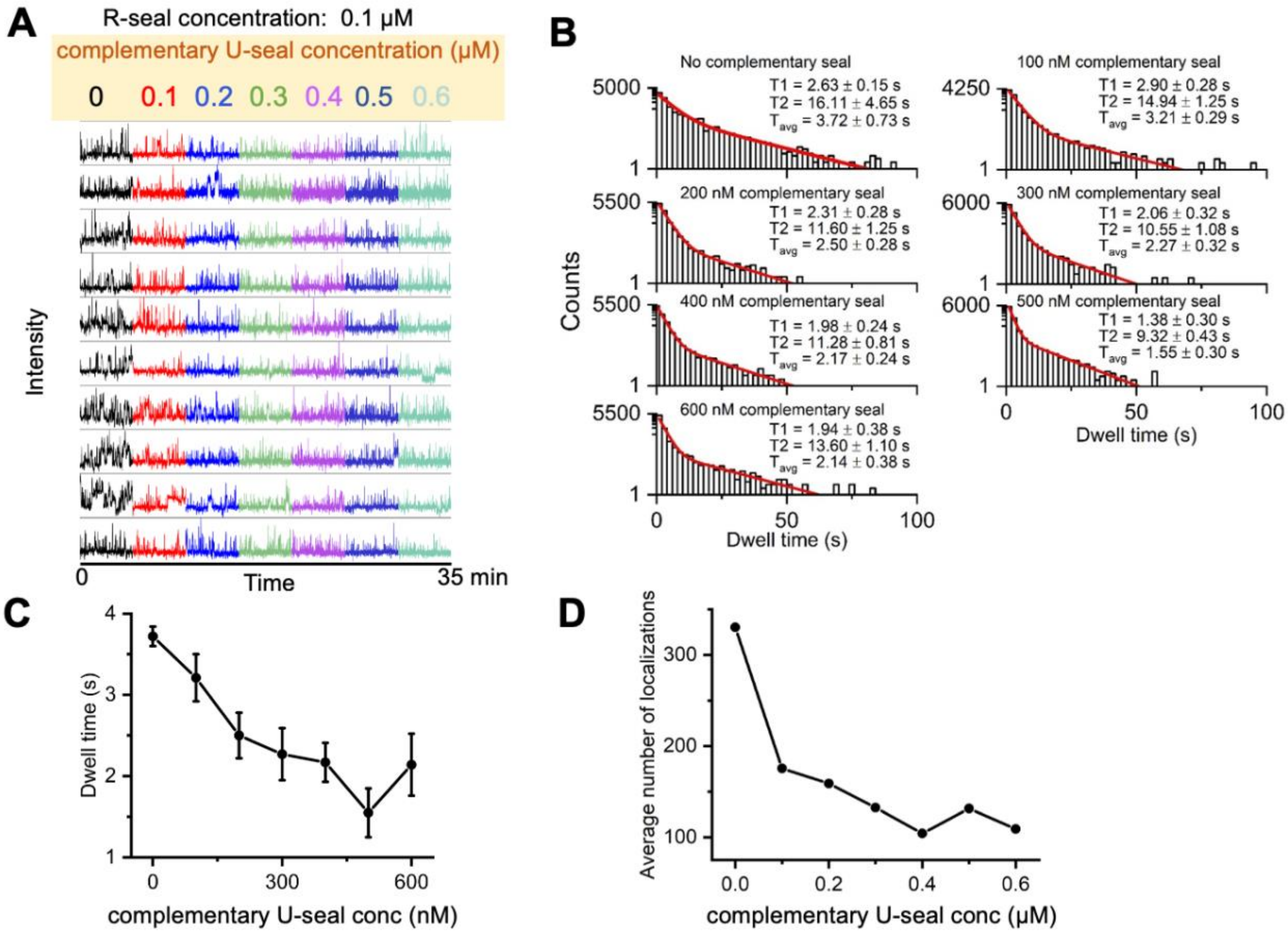


**Supplementary Figure 6. Quantifying the effect of competitive inhibition of R-seal binding to a gap due to increasing concentrations of a complementary U-seal.**

**A.** Representative traces for gap-binding of 100 nM of R-seal R8-$N^3$ in presence of 100-600 nM complementary U-seal in IB2 buffer. Number of molecules N = 380.

**B.** Dwell time distributions for the different U-seal concentration. Histograms are fitted with double-exponential decay model (red lines).

**C-D.** Mean dwell-times (panel C) and localisation count (panel D) as a function of U-seal concentration.

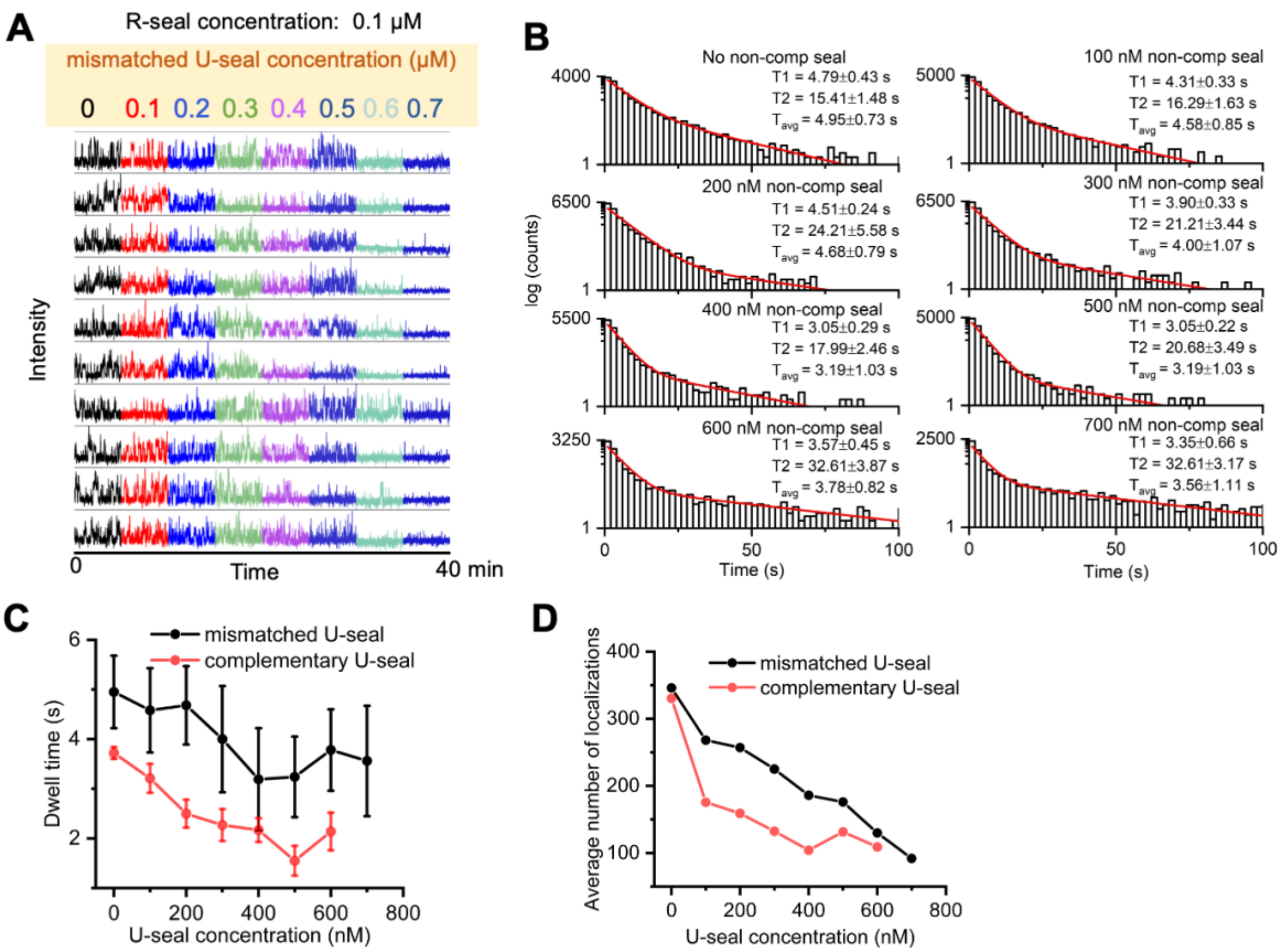


**Supplementary Figure 7. Quantifying the effect of competitive inhibition of R-seal binding to a gap due to increasing concentrations of a 1-nt mismatched U-seal.**

**A.** Representative traces for gap-binding of 100 nM of R-seal R8-$N^3$ in presence of 100-600 nM 1-nt mismatched U-seal (seal U8-$T^3$) in IB2 buffer. Number of molecules N = 664.

**B.** Dwell time distributions for the different U-seal concentration. Histograms are fitted with double-exponential decay model (red lines).

**C-D.** Mean dwell-times (panel C) and mean localisation count (panel D) as a function of U-seal concentration for mismatched seal (black) and complementary seal (red). For the 8-nt gap, increasing U-seal concentration reduces both the dwell time and the number of R-seal localizations, reflecting competitive binding. The effect is stronger for the complementary U-seal. At ~300 nM, this difference is maximized: the complementary seal strongly inhibits binding, whereas the mismatched seal permits binding. At >300 nM, the mismatched U-seal starts to inhibit binding, diminishing contrast. Decreases in the dwell times of the bound R-seal are comparatively similar, indicating that discrimination arises primarily from differences in on-rate. Based on the results of Supplementary Figures 6 and 7, we chose to use 100-200 nM R-seal and 300-600 nM U-seal (i.e., a 3:1 concentration ratio) for the competitive-inhibition assays.

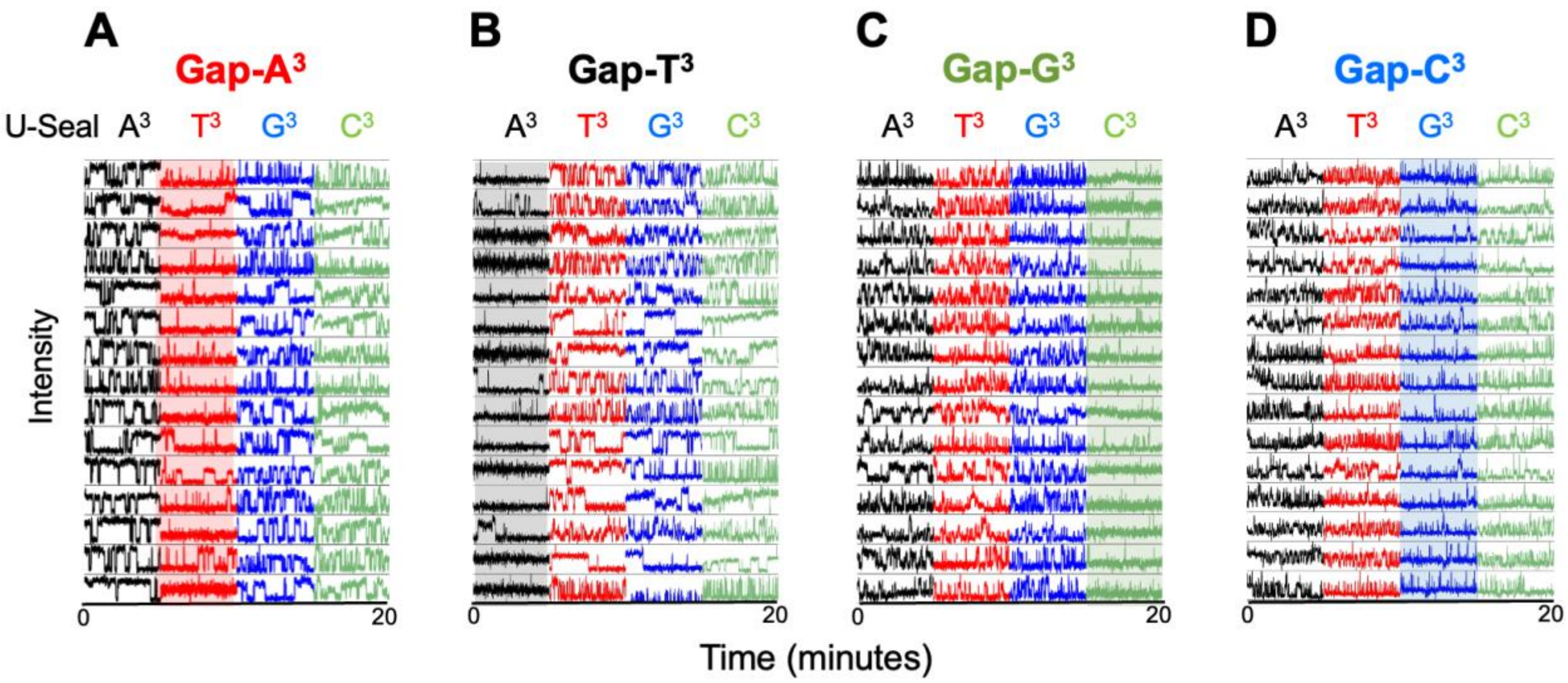


**Supplementary Figure 8. Reading single nucleotides using competitive inhibition: additional examples**.

**A.** Examining gap position 3 in Gap-$A^3$ using 200 nM of labelled R-seal and a set of 4 unlabelled U-seals (added sequentially, all at 600 nM); the complementary U-seal (U8-$T^3$, in red) suppress R-seal binding, enabling base identification.

**B-D**. Same analysis, but for gapped DNA molecules Gap-$T^3$, Gap-$G^3$, and Gap-$C^3$. Complementary U-seals lower the extent of R-seal binding (see highlighted columns of timetraces), enabling base identification.

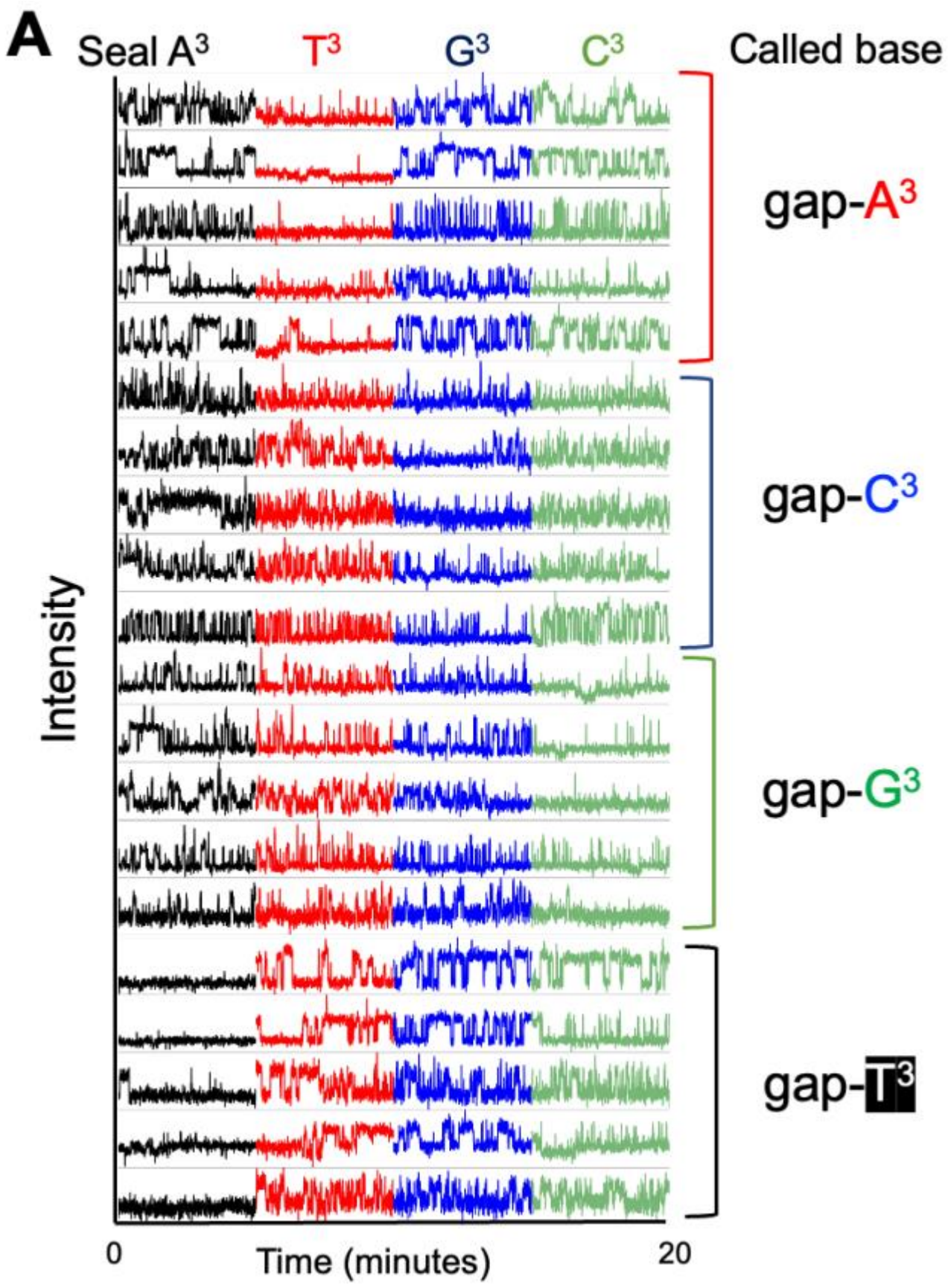


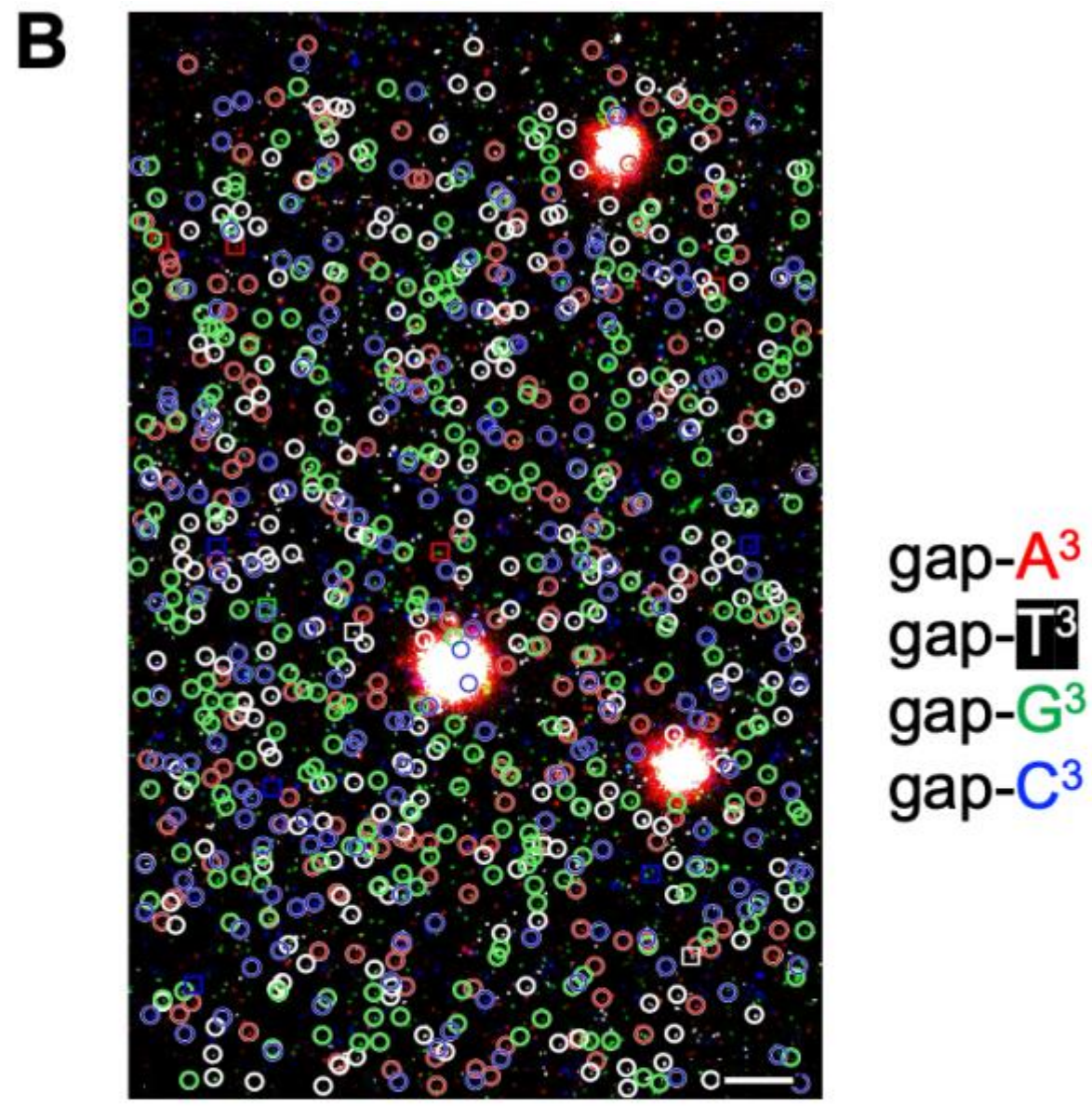


C

| Gap Base | Accuracy (%) |
|---|---|
| $A^3$ | 97.9% (184/188) |
| $T^3$ | 98.8% (243/246) |
| $G^3$ | 99.6% (224/225) |
| $C^3$ | 97.0% (190/196) |
| All bases | 98.4% (841/855) |

**Supplementary Figure 9. Validation of the competitive-inhibition approach using a mixed population of sequences with prior information.** All four gaps were deposited sequentially (as in **Fig. 3A**), followed by registration of their location on the surface, and photobleaching. DNA gaps were then interrogated using competitive inhibition using 200 nM of 8 nt R-seal and four cycles of interrogation with competing U-seals, each at 600 nM.

**A.** Sequential interrogation of the deposited DNA by seals specific for gap position 3. The absence of binding identifies the base in the interrogated position.

**B.** Field of view (50x80 µm) of molecules sequenced for gap position 3. White spots: fiducial markers. Circled molecules were identified correctly; in-square molecules were assigned incorrectly. The base-calling accuracy was ~98.4%. Scale bar: 5 µm.

**C.** Base-calling accuracies for individual bases.

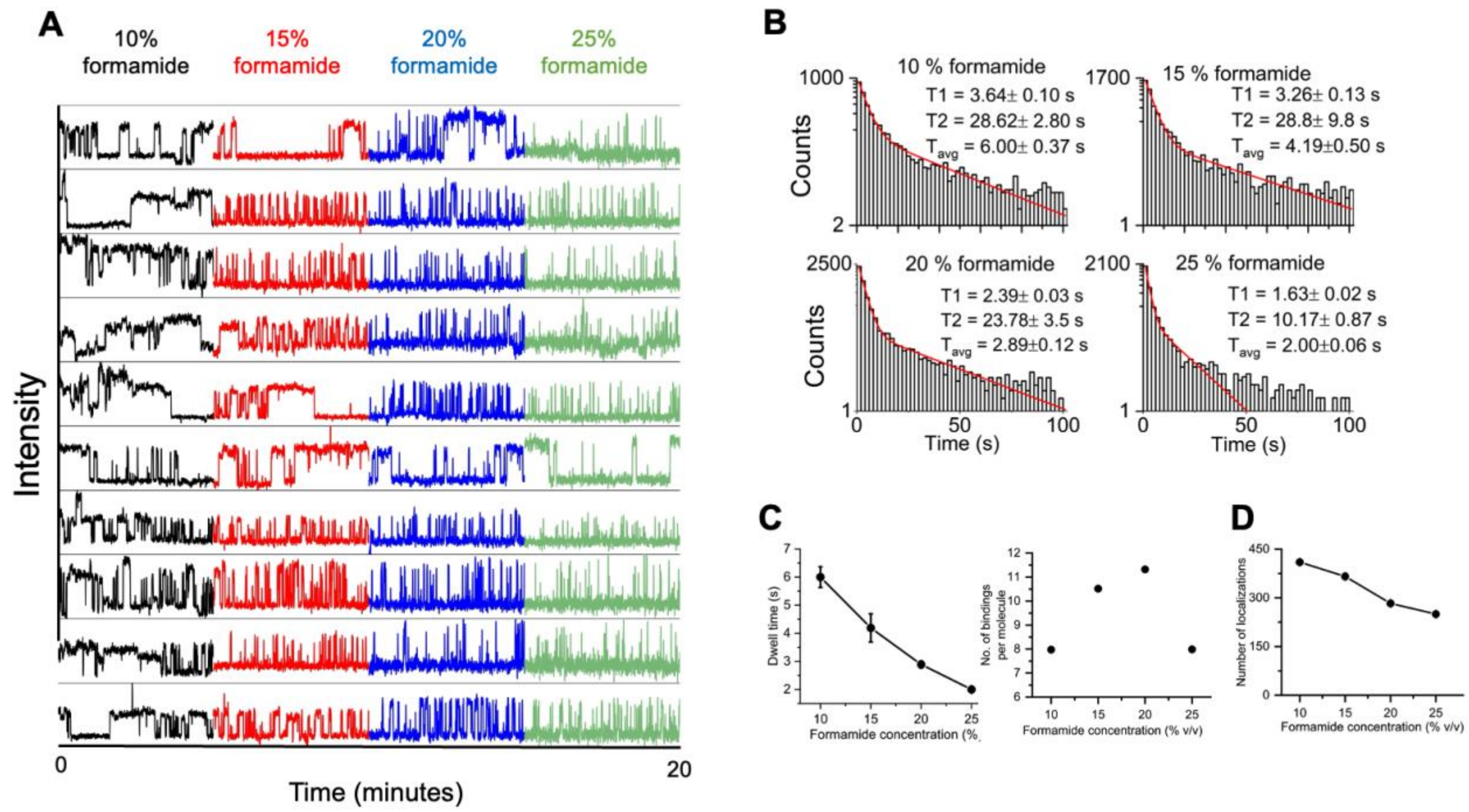


**Supplementary Figure 10. Optimizing formamide concentration for 13-nt seal binding of a gap.**

**A.** Representative traces from hybridization of 500 nM R-seal in IB3 (which contains 10% formamide) and in the presence of 10-25% formamide. Increasing formamide shortens dwell times in the bound (high-intensity) state. Number of molecules N = 422.

**B.** Dwell-time histograms for the bound states at different formamide concentrations. Histograms are fitted with double-exponential decay model (red lines).

**C.** Mean dwell times in the bound states (left) and number of binding-unbinding cycles per gap molecule (right) as a function of formamide concentration. Formamide concentrations of 15% and 20% provide an optimal combination of mean dwell time and number of binding-unbinding cycles.

**D.** Mean localisation count as a function of formamide concentration.

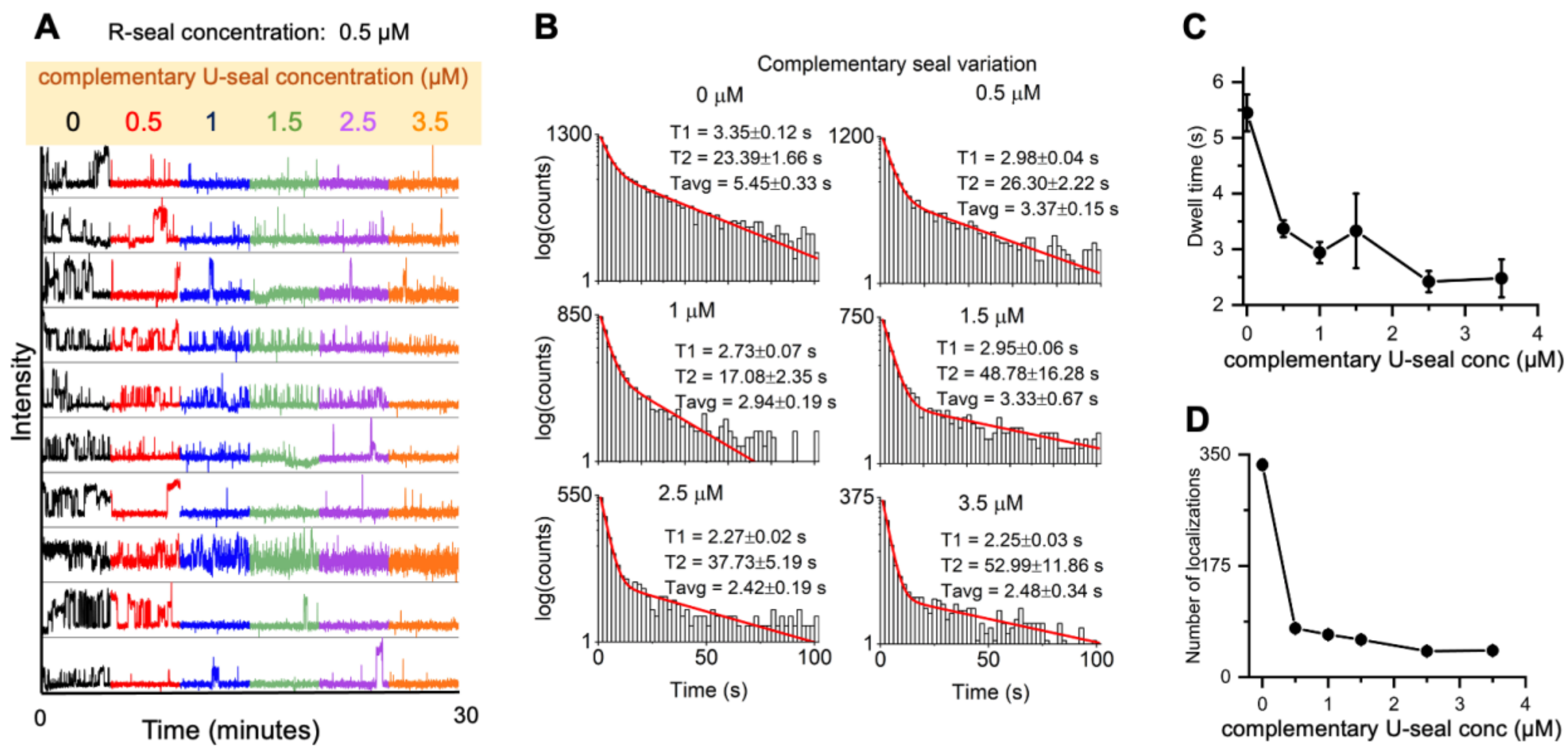


**Supplementary Figure 11. Quantifying competitive inhibition of R-seal binding to a gap due to increasing concentrations of a complementary U-seal: 3-base sequencing.**

**A.** Representative traces for gap-binding of 0.5 µM of R-seal R13-$N^5N^6N^7$ in presence of 0.5-3.5 µM complementary U-seal (U13-$C^5N^6N^7$). Number of molecules N = 642.

**B.** Dwell time distributions for the different U-seal concentrations. Histograms are fitted with double-exponential decay model (red lines).

**C-D.** Mean dwell-times (panel C) and mean localisation count (panel D) as a function of U-seal concentration.

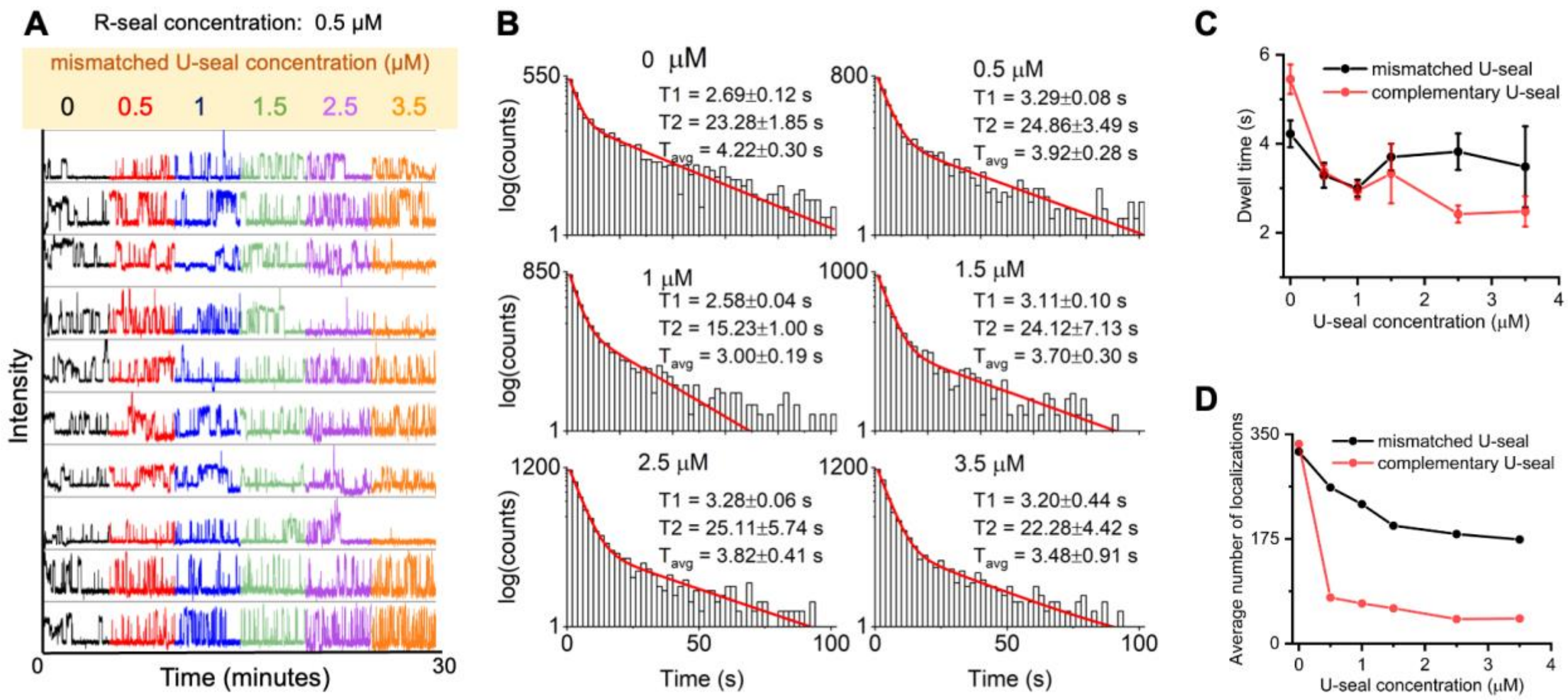


**Supplementary Figure 12. Quantifying the effect of competitive inhibition of R-seal binding to a gap due to increasing concentrations of a 1-nt mismatched U-seal: 3-base sequencing.**

**A.** Representative traces for gap-binding of 0.5 μM of R-seal R13-$N^5N^6N^7$ in presence of 0.5-3.5 μM 1-nt mismatched U-seal (seal U13-$T^5N^6N^7$ U13-$T^3$). Number of molecules N = 305.

**B.** Dwell time distributions for the different U-seal concentration. Histograms are fitted with double-exponential decay model (red lines).

**C-D.** Mean dwell-times (panel C) and localisation count (panel D) as a function of U-seal concentration for mismatched (black) and complementary seals (red). Dwell time decreases initially in the presence of both complementary or mismatched U-seals; the decrease is less pronounced at higher concentrations of the mismatched seal compared to the complementary U-seal. The average number of localizations corresponding to R-seal binding decreases sharply upon addition of complementary U-seal. At 1.5 μM U-seal concentration, the number of localizations decreases ~6-fold in the presence of the complementary U-seal, compared to only ~1.5-fold for the mismatched seal, providing clear contrast. Due to the relatively long gap (13 nt), binding of the degenerate R-seal is fairly stable once formed, resulting in comparable dwell times for both complementary and mismatched seals. Therefore, the main distinction between the two seals arises from differences in the on-rate. Based on the results in Figs S10-S12, we chose to use 500 nM R-seal and 1.5 μM U-seal in an assay buffer with 15% formamide for the competitive inhibition assay on 3-base sequencing, since they offer optimal contrast in R-seal binding between complementary and 1-nt mismatched U-seals.

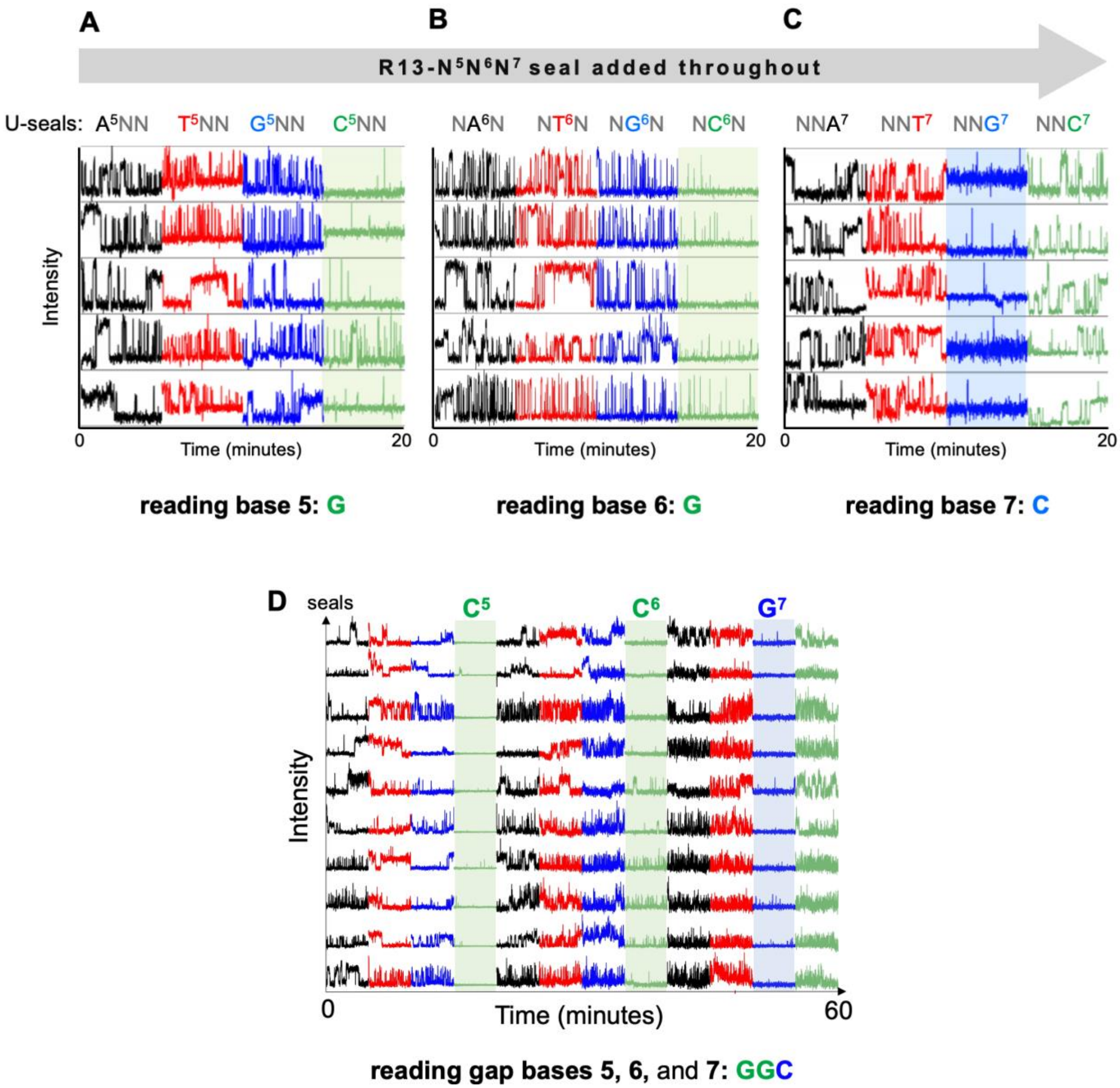


**Supplementary Figure 13. Reading three nucleotides using competitive inhibition**. **A-C.** Additional example traces for 3-base sequencing are provided for gap positions 5 (panel **A**), 6 (panel **B**), and 7 (panel **C**). Conditions and descriptions as in **Fig. 5B**. In all cases, the R-seal (R13-$N^5N^6N^7$) was kept at 500 nM, and each base was evaluated on a different surface. Interrogation with the complementary U-seal led to decrease binding (highlighted traces).

**D.** Sequential interrogation of the same sequence (GGC) on DNA molecules immobilized on the same surface. The overall accuracy for molecules that give reads for all three bases is ~99.5% (188/189), whereas the accuracy for the individual positions is 97.8% (393/402), 91.6% (318/347), and 96.7% (911/942) for bases 5, 6, and 7, respectively.

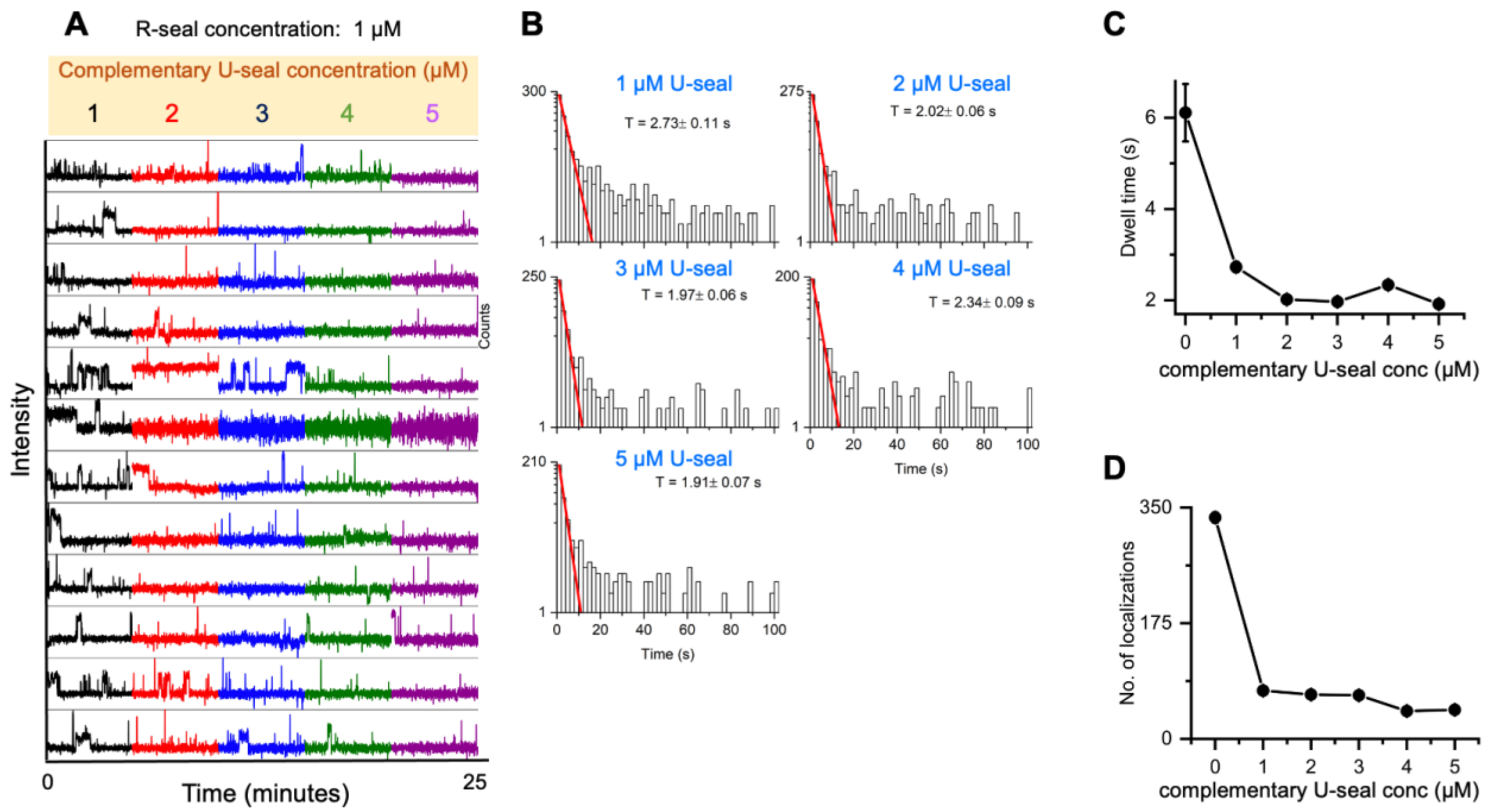


**Supplementary Figure 14. Quantifying competitive inhibition of R-seal binding to a gap due to increasing concentrations of a complementary U-seal: 5-base sequencing.**

**A.** Representative traces for gap-binding of 1 μM of R-seal R13-$N^5N^6N^7N^8N^9$ in presence of 1-5 μM complementary U-seal (U13-$C^5N^6N^7N^8N^9$). Number of molecules N = 180.

**B.** Dwell time distributions for the different U-seal concentrations. Histograms are fitted with mono-exponential decay model (red lines).

**C-D.** Mean dwell-times (panel C) and mean localisation count (panel D) as a function of U-seal concentration.

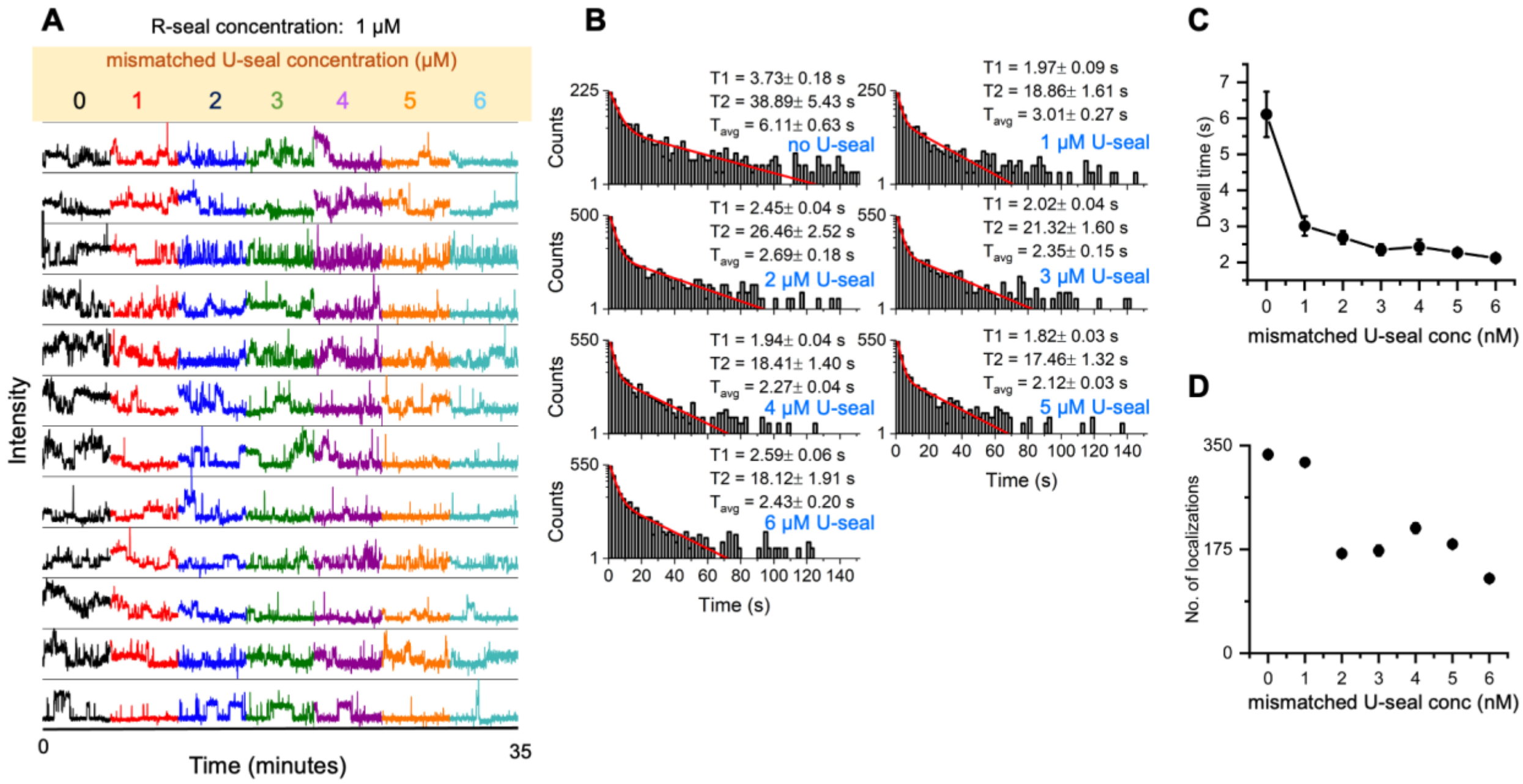


**Supplementary Figure 15. Quantifying the effect of competitive inhibition of R-seal binding to a gap due to increasing concentrations of a 1-nt mismatched U-seal: 5-base sequencing.**

**A.** Representative traces for gap-binding of 1 µM of R-seal R13-$N^5N^6N^7N^8N^9$ in presence of 1-5 µM complementary U-seal (U13-$T^5N^6N^7N^8N^9$). Number of molecules N = 205.

**B.** Dwell time distributions for the different U-seal concentration. Histograms are fitted with double-exponential decay model (red lines).

**C-D.** Mean dwell-times (panel C) and localisation count (panel D) as a function of U-seal concentration. Based on the results in **Figs S15-S16**, we chose to use 1 µM R-seal and 2 µM U-seal (a 1:2 concentration ratio) for the competitive inhibition assay for 5-base sequencing.

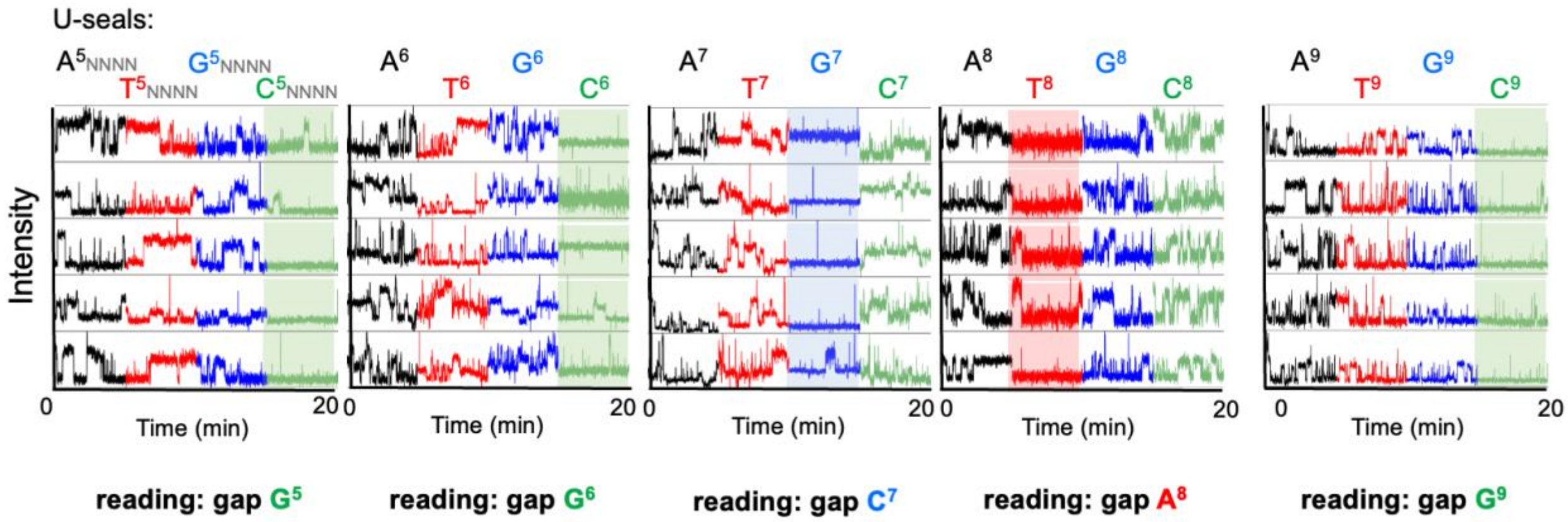


**Supplementary Figure 16. Reading five nucleotides using competitive inhibition: additional examples**. Traces for 5-base sequencing are provided for gap positions 5, 6, 7, 8 and 9. Conditions and descriptions as in **Fig. 5D**. In all cases, the R-seal (R13-$N^5N^6N^7N^8N^9$) and the U-seals were kept at 1 µM and 2 µM, respectively. Interrogation with the complementary U-seal led to decreased binding (highlighted traces).

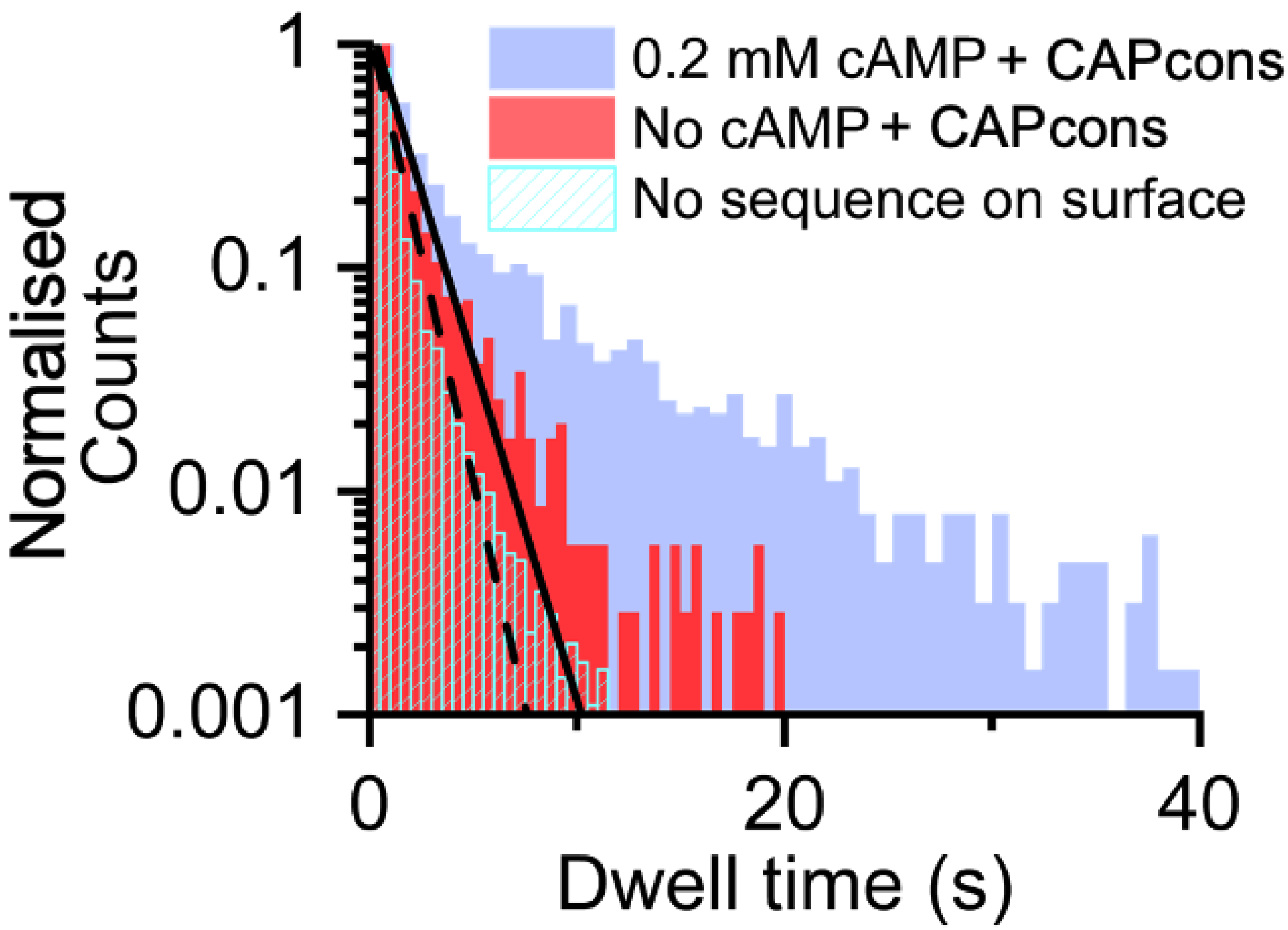


**Supplementary Figure 17. Control experiments for specificity of CAP-DNA interactions.** Dwell-time distributions for CAP binding to surface-immobilised molecules in the presence of CAPcons DNA and cAMP (grey columns); in the presence of CAPcons DNA only (no cAMP; pink columns; mean dwell time of 1.47 s; number of molecules N=823); and in the absence of CAPcons DNA and cAMP (cyan hatched bars; mean dwell time of 1.07 s). The comparison of the controls with the sample displaying specific binding supports that assignment of the long lifetime of ~9 sec to specific, cAMP-dependent binding of CAP to its consensus site.

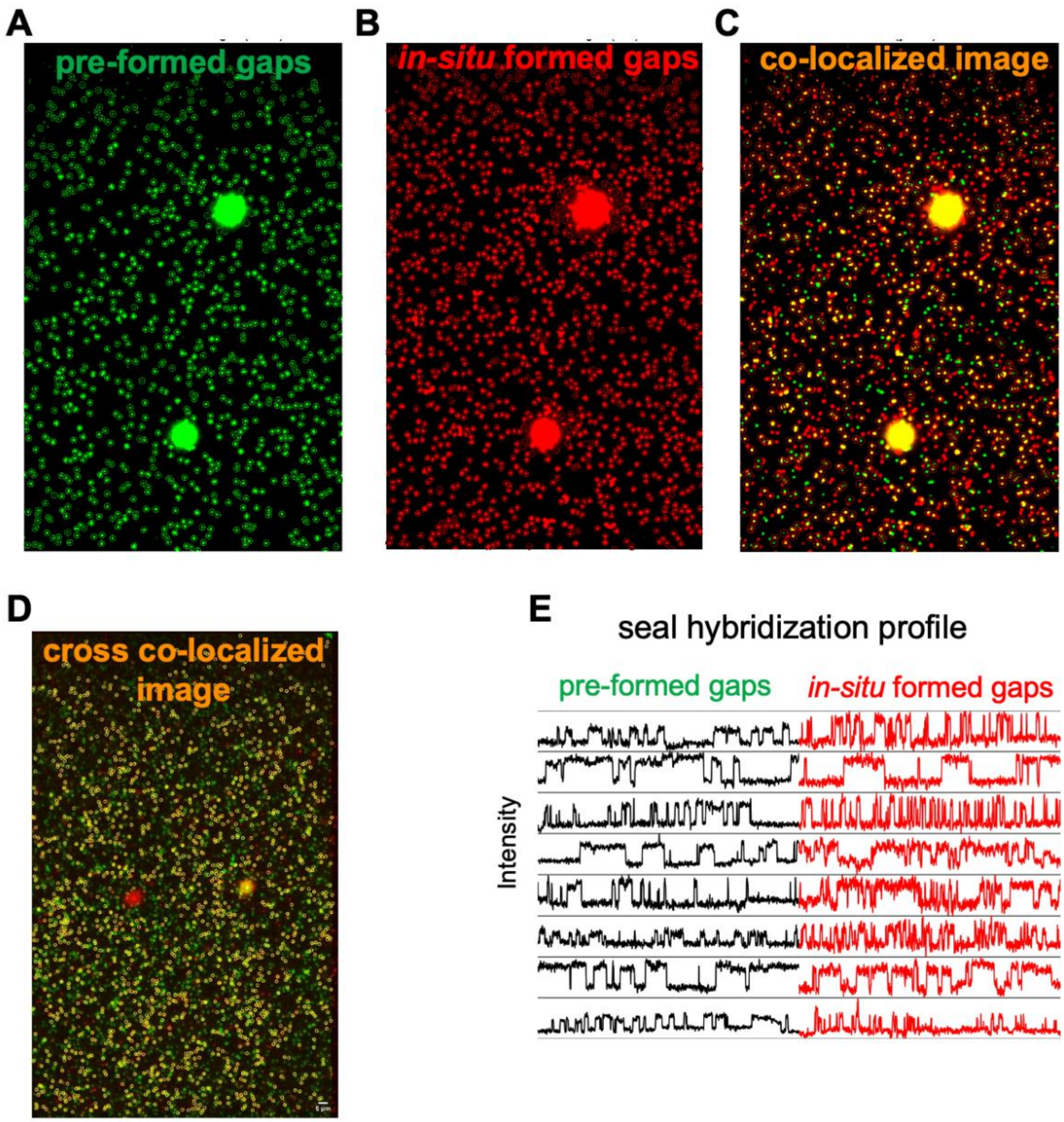


**Supplementary Figure 18. Efficiency and functionality of in-situ DNA gap formation.**
**A.** Localisation image of surface-immobilised 8-nt gapped DNA molecules.
**B.** Localisation image of *in-situ* formed surface-immobilised 8-nt gapped DNA molecules (same field-of-view).
**C.** Superimposed images of panels A and B; colocalised molecules appear yellow.
**D.** Cross-colocalization of flanking strands; cross-colocalised pairs of flanking strands on a single gap molecule appear yellow. For details, see SI text.
**E.** In black: time-traces of transient seal hybridization to pre-formed surface-immobilised gapped DNA. In red: time-traces of transient seal hybridization to the *in-situ* formed gapped DNA. The binding patterns pre-formed and in-situ formed DNA gaps are similar.

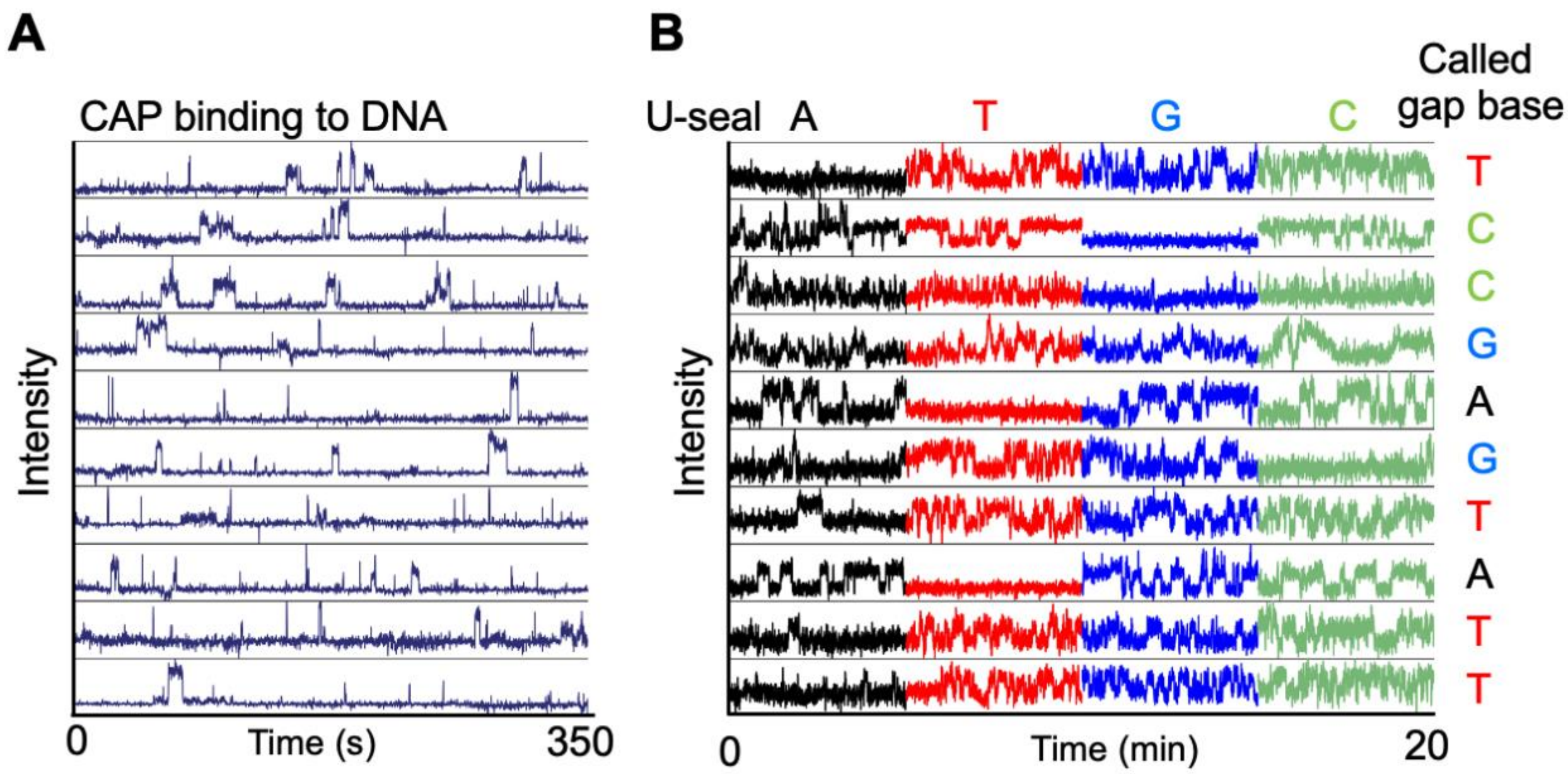


**Supplementary Figure 19. Representative traces for CAP-DNA interactions of the library followed by sequencing.**

**A.** Time-traces of CAP binding to members of the library of 4 DNA variants for position 5 within the CAPcons sequence. The assay employed 2.5 nM of Alexa647-labelled CAP.

**B.** Traces obtained from interrogation of the same DNA molecule as shown in the panel A using competitive inhibition of gap binding. The assay used 200 nM of R seal R9-CAP-$N^5$-Cy5 and 600 nM of 4 competing U-seals (black, red, blue and green for seals featuring A, T, G or C bases sequences, respectively, at the corresponding sequencing position).

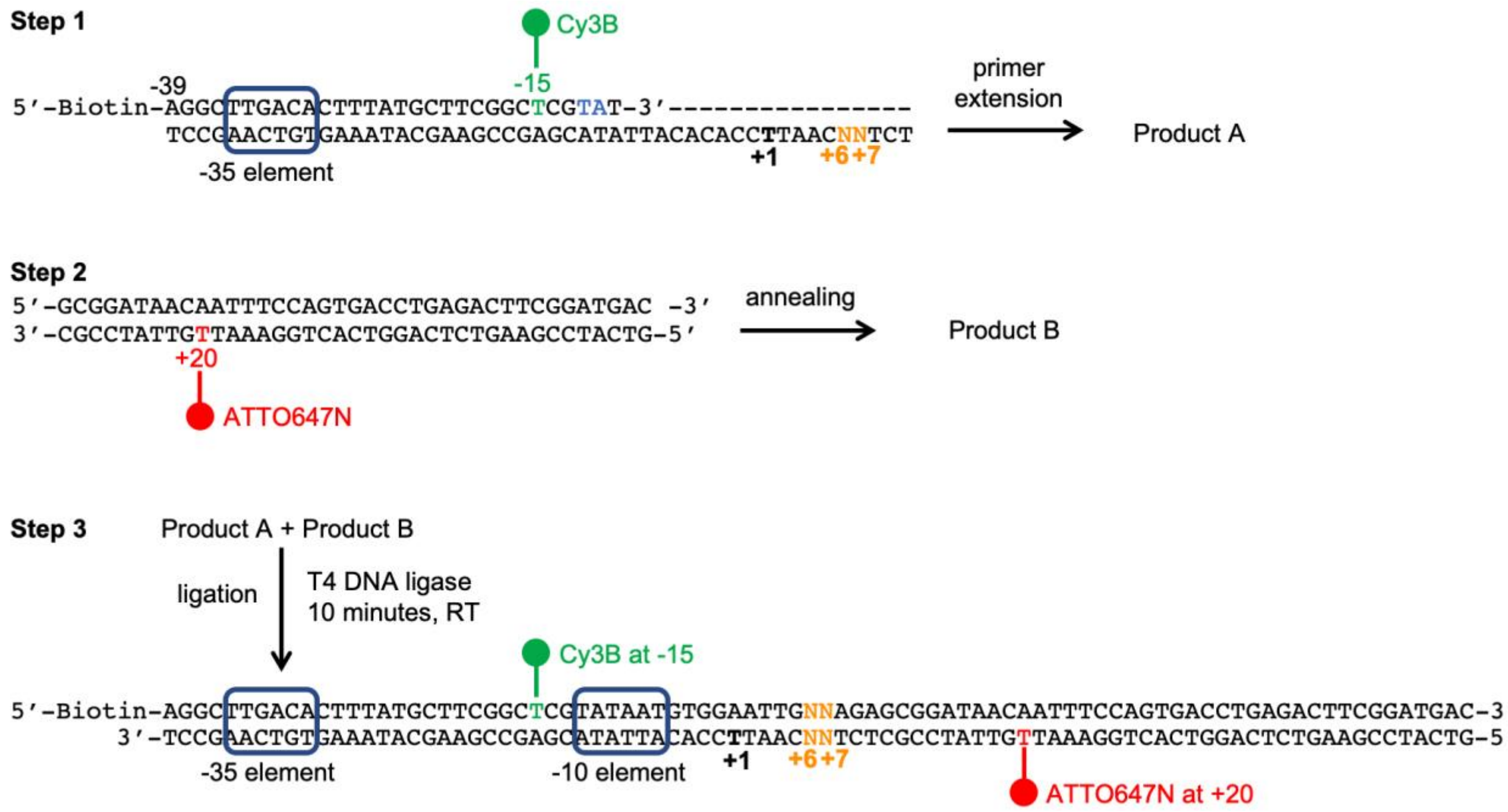


**Supplementary Figure 20. Preparation of library of 16 labelled lacCONS promoter DNA variants.** The library was prepared in 3 steps. **Step 1** involves a primer extension of a Cy3B-modified strand annealed on a template strand with degenerate bases at sites corresponding to positions +6 and +7 from the transcription start site; the extended product is then purified. **Step 2** involves preparation of another duplex fragment, wherein the bottom strand is labelled with ATTO647N at the site corresponding to position +20 form the transcription start site. In **Step 3**, the two dsDNA fragments are ligated using a high-performance T4 DNA ligase to form the final doubly labelled promoter dsDNA fragment.

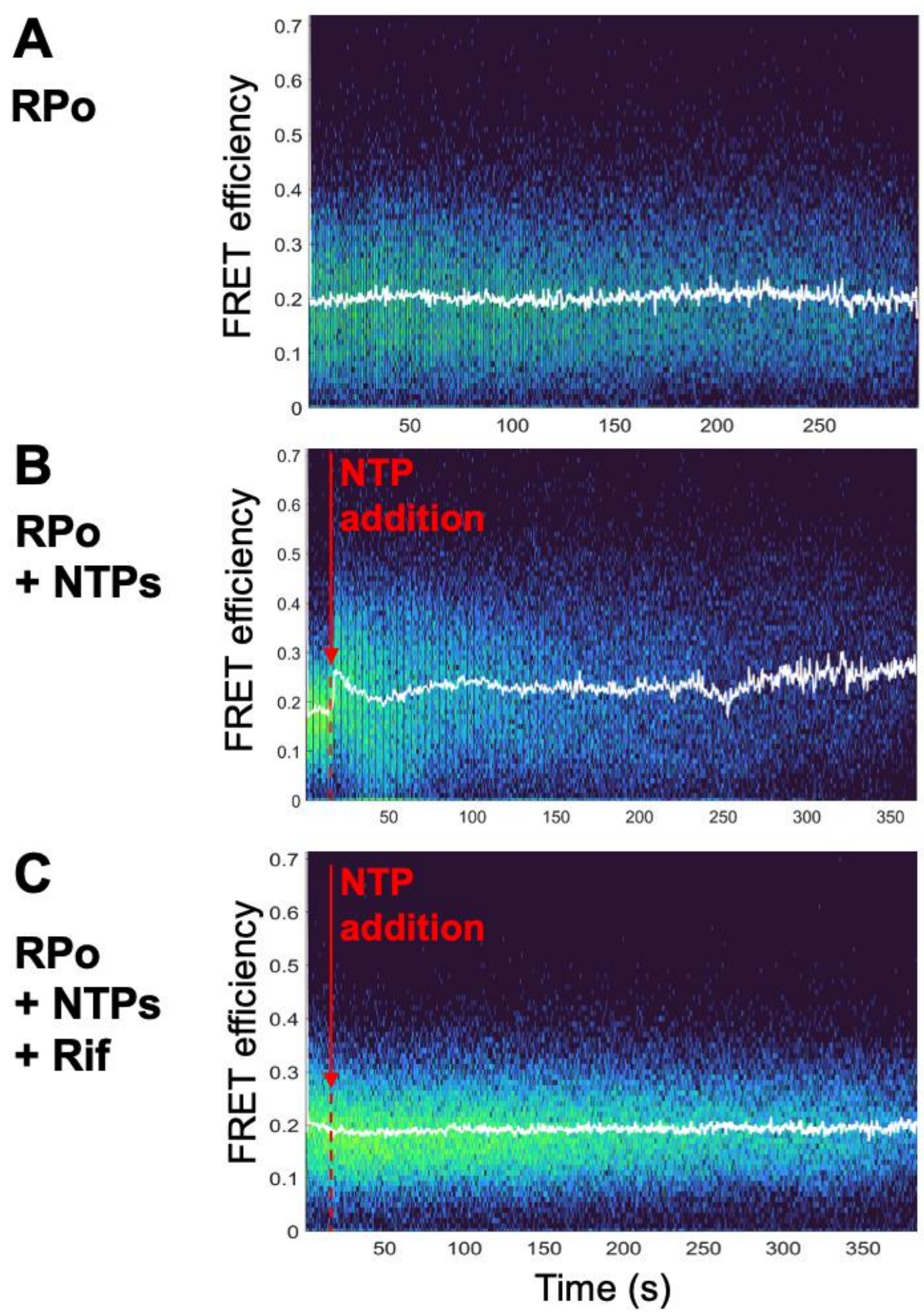


**Supplementary Figure 21. Time evolution of FRET efficiencies for transcription complexes at different conditions.** FRET time-traces have been superimposed to produce heat maps that show the time evolution of the FRET signals.

**A.** Open complexes (RPo) prepared using the labelled promoter library and RNAP were immobilized on surface and imaged using alternating-laser excitation to obtain their $E_{FRET}$. The mean $E_{FRET}$ was ~0.22 and does not change during the recording time. N=112.

**B.** Upon adding 500 µM ApA and 200 µM of all NTPs to the surface-immobilized RPo complexes, a large fraction of the immobilised RPo show an increase in their FRET efficiency at ~15 sec due to the transcription reaction; the following FRET decrease (to a minimum reached in ~50 sec) is due to processes that reduce FRET by populating transcription complexes with $E_{FRET}$ of ~0.2 (mainly abortive RNA release, RNA backtracking and promoter escape; see **Fig. 7A**). The FRET signal also reaches a second local maximum at ~100 sec, likely reflecting a second round of RNA synthesis; beyond that time, fluctuations are lost due to the desynchronisation of the transcribing complexes. Red arrow and line: NTP addition point. N=159.

**C.** Blocking transcription using rifampicin leads to a profile and mean $E_{FRET}$ similar to RPo, confirming that the FRET changes in the presence of NTPs are due to the transcription. N=117.

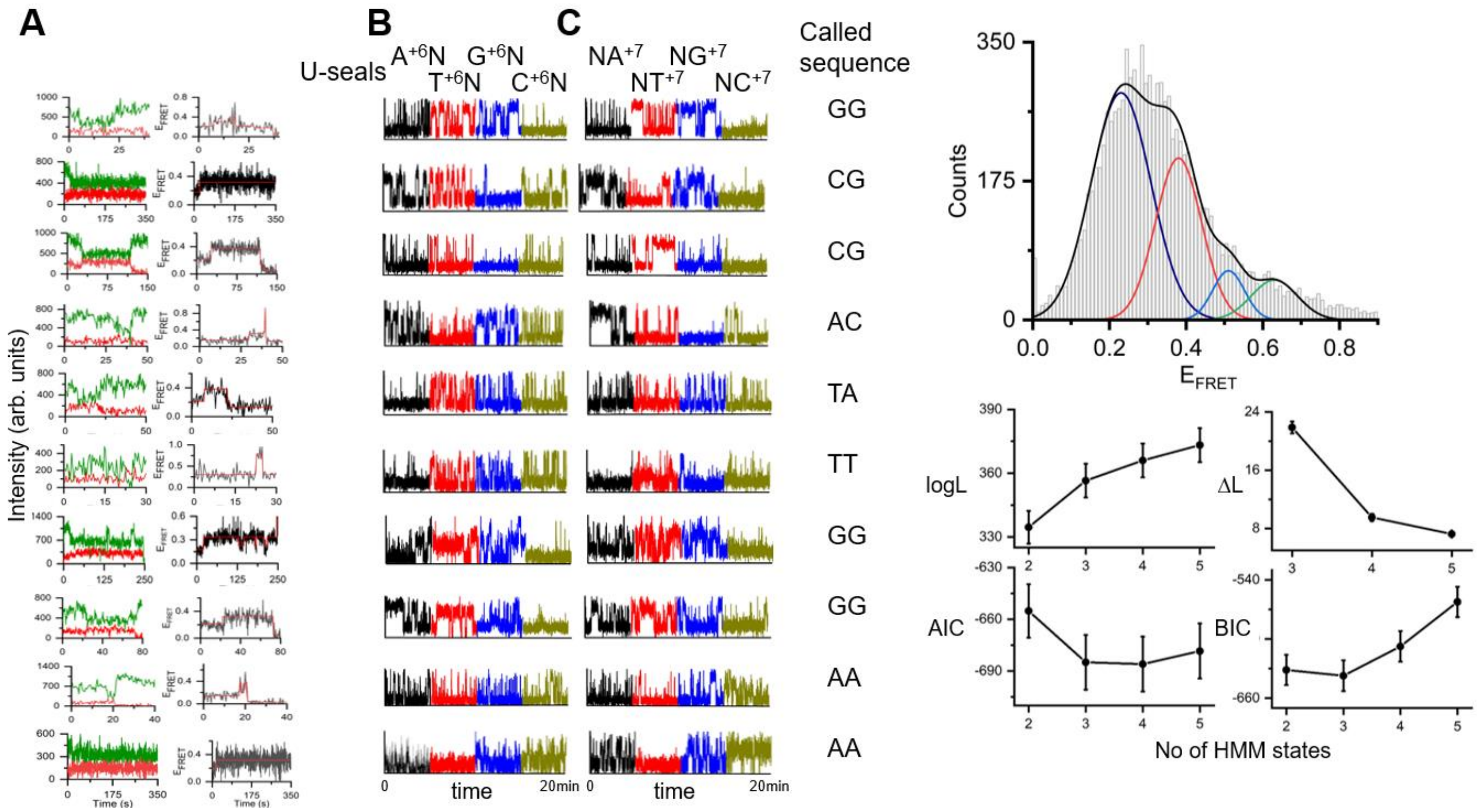


**Supplementary Figure 22. smFRET analysis for the promoter variant library.**

**A-C. Example time-traces obtained from SPIN-seq analysis of the promoter library. A. Left,** FRET donor and FRET acceptor intensities upon donor excitation exhibiting anti-correlated changes characteristic of FRET interactions between the two fluorophores. **Right,** FRET efficiency time-traces showing transitions between a low-FRET state ($E_{FRET}$ ~0.2) and an intermediate-FRET state ($E_{FRET}$~0.4).

**B.** Sequencing position +6 using a R-seal (featuring $N^{+6}N^{+7}$ bases) and a set of competing U-seals ($A^{+6}N^{+7}$, $T^{+6}N^{+7}$, $G^{+6}N^{+7}$, $C^{+6}N^{+7}$).

**C.** Similar interrogation at position +7 for the same molecule using the same R-seal and another set of competing U-seals ($N^{+6}A^{+7}$, $N^{+6}T^{+7}$, $N^{+6}G^{+7}$, $N^{+6}C^{+7}$). In the right panel identified sequence for the same corresponding molecules.

**D.** FRET histogram for all traces showing corresponding to escape events (i.e., the only ones showing transitions that sampled the $E_{FRET}$~0.6 state) prior to donor or acceptor bleaching. The distribution was fitted using a sum of four Gaussian distributions centred at E* ~ 0.22, 0.38, 0.51, and 0.63.

**E.** Identifying the number of FRET states that fits best to the data. To evaluate the fit quality, we used the metrics of log-likelihood logL and change in log-likelihood ΔL (top panels). Model complexity was also evaluated using the AIC and BIC metrics (bottom panels). These results indicate that the three-state system is the dominant behaviour, while a four-state model captures a less abundant FRET state at ~0.5 efficiency.

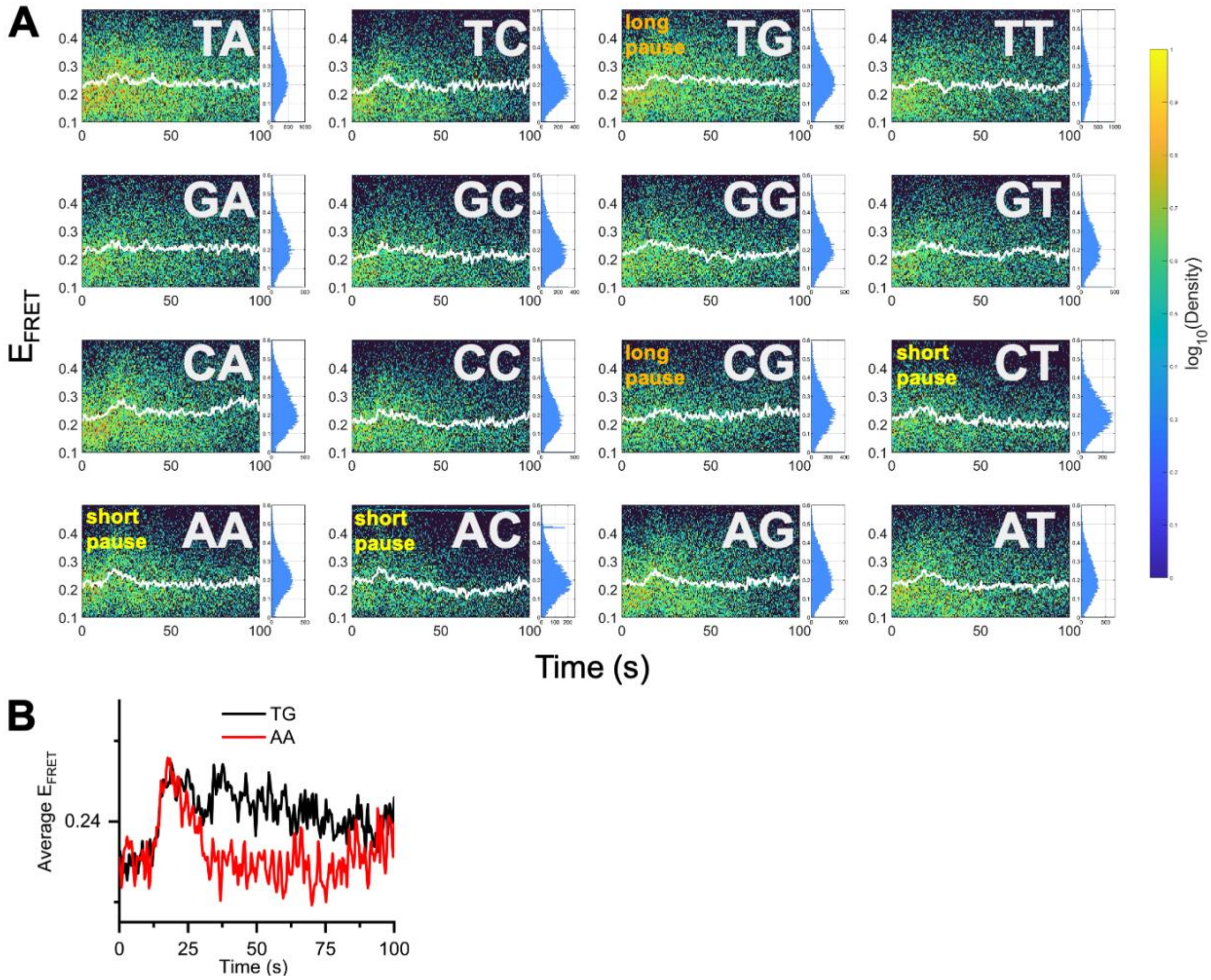


**Supplementary Figure 23. Sequence-dependence of FRET changes along the transcription reaction for the lacCONS promoter 16-variant library.**

**A.** FRET timetrace heatmap featuring traces for all transcriptionally active molecules for all 16 sequences. White line: mean FRET as a function of time. Sequences exhibiting frequent and/or long dwells at the paused state with E*~0.4 (e.g., TG and CG) reach a maximum mean value after 15-20 sec and show a very slow decrease from this value thereafter. Sequences exhibiting short/infrequent pausing (e.g., CT, AA, AC) reach a maximum mean value within 15-20 sec but return to the starting value more rapidly (in 10-20 sec). Sequences exhibiting elevated propensity for promoter escape (class I, e.g., TC) and cycling (class II, e.g., AA) behavior show a distinctive feature of rapid FRET increase to the max FRET value, followed by a rapid decrease to the initial FRET value.

**B.** Overlayed average FRET efficiencies across all traces for two sequences, TG (black) and AA (red). Both the sequences exhibit similar temporal profile in terms of reaching high $E_{FRET}$ value upon addition of NTP (~15 sec). Escalated pausing sequences TG retains the high $E_{FRET}$ value throughout the duration. However, AA sequences show a decrease in $E_{FRET}$ after some time.

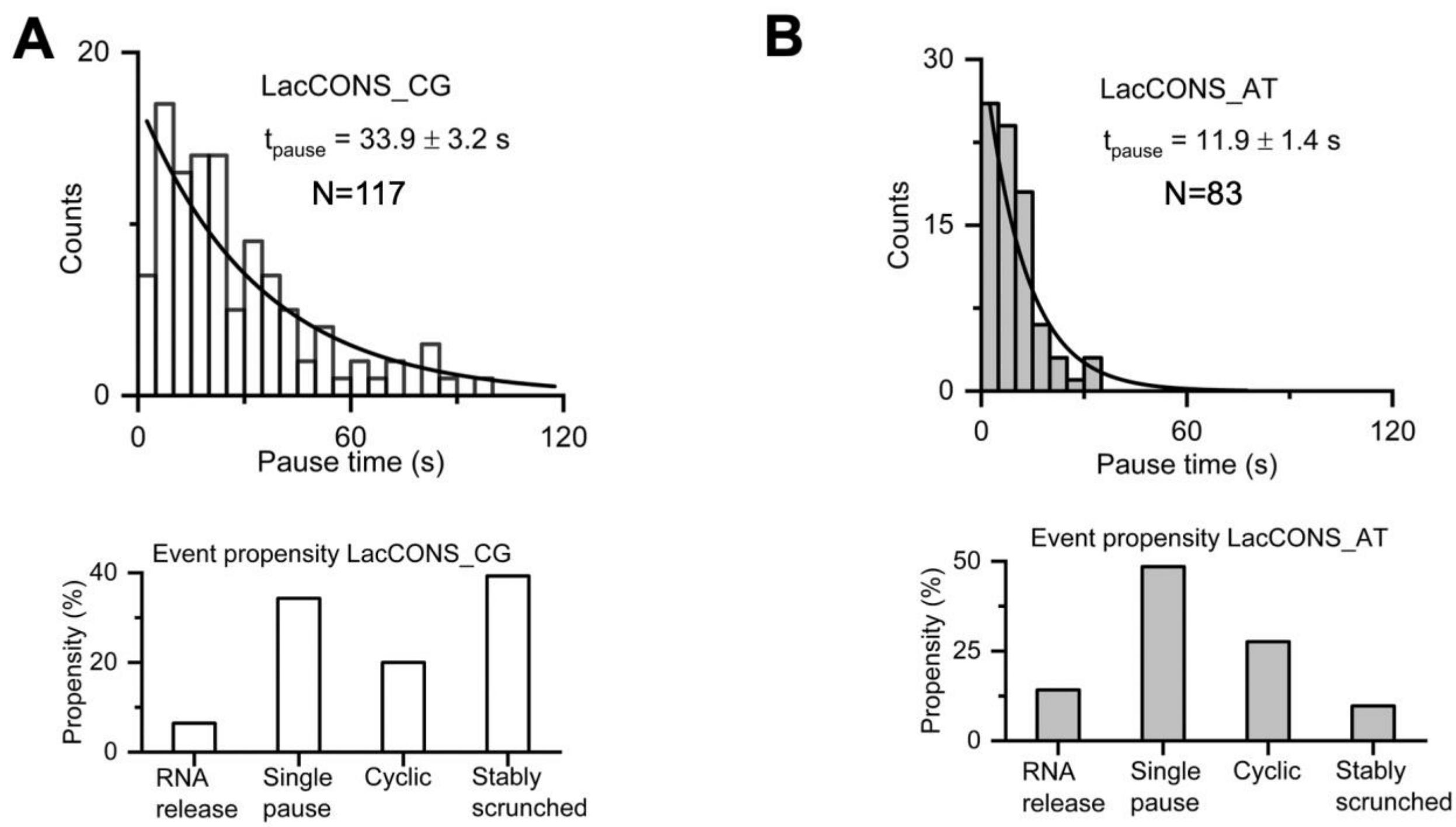


**Supplementary Figure 24. Verification of sequence-dependent effects on transcription initiation.** Analysis of two lacCONS promoter variants labelled at -15 and +20 position with Cy3B and ATTO647N, respectively (as for the promoter library). Variants lacCONS-CG and lacCONS-AT were associated with long and short pause duration, respectively, as seen using SPIN-Seq.

**A.** Pause-duration distribution (top) and distribution between classes (bottom) for variant lacCONS-CG (having C and G at positions +6 and +7, respectively). Single-exponential fitting analysis shows of pause lifetime of ~34 s. The variant is associated with pronounced tendency for stable scrunching and single-pause events.

**B.** Pause-duration distribution (top) and distribution between classes (bottom) for variant lacCONS-AT (having A and T at positions +6 and +7, respectively). Single-exponential fitting analysis shows of pause lifetime of ~12 s. The variant is associated with tendency for cyclic events and single-pause events.

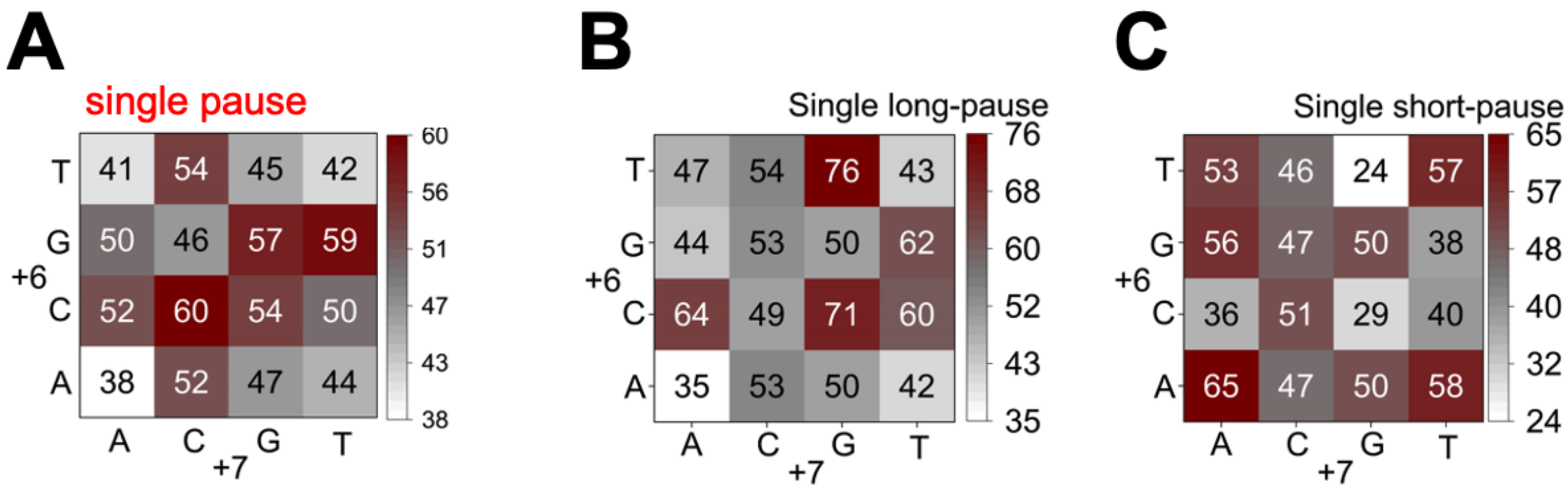


**Supplementary Figure 25. Propensity for promoter escape and single-pause events showing pauses of substantially different duration.**

**A.** Propensity of 16 lacCONS promoters to give Class-III events ("single-pause" events). The propensity is shown as percentage of molecules in the population belonging to this class.

**B.** Percentage of Class-III events that show a single long-pause (traces showing >10 s single pause duration).

**C.** Percentage of Class-III events that show a single short-pause (traces showing 1-10 s single pause duration).

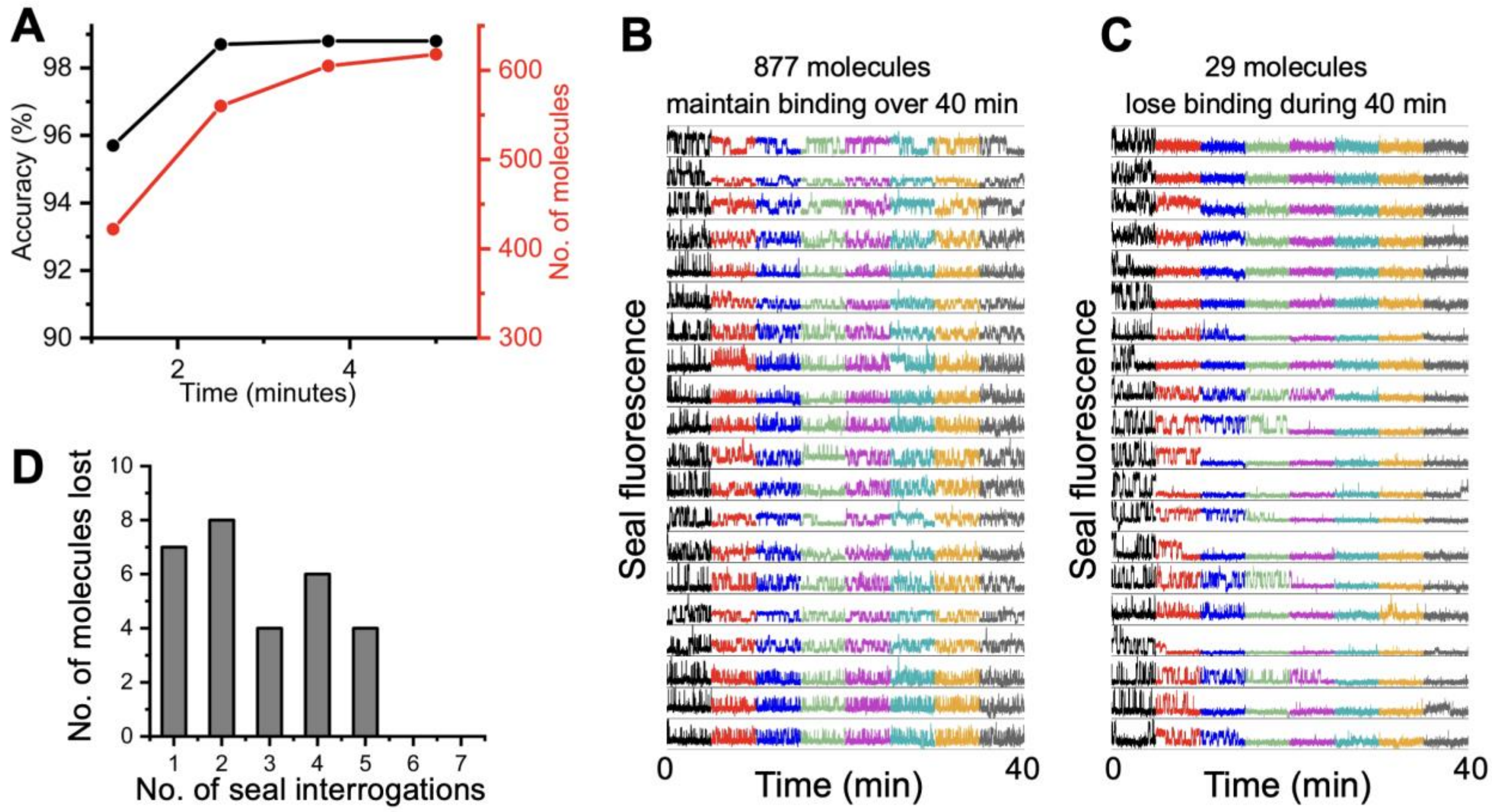


**Supplementary Figure 26. Assay performance as a function of interrogation times. A.** Base-calling accuracy as a function of interrogation time. Accuracy was evaluated for detecting base 3 on the Gap8-$G^3$ template using the non-competitive mode. The time refers to the duration of each interrogation with one of the 4 seals. **B-C.** Binding capability of DNA molecules over repeated interrogations. Gap-$A^3$ molecules were probed using an ATTO647N-labeled complementary strand (seal $T^3$) across eight interrogation cycles, each lasting 5 min, followed by a washing step; 877 molecules (~97%) exhibited characteristic binding–unbinding events across the interrogation traces (examples in panel B), while 29 molecules (~3%) lost the binding–unbinding signature during one of the interrogation cycles (examples in panel C). **D.** Number of lost molecules per interrogation cycle.

## Supplementary Table 1. DNA sequences used in this work.

| Experiments/construct | Strand Name | Sequence |
|---|---|---|
| Formation of 8-nt gapped DNA (Fig. 2A, 2C, and 2D) | Gap-(1-65)-$G^{30}$-$B^{Bio,1}$ | 5’ CAG TCA AGA GCC TGA GGC AGC AGA TTA TCG ACG GTG GAT GTA TGG TAA TGG GAC GAA GAA TGA GG–biotin 3’ |
| | Gap-(1-27)-$Top^{Cy3B, 18}$ | 5’ CCT CAT TCT TCG TCC CA**T(Cy3B)** TAC CAT ACA 3’ |
| | Gap-(36-65)-Top | 5’ CGA TAA TCT GCT GCC TCA GGC TCT TGA CTG 3’ |
| Template for formation of 8-nt gapped DNA with T at position 3 of the gap (Fig. 2C and 2D) | Gap-(1-65)-$T^{30}$-$B^{Bio,1}$ | 5’ CAG TCA AGA GCC TGA GGC AGC AGA TTA TCG ACG GTT GAT GTA TGG TAA TGG GAC GAA GAA TGA GG–biotin 3’ |
| Template for formation of 8-nt gapped DNA with A at position 3 of the gap (Fig. 2C and 2D) | Gap-(1-65)-$A^{30}$-$B^{Bio,1}$ | 5’ CAG TCA AGA GCC TGA GGC AGC AGA TTA TCG ACG GTA GAT GTA TGG TAA TGG GAC GAA GAA TGA GG–biotin 3’ |
| Template for formation of 8-nt gapped DNA with C at position 3 of the gap (Fig. 2C and 2D) | Gap-(1-65)-$C^{30}$-$B^{Bio,1}$ | 5’ CAG TCA AGA GCC TGA GGC AGC AGA TTA TCG ACG GTC GAT GTA TGG TAA TGG GAC GAA GAA TGA GG–biotin 3’ |
| Template for formation of 8-nt gapped DNA with A at all gap positions (Supplementary Figure 5) | Gap-(1-65)-AAAAAAAA-$B^{Bio,1}$ | 5’ CAG TCA AGA GCC TGA GGC AGC AGA TTA TCG AAA AAA AAT GTA TGG TAA TGG GAC GAA GAA TGA GG–biotin 3’ |
| 8 nt fluorescent seals (Fig. 2A, 2C, and 2D) | S8-$C^3$ | 5’ATTO647N -TCC ACC GT 3’ |
| | S8-$G^3$ | 5’ATTO647N -TCG ACC GT 3’ |
| | S8-$A^3$ | 5’ATTO647N -TCA ACC GT 3’ |
| | S8-$T^3$ | 5’ATTO647N -TCT ACC GT 3’ |
| 8 nt fluorescent seals for all-A-gap experiment (Supplementary Figure 5) | S8-allAgap-$C^3$ | 5’ATTO647N -TTC TTT TT 3’ |
| | S8-allAgap-$G^3$ | 5’ATTO647N -TTG TTT TT 3’ |
| | S8-allAgap-$A^3$ | 5’ATTO647N -TTA TTT TT 3’ |
| | S8-allAgap-$T^3$ | 5’ATTO647N -TTT TTT TT 3’ |
| 8-nt R-seal for one-base reading (Fig. 3B) | R8-$N^3$ | 5’ATTO647N -TCN ACC GT 3’ |
| Set of 4 8-nt U-seals for one-base reading (Fig. 3B) | U8-$A^3$ | 5’ TCA ACC GT 3’ |
| | U8-$T^3$ | 5’ TCT ACC GT 3’ |
| | U8-$G^3$ | 5’ TCG ACC GT 3’ |
| | U8-$C^3$ | 5’ TCC ACC GT 3’ |
| Formation of 13-nt gap gapped DNA (Fig. 3E) | Gap-(1-65)-$B^{Bio,1}$ | 5’ CAG TCA AGA GCC TGA GGC AGC AGA TTA TCG ACG GTG GAT GTA TGG TAA TGG GAC GAA GAA TGA GG–biotin 3’ |
| | Gap-(1-27)-$Top^{Cy3B, 25}$ | 5’ CCT CAT TCT TCG TCC CA**T(Cy3B)** TAC CAT ACA 3’ |
| | Gap-(41-65)-Top | 5’ A TCT GCT GCC TCA GGC TCT TGA CTG 3’ |
| 13-nt R-seal for 3-base reading (Fig. 3E) | R13-$N^5N^6N^7$ | 5’ - Atto647N **-**TCC ANN NTC GAT A- BHQ1 - 3’ |
| Set of 12 13-nt U-seals for 3-base reading (Fig. 3E) | U13-$A^5N^6N^7$ | 5’ TCC AAN NTC GAT A 3’ |
| | U13-$T^5N^6N^7$ | 5’ TCC ATN NTC GAT A 3’ |
| | U13-$G^5N^6N^7$ | 5’ TCC AGN NTC GAT A 3’ |

| | | |
|---|---|---|
| | U13-$C^5N^6N^7$ | 5’ TCC ACN NTC GAT A 3’ |
| | U13-$N^5A^6N^7$ | 5’ TCC ANA NTC GAT A 3’ |
| | U13-$N^5T^6N^7$ | 5’ TCC ANT NTC GAT A 3’ |
| | U13-$N^5G^6N^7$ | 5’ TCC ANG NTC GAT A 3’ |
| | U13-$N^5C^6N^7$ | 5’ TCC ANC NTC GAT A 3’ |
| | U13-$N^5N^6A^7$ | 5’ TCC ANN ATC GAT A 3’ |
| | U13-$N^5N^6T^7$ | 5’ TCC ANN TTC GAT A 3’ |
| | U13-$N^5N^6G^7$ | 5’ TCC ANN GTC GAT A 3’ |
| | U13-$N^5N^6C^7$ | 5’ TCC ANN CTC GAT A 3’ |
| 13-nt R-seal for 5-base reading (Fig. 3G) | R13-$N^5N^6N^7N^8N^9$ | 5’- ATTO647N **-**TCC ANN NNN GAT A- BHQ1 - 3’ |
| Set of 20 13-nt U-seals for 5-base reading (Fig. 3G) | U13-$A^5N^6N^7N^8N^9$ | 5’ TCC AAN NNN GAT A 3’ |
| | U13-$T^5N^6N^7N^8N^9$ | 5’ TCC ATN NNN GAT A 3’ |
| | U13-$G^5N^6N^7N^8N^9$ | 5’ TCC AGN NNN GAT A 3’ |
| | U13-$C^5N^6N^7N^8N^9$ | 5’ TCC ACN NNN GAT A 3’ |
| | U13-$N^5A^6N^7N^8N^9$ | 5’ TCC ANA NNN GAT A 3’ |
| | U13-$N^5T^6N^7N^8N^9$ | 5’ TCC ANT NNN GAT A 3’ |
| | U13-$N^5G^6N^7N^8N^9$ | 5’ TCC ANG NNN GAT A 3’ |
| | U13-$N^5C^6N^7N^8N^9$ | 5’ TCC ANC NNN GAT A 3’ |
| | U13-$N^5N^6A^7N^8N^9$ | 5’ TCC ANN ANN GAT A 3’ |
| | U13-$N^5N^6T^7N^8N^9$ | 5’ TCC ANN TNN GAT A 3’ |
| | U13-$N^5N^6G^7N^8N^9$ | 5’ TCC ANN GNN GAT A 3’ |
| | U13-$N^5N^6C^7N^8N^9$ | 5’ TCC ANN CNN GAT A 3’ |
| | U13-$N^5N^6N^7A^8N^9$ | 5’ TCC ANN NAN GAT A 3’ |
| | U13-$N^5N^6N^7T^8N^9$ | 5’ TCC ANN NTN GAT A 3’ |
| | U13-$N^5N^6N^7G^8N^9$ | 5’ TCC ANN NGN GAT A 3’ |
| | U13-$N^5N^6N^7C^8N^9$ | 5’ TCC ANN NCN GAT A 3’ |
| | U13-$N^5N^6N^7N^8A^9$ | 5’ TCC ANN NNA GAT A 3’ |
| | U13-$N^5N^6N^7N^8T^9$ | 5’ TCC ANN NNT GAT A 3’ |
| | U13-$N^5N^6N^7N^8G^9$ | 5’ TCC ANN NNG GAT A 3’ |
| | U13-$N^5N^6N^7N^8C^9$ | 5’ TCC ANN NNC GAT A 3’ |
| DNAs for annealing of CAPcons dsDNA (Fig. 4D) | CAPcons-(1-49)-$C^{25}$-$B^{Bio,49}$ | 5’ biotin-GTG CCT AAA ATG TGA TCT AGA TCA CAT TTA TTG CGT AGA GCT CAC TGC C 3’ |
| | CAPcons-(1-49)- $G^{25}$-$Top^{Cy3B,13}$ | 5’ GGC AGT GAG CTC **T(Cy3B)** AC GCA ATA AAT GTG ATC TAG ATC ACA TTT TAG GCA C 3’ |
| DNAs for annealing of CAPcons dsDNA G:C variant (Fig. 4E) | CAPcons-(1-49)-$G^{25}$-$B^{Bio,49}$ | 5’ biotin-GTG CCT AAA ATG TGA TCT AGA TCA GAT TTA TTG CGT AGA GCT CAC TGC C 3’ |
| | CAPcons-(1-49)-$C^{25}$-$Top^{Cy3B,13}$ | 5' GGC AGT GAG CTC **T(Cy3B)** AC GCA ATA AAT CTG ATC TAG ATC ACA TTT TAG GCA C 3’ |
| DNA for preparation of CAP variant library at position 5 of the CAP consensus (Fig. 4F) | CAPcons-(1-49)-$N^{25}$-$B^{Bio,49}$ | 5’ biotin-GTG CCT AAA ATG TGA TCT AGA TCA NAT TTA TTG CGT AGA GCT CAC TGC C 3’ |
| | CAPcons-(1-20)-$Top^{Cy3B,11}$ | 5’ GGC AGT GAG C**TCy3B)** C TAC GCA AT 3’ |
| Flanking strands for *in situ* gap formation on CAPcons library (Fig. 4F) | CAPcons-(30-49)-Top | 5’ CTA GAT CAC ATT TTA GGC AC 3’ |
| | CAPcons-(1-20)-$Top^{Cy3B,11}$ | 5’ GGC AGT GAG C**T(Cy3B)** C TAC GCA AT 3’ |

| | | |
|---|---|---|
| 9-nt CAPcons R-Seal (Fig. 4H) | R9-CAP-N$^{5}$ | 5’ Cy5 - AAA TNT GAT 3’ |
| Set of 4 9-nt CAPcons U-seals (Fig. 4H) | U9-CAP-A$^{5}$ | 5’ AAA TAT GAT 3’ |
| | U9-CAP-T$^{5}$ | 5’ AAA TTT GAT 3’ |
| | U9-CAP-G$^{5}$ | 5’ AAA TGT GAT 3’ |
| | U9-CAP-C$^{5}$ | 5’ AAA TCT GAT 3’ |
| DNAs for generation of *lac*CONS variant promoter (Fig. 5B) | lacCONS(-39/-10)-Top$^{Cy3B,-15;\ Bio,-39}$ | 5’ biotin- AGG CTT GAC ACT TTA TGC TTC GGC **T(Cy3B)** CG TAT 3’ |
| | lacCONS(-39/+10)-N$^{+6}$N$^{+7}$-B | 5’ TCT NNC AAT TCC AAC ACG GCT ACG AGC CGA AGC ATA AAG TG 3’ |
| | lacCONS(+11/+49)-B$^{Atto647N,+20}$ | 5’ GTC ATC CGA AGT CTC AGG TCA CTG GAA AT**T(ATTO647N)** GTT ATC CGC 3’ |
| | lacCONS(+11/+49)-Top | 5’ GCG GAT AAC AAT TTC CAG TGA CCT GAG ACT TCG GAT GAC 3’ |
| Flanking strands for lacCONS gap formation around positions +6/+7 (Fig. 5C) | lacCONS(+11/+49)-B | 5’ GTC ATC CGA AGT CTC AGG TCA CTG GAA ATT GTT ATC CGC 3’ |
| | lacCONS(-39/+2)-B | 5’ TTC CAC ACA TTA TAC GAG CCG AAG CAT AAA GTG TCA AGC CT 3’ |
| 8-nt lacCONS R-seal (Fig. 5C) | R8-lacCONS(+3/+9)-N$^{+7}$N$^{+6}$-B | 5’ ATTO647N-TCT NNC AA 3’ |
| Set of 8 8-nt lacCONS U-seals (Fig. 5C) | U8-lacCONS(+3/+9) -A$^{+7}$-N$^{+6}$-B | 5’ TCT ANC AA 3’ |
| | U8-lacCONS(+3/+9) -T$^{+7}$-N$^{+6}$-B | 5’ TCT TNC AA 3’ |
| | U8-lacCONS(+3/+9) -G$^{+7}$-N$^{+6}$-B | 5’ TCT GNC AA 3’ |
| | U8-lacCONS(+3/+9) -C$^{+7}$-N$^{+6}$-B | 5’ TCT CNC AA 3’ |
| | U8-lacCONS(+3/+9) -N$^{+7}$-A$^{+6}$-B | 5’ TCT NAC AA 3’ |
| | U8-lacCONS(+3/+9) -N$^{+7}$-T$^{+6}$-B | 5’ TCT NTC AA 3’ |
| | U8-lacCONS(+3/+9) -N$^{+7}$-G$^{+6}$-B | 5’ TCT NGC AA 3’ |
| | U8-lacCONS(+3/+9) -N$^{+7}$-C$^{+6}$-B | 5’ TCT NCC AA 3’ |
| DNAs for preparation of *lac*CONS A$^{+6}$T$^{+7}$ variant (Supplementary Figure 24) | lacCONS(-39/+10)-A$^{+6}$T$^{+7}$-B$^{Cy3B,-15;\ Bio,-39}$ | 5’ Biotin-AGG CTT GAC ACT TTA TGC TTC GGC **T(Cy3B)** CG TAT AAT GTG TGG AAT TGA TAG A 3’ |
| | lacCONS(-39/+10)-A$^{+6}$T$^{+7}$-Top | 5’ TCT ATC AAT TCC ACA CAT TAT ACG AGC CGA AGC ATA AAG TGT CAA GCC T 3’ |
| DNAs for preparation of *lac*CONS C$^{+6}$G$^{+7}$ variant (Supplementary Figure 24) | lacCONS(-39/+10)-C$^{+6}$G$^{+7}$- B$^{Cy3B,-15;\ Bio,-39}$ | 5’ Biotin - AGG CTT GAC ACT TTA TGC TTC GGC **T(Cy3B)** CG TAT AAT GTG TGG AAT TGC GAG A 3’ |
| | lacCONS(-39/+10)-C$^{+6}$G$^{+7}$-Top | 5’ TCT CGC AAT TCC ACA CAT TAT ACG AGC CGA AGC ATA AAG TGT CAA GCC T 3’ |

Underlined sequences denote the part of a long template strand that forms the ssDNA gap in a gapped DNA. Bold typeface denotes a labelled position along with the fluorophore attached (the fluorophores used are in a parenthesis). N indicates a degenerate base. Top: top strand.  B: bottom strand. Bio: biotin.

**Supplementary Table 2. Accuracies for base-calling for the competitive-inhibition-based assay**

| **Accuracy for single-base sequencing using the competitive-inhibition assay** | | |
|---|---|---|
| **Gap Base** | **Accuracy (%), individual gaps** | **Accuracy (%) mixed gaps** |
| A | 94.2 (274/291) | 97.9 (184/188) |
| T | 97.2 (383/394) | 98.8 (243/246) |
| G | 99.6 (500/502) | 99.6 (224/225) |
| C | 96.6 (736/762) | 96.9 (190/196) |
| All bases | 97.1 (1893/1949) | 98.5 (841/855) |
| **Accuracy for 3-base sequencing using the competitive-inhibition assay** | | |
| **Gap Base** | **Accuracy (%)** | |
| $G^5$ | 96.4 (240/249) | |
| $G^6$ | 98.1 (256/261) | |
| $C^7$ | 95.0 (489/515) | |
| All bases | 96.5 (765/793) | |
| **Accuracy for 5-base sequencing using the competitive binding assay** | | |
| **Gap Base** | **Accuracy (%)** | |
| $G^5$ | 95.1 (195/205) | |
| $G^6$ | 97.9 (464/474) | |
| $C^7$ | 97.1 (438/451) | |
| $A^8$ | 94.9 (352/371) | |
| $G^9$ | 97.1 (233/240) | |
| All bases | 96.6 (1682/1741) | |

**Supplementary Table 3. Kinetic analysis of CAP-DNA interactions based on single-molecule binding time-traces.**

| **Kinetic analysis for CAP binding to consensus and single mutant DNA sequences** | | | |
|---|---|---|---|
| **Sequence** | **Dwell times $t_1$ and $t_2$ (s)** | **On-rate ($M^{-1}s^{-1}$)** | **$K_d$ (nM)** |
| CAPcons_G | 0.55 ± 0.31<br>6.42 ± 0.35 | 6.8 x $10^6$ | 20.2 ± 1.3 |
| CAPcons_C (consensus) | 1.75 ± 0.42<br>8.71 ± 0.50 | 1.3 x $10^7$ | 9.1 ± 0.6 |
| **Kinetic analysis for CAP-binding to the library of CAPcons DNA variants** | | | |
| **Sequence** | **Dwell times $t_1$ and $t_2$ (s)** | **On-rate ($M^{-1}s^{-1}$)** | **$K_d$ (nM)** |
| CAPcons_A | 0.90 ± 0.13<br>4.34 ± 0.09 | 8.11±0.29 x $10^6$ | 29.1 ± 1.6 |
| CAPcons_T | 0.80 ± 0.09<br>5.04 ± 0.25 | 8.20±0.46 x $10^6$ | 24.4 ± 0.8 |
| CAPcons_G | 0.85 ± 0.16<br>6.02 ± 0.14 | 8.36±0.41 x $10^6$ | 20.1 ± 0.9 |
| CAPcons_C (consensus) | 1.27 ± 0.19<br>8.78 ± 0.50 | 11.09±0.96 x $10^6$ | 10.6 ± 0.6 |

**Supplementary Table 4. List of buffers for sequencing within gaps with different sizes.**

| Assay | Gap length | Buffer | Buffer composition |
|---|---|---|---|
| Non-competitive assay | 8 nt | IB2 | 20 mM HEPES, pH 7.7, 200 mM NaCl, 80 mM $MgCl_2$, 5% dextran sulphate, 10% formamide, 1 mM TROLOX, 1% Glucose, 40 µg/ml catalase and 0.1 mg/ml glucose oxidase |
| Competitive-inhibition assay | 8 nt | IB3 | 20 mM HEPES pH 7.7, 200 mM NaCl, 80 mM $MgCl_2$, 10% Dextran Sulphate, 10% formamide, 1 mM TROLOX, 1% Glucose, 40 µg/ml catalase, 0.1 mg/ml glucose oxidase |
| Competitive-inhibition assay | 13 nt | IB3 | 20 mM HEPES pH 7.7, 200 mM NaCl, 80 mM $MgCl_2$, 10% Dextran Sulphate, 10% formamide, 1 mM TROLOX, 1% Glucose, 40 µg/ml catalase, 0.1 mg/ml glucose oxidase |
| Competitive-inhibition assay | 8 nt (transcription pausing experiments, Fig. 7) | IB4 | 20 mM HEPES pH 7.7, 200 mM NaCl, 150 mM $MgCl_2$, 10% dextran sulphate, 5% formamide, 200 µM of freshly prepared spermidine hydrochloride, 1 mM TROLOX, 1% Glucose, 40 µg/ml catalase, 0.1 mg/ml glucose oxidase |

## Supplementary references


1. Kapanidis, A. N., Ebright, Y. W., Ludescher, R. D., Chan, S. & Ebright, R. H. Mean DNA bend angle and distribution of DNA bend angles in the CAP-DNA complex in solution. *J. Mol. Biol.* **312**, 453–468 (2001).
2. Uphoff, S. *et al.* Monitoring multiple distances within a single molecule using switchable FRET. *Nat. Methods* **7**, 831–836 (2010).
3. Kapanidis, A., Ebright, Y. & Ebright, R. Site-specific incorporation of fluorescent probes into protein: hexahistidine-tag-mediated fluorescent labeling with (Ni(2+):nitrilotriacetic Acid (n)-fluorochrome conjugates. *JACS* **123**, 12123–12125 (2001).
4. Kapanidis, A. N. *et al.* Fluorescence-aided molecule sorting: analysis of structure and interactions by alternating-laser excitation of single molecules. *Proc. Natl. Acad. Sci. U. S. A.* **101**, 8936–8941 (2004).
5. Kapanidis, A. N., Ebright, Y. W., Ludescher, R. D., Chan, S. & Ebright, R. H. Mean DNA bend angle and distribution of DNA bend angles in the CAP-DNA complex in solution. *J. Mol. Biol.* **312**, (2001).
6. Belogurov, G. A. *et al.* Structural basis for converting a general transcription factor into an operon-specific virulence regulator. *Mol. Cell* **26**, 117–29 (2007).
7. Hudson, B. P. *et al.* Three-dimensional EM structure of an intact activator-dependent transcription initiation complex. *Proc. Natl. Acad. Sci. U. S. A.* **106**, 19830–19835 (2009).
8. Duchi, D. *et al.* RNA Polymerase Pausing during Initial Transcription. *Mol. Cell* **63**, (2016).
9. Dulin, D. *et al.* Pausing controls branching between productive and non-productive pathways during initial transcription in bacteria. *Nat. Commun.* **9**, (2018).
10. Wang, A. *et al.* The displacement of the σ70 finger in initial transcription is highly heterogeneous and promoter-dependent. *Nucleic Acids Res.* **53**, (2025).
11. Malinen, A. M. *et al.* Real-Time Single-Molecule Studies of RNA Polymerase–Promoter Open Complex Formation Reveal Substantial Heterogeneity Along the Promoter-Opening Pathway. *J. Mol. Biol.* **434**, 167383 (2022).
12. Owczarzy, R., Moreira, B. G., You, Y., Behlke, M. A. & Wälder, J. A. Predicting stability of DNA duplexes in solutions containing magnesium and monovalent cations. *Biochemistry* **47**, (2008).
13. Wahl, G. M., Stern, M. & Stark, G. R. Efficient transfer of large DNA fragments from agarose gels to diazobenzyloxymethyl-paper and rapid hybridization by using dextran sulfate. *Proceedings of the National Academy of Sciences* **76**, 3683–3687 (1979).
14. Ku, W.-C., Lau, W. K., Tseng, Y.-T., Tzeng, C.-M. & Chiu, S.-K. Dextran sulfate provides a quantitative and quick microarray hybridization reaction. *Biochem. Biophys. Res. Commun.* **315**, 30–37 (2004).
15. Kummerlin, M. *et al.* Tunable fluorogenic DNA probes drive fast and high-resolution single-molecule fluorescence imaging. *Nucleic Acids Res.* **53**, gkaf593 (2025).
16. Ma, H., Chen, M., Nguyen, P. & Liu, Y. Toward drift-free high-throughput nanoscopy through adaptive intersection maximization. *Sci. Adv.* **10**, eadm7765 (2024).
17. Thévenaz, P., Ruttimann, U. E. & Unser, M. A pyramid approach to subpixel registration based on intensity. *IEEE Transactions on Image Processing* **7**, 27–41 (1998).
18. Schnitzbauer, J., Strauss, M. T., Schlichthaerle, T., Schueder, F. & Jungmann, R. Super-resolution microscopy with DNA-PAINT. *Nat. Protoc.* **12**, 1198–1228 (2017).
