# Supplementary material for: Unraveling single-molecule reactions via multiplexed in-situ DNA sequencing
Source: Nat Commun. 2026 Aug 29;17:10639. doi: 10.1038/s41467-026-77308-3 (PMC13649778; doi:10.1038/s41467-026-77308-3)
Supplement: Supplementary file 2 [file 41467_2026_77308_MOESM2_ESM.pdf]

nature portfolio

Corresponding author(s): Dr. Achillefs Kapanidis
Last updated by author(s): 26/06/2026

Reporting Summary

Nature Portfolio wishes to improve the reproducibility of the work that we publish. This form provides structure for consistency and transparency in reporting. For further information on Nature Portfolio policies, see our Editorial Policies and the Editorial Policy Checklist.

Statistics

For all statistical analyses, confirm that the following items are present in the figure legend, table legend, main text, or Methods section.

n/a	Confirmed	
☐	☒	The exact sample size (*n*) for each experimental group/condition, given as a discrete number and unit of measurement
☐	☒	A statement on whether measurements were taken from distinct samples or whether the same sample was measured repeatedly
☒	☐	The statistical test(s) used AND whether they are one- or two-sided<br>*Only common tests should be described solely by name; describe more complex techniques in the Methods section.*
☒	☐	A description of all covariates tested
☒	☐	A description of any assumptions or corrections, such as tests of normality and adjustment for multiple comparisons
☐	☒	A full description of the statistical parameters including central tendency (e.g. means) or other basic estimates (e.g. regression coefficient) AND variation (e.g. standard deviation) or associated estimates of uncertainty (e.g. confidence intervals)
☒	☐	For null hypothesis testing, the test statistic (e.g. *F*, *t*, *r*) with confidence intervals, effect sizes, degrees of freedom and *P* value noted<br>*Give P values as exact values whenever suitable.*
☐	☒	For Bayesian analysis, information on the choice of priors and Markov chain Monte Carlo settings
☒	☐	For hierarchical and complex designs, identification of the appropriate level for tests and full reporting of outcomes
☒	☐	Estimates of effect sizes (e.g. Cohen's *d*, Pearson's *r*), indicating how they were calculated

Our web collection on statistics for biologists contains articles on many of the points above.

Software and code

Policy information about availability of computer code

Data collection	All data collection protocol described in methods. All imaging movies were collected using ONI nanoimager microscope using nanoimager software.
Data analysis	Detailed protocol of data analysis is given in methods section. Imaging movies were processed and analysed using home-made python-based softwares. Movies were drift-corrected and aligned using molseeq (github repository https://github.com/piedrro/napari-molseeq). Molecules were localized using picasso incorporated in molseeq. time-traces were visualized and post-processed using Traceanalyser (https://github.com/piedrro/TraceAnalyser). Localization counting and base calling was performed using python based scripts (https://github.com/Z-Qing/GapSeq-repo). Histograms and graphs were plotted and fitted using OriginPro2021. Images were visualized and exported using fiji (imageJ). Overlay of single-molecule time traces and heatmaps were plotted using home-made MATLAB (MATLAB R2022b) code. All the python based codes and software are deposited together also in https://doi.org/10.5281/zenodo.20848891.

For manuscripts utilizing custom algorithms or software that are central to the research but not yet described in published literature, software must be made available to editors and reviewers. We strongly encourage code deposition in a community repository (e.g. GitHub). See the Nature Portfolio guidelines for submitting code & software for further information.

Data

Policy information about availability of data

All manuscripts must include a data availability statement. This statement should provide the following information, where applicable:

- Accession codes, unique identifiers, or web links for publicly available datasets
- A description of any restrictions on data availability
- For clinical datasets or third party data, please ensure that the statement adheres to our policy

All data supporting the conclusion of this work are in the main and supplementary figures. Raw movies and localization counting files for proof of principle of sequencing method have been deposited in figshare online repository (https://doi.org/10.6084/m9.figshare.31963782). Raw movies for sequencing in transcription assay are available from corresponding authors upon reasonable request. Source data has been provided with the manuscript.

Research involving human participants, their data, or biological material

Policy information about studies with human participants or human data. See also policy information about sex, gender (identity/presentation), and sexual orientation and race, ethnicity and racism.

Reporting on sex and gender	n/a
Reporting on race, ethnicity, or other socially relevant groupings	n/a
Population characteristics	n/a
Recruitment	n/a
Ethics oversight	n/a

Note that full information on the approval of the study protocol must also be provided in the manuscript.

Field-specific reporting

Please select the one below that is the best fit for your research. If you are not sure, read the appropriate sections before making your selection.

☒ Life sciences ☐ Behavioural & social sciences ☐ Ecological, evolutionary & environmental sciences

For a reference copy of the document with all sections, see nature.com/documents/nr-reporting-summary-flat.pdf

Life sciences study design

All studies must disclose on these points even when the disclosure is negative.

Sample size	For all the single-molecule dwell time and localization counting analysis in the proof-of-principle single field of view with more than 1000 attached DNA molecules were considered. Each dwell-time histogram contain more than 1000 binding-unbinding events which should be sufficient for statistical analysis. For sequencing all the sample sizes for single-nucleotide and multiple nucleotides detection are reported in tabular format. For initiation pause experiment for each sequence more than 50 molecules were taken into account. For pause-time histogram generation to check whether number of molecules were sufficient or not we removed 1/3rd of the molecules and determined the pause-time for each sequence and compare with the pause-times for all the molecules. In this case pause duration didn't change more than standard deviation through which we decided that sample-size is enough.
Data exclusions	For single-base sequencing in the non-competition method we excluded molecules with overlapping PSFs.
Replication	All the single-base sequence identification experiments were replicated three times. Multiple base identification were replicated two times. Three base walking experiments were performed three times. CAP-binding and sequencing experiment was replicated two times with the similar condition. For the initiation-pause sequencing experiment twenty transcription-sequencing assays were combined to reach the throughput. This replication was done two times. For the initiation pause of specific sequences for each variant experiment was repeated two times each consists of three transcription assays of similar condition.
Randomization	n/a
Blinding	n/a

Reporting for specific materials, systems and methods

We require information from authors about some types of materials, experimental systems and methods used in many studies. Here, indicate whether each material, system or method listed is relevant to your study. If you are not sure if a list item applies to your research, read the appropriate section before selecting a response.

Materials & experimental systems

n/a	Involved in the study
☒	☐ Antibodies
☒	☐ Eukaryotic cell lines
☒	☐ Palaeontology and archaeology
☒	☐ Animals and other organisms
☒	☐ Clinical data
☒	☐ Dual use research of concern
☒	☐ Plants

Methods

n/a	Involved in the study
☒	☐ ChIP-seq
☒	☐ Flow cytometry
☒	☐ MRI-based neuroimaging

Plants

Seed stocks	n/a
Novel plant genotypes	n/a
Authentication	n/a
