# Supplementary material for: Unraveling single-molecule reactions via multiplexed in-situ DNA sequencing
Source: Nat Commun. 2026 Aug 29;17:10639. doi: 10.1038/s41467-026-77308-3 (PMC13649778; doi:10.1038/s41467-026-77308-3)
Supplement: Supplementary file 3 [file 41467_2026_77308_MOESM3_ESM.pdf]

nature portfolio

Peer Review File

Unraveling single-molecule reactions via multiplexed in-situ DNA sequencing

Corresponding Author: Professor Achillefs Kapanidis

Parts of this Peer Review File have been redacted as indicated to maintain the confidentiality of unpublished data. This file contains all reviewer reports in order by version, followed by all author rebuttals in order by version.

Version 0:

Reviewer comments:

Reviewer #1

(Remarks to the Author)
This manuscript (NCOMMS-25-94084-T, “Unraveling single-molecule reactions via multiplexed in-situ DNA sequencing”) submitted by Hazra et al. introduces SPIN-Seq (Single-molecule Phenotyping and In-situ Sequencing), a novel single-molecule platform that directly links the functional phenotypes of individual DNA molecules to their underlying sequences on the same surface and within the same microscope field of view. The authors develop a transient hybridization-based in situ sequencing strategy that enables accurate reading of up to five nucleotides per molecule and apply this technique to investigate how DNA sequence variations influence transcription factor binding and transcription initiation dynamics by E coli RNA polymerase.
This work integrates substantial methodological innovation with biologically meaningful insights into how DNA sequence information is translated into functional phenotypes in protein-DNA interactions. However, to strengthen this manuscript and improve clarity, additional experiments are required, and several protocols need to be described in greater detail. In particular, the optimization procedures are insufficiently explained. After appropriate revision, this manuscript could be suitable for publication in Nature Communications. Major and minor comments are detailed below.

Major comments
1. Overall, many figures are too small to read clearly. The authors should provide higher-resolution data images and enlarge axis labels and annotations throughout the manuscript.
2. Several dwell time distributions exhibit double-exponential decay. The authors should explain the origin of the double-exponential decay in dwell times and clarify the meanings of the short (t1) and long (t2) dwell time components. This explanation is important for understanding why t2 was chosen for comparing the seal-binding dwell times.
3. Fig. S6B represents dwell times of R-seals depending on U-seal concentrations. The authors should clearly state the conclusion drawn from this analysis. Specifically, what is the optimal concentration of U-seals based on the dwell time data and how was the optimal concentration determined?
In addition, although the authors state that the dwell times were fitted with a double-exponential decay function in Fig. S6B, only the first distribution (top left) appears to be properly fitted. The remaining datasets should be refitted appropriately, and the values of t1, t2 and average dwell time should be displayed.
Along the same line, Fig. S6C appears to use average dwell times. The authors should explicitly define these values as average dwell times and explain why average dwell time was chosen instead of t1 or t2. This same clarification is required for Fig. S7C and S12C.
4. Regarding R-seal/U-seal optimization assays in Fig. S6 and S7, at 0.6 uM U-seal concentration, mismatched U-seals completely suppress R-seal binding in Fig. S7A, while complementary U-seals show similar R-seal binding in Fig. S6A. This result appears counterintuitive, as complementary U-seals would be expected to suppress R-seal binding more efficiently than mismatched U-seals. The authors should explain this observation.
5. It is clever to use formamide to increase transient bindings of R-seals for long gaps. However, although optimization experiments are shown in Fig. S10, different formamide concentrations are used depending on gap size. The authors do not explain how the optimal formamide concentration was determined depending on gap size. The rationale underlying this choice should be described.
6. Fig. S12 appears to address U-seal concentration optimization for R-seal binding of 5-nt sequencing. However, the explanation is insufficient. The criteria for determining the optimal U-seal concentration are not described. Moreover, the binding kinetics in Fig. S12A do not show a consistent trend: some traces show suppression of R-seal binding at high U-seal concentrations, while others do not. Does not the single mismatched U-seal suppress R-seals? Additionally,

mismatched U-seals do not appear to alter dwell times across concentrations. The authors should clarify what conclusions are drawn from Fig. S12.
7. The authors use purified CAP and E coli RNA polymerase. The activity of the purified proteins should be validated using additional biochemical assays, such as EMSA for CAP and in vitro transcription assay for E coli RNA polymerase.
8. Regarding the in-situ hybridization for a gap, the authors report ~70% efficiency for 100 nM flanking strands and >90% efficiency for 1 or 2 uM unlabeled flanking strands. However, the 70% efficiency is measured using only one labeled flanking strand. When both strands must be simultaneously hybridized, the actual gap-formation efficiency is likely lower than 70%. For more accurate quantification, both strands should be labeled with different fluorophores and analyzed using three-color colocalization.
9. For clarity, the authors should provide a clearer description of how the scattered plots for localization maps were generated.
10. The quencher-dye seal strategy demonstrated in Fig. 3E-3H works particularly well for long gaps and significantly reduces background fluorescence. This approach is highly suitable for studying promoter escape by E coli RNA polymerase. These experiments convincingly demonstrate both sequence dependence (Fig. 5H) during transcription initiation and the robustness of both quencher-dye and fluorescent seal strategies.
11. In Fig. 5, the authors claim that C rather than G at +7 from the TSS enhances the promoter escape. In E coli genes or promoters, is cytosine statistically enriched at the +7 position? A genome-wide analysis demonstrating a preference for C over G at this position would significantly strengthen the biological relevance of this finding.

Minor comments
1. In the purification of the E coli RNAP holoenzyme, the location (subunit) of the HIS tag should be specified.
2. Exposure times differ across experiments (Fig. 2A-2B, Fig. 2C-2D, and Figs. 3-4). The authors should explain the rationale for these differences.
3. Depending on the gap size, the imaging buffers vary. For clarity, the authors should provide a summary table listing buffer compositions used for each gap size.
4. In Fig. 3F, the labels “A, G, C, and T” on the x-axis are misaligned and should be corrected.
5. High concentration of (80 mM or 150 mM) MgCl2 and 5~10% of dextran sulphate are used probably to enhance the binding of DNA. These are not usual buffer conditions for DNA hybridization. For readers, the authors need to explain the rationale for using such buffers.
6. Through the manuscript, R-seal concentrations are unclear. The R-seal concentration represents the total concentration of all R-seals? For example, in Fig. 3E, 500 nM is used for 64 R-seals. Then does 500 nM represent the concentration per individual R-seal or the total concentration of all R-seals combined? This should be clarified explicitly.
7. At line 130 on page 4, the sentence “we tracked R-seal binding to the gap while it is sequentially added mixed with one of the four U-seals” is grammatically strange and should be rewritten for clarity.

(Remarks on code availability)

Reviewer #2

(Remarks to the Author)
In this manuscript, Hazra et al. describe a new method called Single-molecule Phenotyping and In-Situ Sequencing (SPIN-Seq). This method allows for measuring a functional output on a single DNA followed by DNA sequencing to correlate the sequence with function at a single-molecule level. They use this method to examine sequence-specific transcription factor binding behavior and sequence dependences of RNA polymerase open complex dynamics. This method is promising and has clear applications to many biological systems. In general, however, I felt that the method needs to be explained in more detail both in the details of the experiments and the analysis so that it can be replicated by other single-molecule labs. I have several questions to help clarify the method and suggestions for additions that would bolster some of their conclusions.
Major concerns/comments:
1. The authors report accuracy for specific sequences, but they do not discuss the robustness for differences in base pairing potential. How robust is the sequencing method relative to base-pairing potential and repetitive sequences? Would the authors show how well their method sequences A-rich or T-rich regions or sequences with low GC-content?
2. Does SPIN-Seq work at higher temperature? How does the accuracy and robustness of the sequencing method change at higher temperature? I could imagine functional assays that require 37C, so it would be important to know if sequencing can be performed at higher temperature as well.
3. The authors claim that this method can sequence 5 bases with high accuracy on the same surface (1024 possible sequences). What would the read depth be for each sequence? How many molecules can they image at once? Even with a large camera field of view around 10,000 molecules, the read depth per sequence would be ~10 molecules. Would the authors comment on this and how they would interpret the functional assay per sequence when the read depth is so low?
4. In figure 2D, the authors show “a map of colocalizations around the interrogated gap DNA molecule”. It is unclear that this is a single DNA molecule examined, so please indicate on the figure with an X or in some way to show where the immobilized gap DNA is located. What is the scale in XY? A localization grid would help show the magnitude of the localization. I cannot tell at this resolution what the scale bar says.
5. For the competitive inhibition assay, would the authors comment on why they chose to label the R-seal and not the U-seal to probe when colocalization decreases as opposed to increases for detection?
Minor comments:
1. A discussion of the advantages, limitations, and comparison to other single-molecule based sequencing methods would be very useful in the conclusions section. For example, how long does the sequencing steps typically take? How does this change as more bases are sequenced? What materials are needed that are consumed in the experiment? Can probes be

reused? What about cost?
2. The description of the base calling analysis in the supplement (starting at line 341) is hard to understand and needs clarification. Why are the authors fitting the first derivative of the PDF (line 353)? How is the threshold determined “algorithmically”? What does it mean that “molecules show persistent localization clustering over time”? Why were localization bundle thresholds of 5 and 2 chosen for single or multiple base calling? What does it mean that the longest bundle should be smaller than 50% of the frame number? Please explain and clarify this section.
3. For the CAP-binding assay analysis, the authors state that “Traces were then fitted using a cluster-based approach for dwell-time analysis”. What is a cluster-based approach? Has this been used previously? Please explain.
4. Do the authors perform each sequential step by hand or do they use a pump system or some kind of microfluidics? If doing the washes by hand, are the traces spliced together on the same time axis with the washing steps removed? Would the authors comment on the any decrease in the density of molecules in a field of view during sequential washing and sequencing steps due to drift or photobleaching?
5. In general, all of the figures have parts that are very small and hard to see. The text in some figures is almost illegible. Please increase sizes and resolution so the reader can inspect them easily.
6. Do the authors typically use multiple fields of view to generate enough data for sequence depth? Was a single field of view used in the non-equilibrium experiments to generate the kinetic data in Figure 5F or was the data aggregated from multiple movies? Please explain.
Typos/Clarity:
1. In the supplement, the immobilization density is reported as 0.2 molecules per mm2 but later in the text it says 0.2 per um2. (Lines 145-152) Which is correct?
2. The authors report TIRF at an angle of 56° (Line 170), but the glass-water critical angle is 63°. Would the authors clarify?
3. Line 334-339: What is the background subtraction referring to? What image registration is being performed?
4. The authors should state that they use a “kernel density estimation” to acquire the probability density function in the supplement (Line 352).

(Remarks on code availability)

Reviewer #3

(Remarks to the Author)
In this work, Hazra et al. demonstrate a new approach to multiplexed single-molecule imaging that utilizes transient hybridization of DNA oligonucleotides to identify the sequences of individual molecules within a randomized library. This is a strong and innovative manuscript that introduces new capabilities in single-molecule biophysics by directly linking equilibrium non-equilibrium dynamics to DNA sequence at the level of individual molecules. The technical execution is impressive, and the biological applications are compelling. I do not have any significant technical concerns, but there are a number of changes that could be made to make this complex work more compelling and clear to the reader.

Major comments
1. Although the introduction summarizes limitations of prior sequencing-linked single-molecule approaches (Refs 7-9, 10-11), the new capabilities introduced by SPIN-Seq could be more clearly articulated. For example, it is noted that other methods exhibit the shortcoming of “dependence on a solid support sub-optimal for single-molecule imaging”. It would be helpful to explain why this restricted the biological questions that could be investigated and how SPIN-Seq avoids these limitations.
2. While the results section clearly explains how 3–5 nt sequencing is achieved, the manuscript does not explicitly discuss the practical limits, costs, and trade-offs associated with further scaling of the method. It would strengthen the work to more clearly discuss the cumulative limitations imposed by multiple interrogation cycles (e.g., molecule loss, surface stability), such as whether mismatch discrimination degrades with increasing gap length or sequence degeneracy, and the expected trade-off between library size, and fidelity of quantifying the upstream kinetic measurements.
3. While the CAP binding and RNAP initiation examples convincingly demonstrate the power of the approach, both systems involve bacterial proteins acting on naked DNA substrates. It would strengthen the manuscript to briefly discuss potential challenges that may arise when extending SPIN-Seq beyond these demonstrated systems.
4. The approach by which promoter/RNAP traces were sorted into classes should be articulated in more detail. For example, what was the cutoff for “extensive cycling” that identified a molecule as Class II? Was the sorting done manually based on the HMM idealizations or were cutoffs defined and the sorting done automatically? Additionally, details of the HMM implementation (e.g., allowed number of states, initialization/constraints, and whether parameters were shared across sequences) should be briefly summarized in the main text or methods.
5. The sequence identification is based on the slow component of bi-exponential fits to seal dwell time distributions, but the fast component dominates (e.g. 70-80% weight in Fig. 2A-B). What does this fast component represent, and does it give any information that could either complicate or enhance analysis of binding?
6. A number of figure panels are too small to see clearly, such as the right-hand side of Fig. 1D and panels C and D of fig. 3. It is impossible to see the circles and squares in Fig. 2G. I do not think they are necessary here anyway, as fig. S9 is sufficient to drive home this point.

Minor comments
1. Several supplementary figures (e.g. S4-S5) contain key quantitative validation of the base-calling and analysis pipeline. The authors may consider briefly summarizing the main quantitative conclusions from these figures in the results section of

the main text to make the robustness of the method more immediately apparent to readers.
2. For clarity, the correspondence between the four transcriptional classes (Class I–IV) and the mechanistic states depicted in Fig. 5A could be made more explicit. For example, visual cues such as arrows or labels linking Class I–IV to Fig. 5A, a color-coded legend, or a simplified schematic adjacent to Fig. 5E would help guide the reader
3. The localization plots are not explained clearly when they are introduced in the text, only in the methods. It would be helpful to explain them when they are introduced, including specifying what is meant by a “localization”, and to make their panels large enough that axes can be labeled (e.g. “distance from molecule centroid” or whatever is appropriate).
4. Pg. 3 line 118: What is meant by “binds the gap well”? It doesn’t appear that there is anything special about the R-seals that make them bind better than other oligonucleotides with the same complementarity pattern.
5. Pg. 5 line 186: “two DNA strands were hybridized”. This makes it sound like two strands were hybridized to each other, rather than two additional strands being hybridized to the immobilized strand. The language should be clarified.
6. Pg. 7 line 276. I couldn’t follow what was meant by “...and a 2 A/U nt at the…”.
7. Y-axes are simply labeled “fluorescence” or “intensity” in a number of figure panels. It would be helpful to specify what the fluorescence is from. For example, in Fig. 4G the y-axis would be labeled “CAP fluorescence” and in 4H it would be labeled “R-seal fluorescence”.
8. It would be helpful to briefly explain in the main text the approach of obtaining a library of one strand of DNA and then extending the other with DNA polymerase in order to obtain a library with only base-paired sequences.
9. Fig. 5H-J and S25 show percentages, not fractions (as the captions suggest).
10. In the plots showing dwell time distributions and fits, the number of molecules analyzed should be provided (in the figure, caption, or a supplementary table).
11. Supplement pg. 3 line 81: The concentration of the glycogen stock should be provided.
12. Supplement pg. 9 line 349: Why is “all acceptor channels” plural? It would be more clear to identify the channels by their color, since the detected fluorophore is not acting as a FRET acceptor in all cases.
13. Fig. S3: The x-axis of panel B says “mFOLD server” while the caption says “DINAmelt server”.
14. Fig. S5A: It would be helpful if the definition of the y-axis of this plot was more specific.
15. Fig. S6B and a few other places. Many of these plots appear to have a slow component that is not being adequately fitted, even though it is stated that the fits are bi-exponential. What is the significance of this slow component, and is it problematic for it to not be fitted?
16. Fig. S6-S7, S10-12, S14-S15: It would be helpful to state in the captions what concentration was ultimately selected for the experiments in the main text and why.
17. Fig. S10C caption: The plots are left and right, not top and bottom.
18. Fig. S12: Panel D is not labeled in the figure.
19. Fig. S17: The cyan tint of the “no sequence” bars is barely visible. A brighter color or thicker lines would be more clear.
20. Fig. S21B: The meaning of the red line is not specified, and it is difficult to see.
21. Fig. S21A: The plots and their sequence labels are too small to see clearly. It would be helpful to indicate on the figure what of the categories described in the caption each sequence was assigned to.
22. Methods lines 145 and 152: It would be more clear if consistent units were used (mm2 vs µm2).

(Remarks on code availability)

Reviewer #4

(Remarks to the Author)
I co-reviewed this manuscript with one of the reviewers who provided the listed reports. This is part of the Nature Communications initiative to facilitate training in peer review and to provide appropriate recognition for Early Career Researchers who co-review manuscripts.

(Remarks on code availability)
I did not independently install or execute the analysis code. The manuscript provides links to in-house software used for localization, HMM fitting, and base calling, and the described workflows appear consistent with the reported results.

Version 1:

Reviewer comments:

Reviewer #1

(Remarks to the Author)
This revised manuscript (NCOMMS-25-94084-T, “Unraveling single-molecule reactions via multiplexed in-situ DNA sequencing”) submitted by Hazra et al. has addressed most of the previous comments. However, several issues should be clarified for publication.

1. Regarding the interpretation of double-exponential decay in the lifetime distributions, the authors showed that this behavior arises not only from base-pairing during hybridization, but also from stacking effects at the gap. This is very important mechanistic information. Even if the authors do not add revised data, they should explicitly mention in this manuscript that the heterogeneous behavior originates from the stacking effects at the gap.

2. In Fig. S6B, the authors stated that average dwell times represent the expected lifetime of randomly chosen hybridization events. However, in the interpretation of the data in Fig. 2A and 2B, they used t2. The same criterion (average dwell times) should be applied consistently. Alternatively, the authors should clearly explain why different lifetime criteria were used for different datasets.
3. The rationale for selecting 100nM/300nM as the optimal condition is not sufficiently clear. In Fig. S7, the dwell time and location graphs must include the corresponding complementary data for clear comparison.
4. Regarding the R-seal/U-seal optimization at 0.5 uM R-seal, the authors claimed that 1.5 uM U-seal is optimal. However, this conclusion is not clearly supported by the current presentation. To make this conclusion more quantitative, the authors should add and additional graph showing the ratio between complementary and 1-nt mismatch U-seals. The ratio can be easily calculated from the data in Fig. S11 and S12.
5. For “G” in the gap in Fig 2F, the dwell times for “G,” “A,” and “T” are overall high and are not dramatically distinguished from that for “C.” In addition, this trend does not appear to be reflected in Fig. 2E and Fig. S1. The authors should explain this.
6. In the analysis, 2,000–2,500 molecules were detected in a single FOV, but only 300–600 of them were actually used for sequencing. The authors need to explain why the remaining 75–85% of molecules were excluded from the analysis.

(Remarks on code availability)

Reviewer #2

(Remarks to the Author)
The authors have sufficiently addressed our concerns in the revised manuscript.

Minor comment: We noted that the figure legend for Figure 2 is missing panel D (likely a typo for C,D).

(Remarks on code availability)

Reviewer #3

(Remarks to the Author)
The authors have significantly improved the clarity of the manuscript in this revision, as well as adding details important for replication of the study and assessment of the method’s robustness. My only remaining questions concern major comment 4 from the original review, which was about assignment of traces in the transcription initiation experiment. While the authors added a lot of useful information in response to this comment, the provided details raise a few further questions/comments.

1. The authors state that “Emission means were initialized near the peaks of the empirical FRET efficiency distribution”, but the FRET efficiency distribution is not shown anywhere. A histogram showing the distribution these choices were based on should be shown.
2. By stating that emission means were “initialized” at empirical values (presumably the EFRET = 0.2, 0.38, 0.5 and 0.6 values mentioned later in the paragraph, which should be clarified), the authors imply that these values were allowed to float during idealization. From the authors’ statement that all trajectories were idealized “with shared emission and transition parameters”, it is not clear whether only the initial guesses were identical for all traces, or whether the emission means were kept identical across all traces but allowed to float relative to their initialized values (e.g. the algorithm could find that an initial guess of 0.38 is better modeled as 0.41, giving all molecules an idealized value of 0.41 when their traces dwell at FRET efficiencies in that ballpark). These details should be made clear.
3. What fraction of the traces were ambiguous, and how were they handled? This is particularly important if EFRET values were allowed to float individually for traces, as some traces may have ended up with states at FRET efficiencies that were ambiguous relative to the initial model, such as ~0.45.
4. The authors state that the state number was “validated for robustness”. They should briefly describe what this validation entailed.
5. The second paragraph describing HMM (“Following HMM idealization…”) states that Class III molecules showed transitions from 0.2 to 0.4, but 0.4 is not listed as one of the FRET efficiencies used to initialize the model. Is this a typo, or does it refer to the 0.38 state? If emission means were allowed to float for molecules individually during HMM, can an EFRET ~0.4 be reliably distinguished from EFRET ~ 0.38?

(Remarks on code availability)

Reviewer #4

(Remarks to the Author)
I co-reviewed this manuscript with one of the reviewers who provided the listed reports. This is part of the Nature Communications initiative to facilitate training in peer review and to provide appropriate recognition for Early Career Researchers who co-review manuscripts.

(Remarks on code availability)

I did not independently install or execute the analysis code. The manuscript provides links to in-house software used for localization, HMM fitting, and base calling, and the described workflows appear consistent with the reported results.

Version 2:

Reviewer comments:

Reviewer #1

(Remarks to the Author)
Dear editor,

all issues that I raised are addressed and figured out. This manuscript is ready to publish in Nature Communications.

(Remarks on code availability)

Reviewer #3

(Remarks to the Author)
The revised supplementary information provides methodological clarity regarding HMM initialization, global fitting strategy, ambiguous trace handling, and state-number validation. The transcription initiation analysis has been substantially improved by these additions. I offer just two aesthetic suggestions for the new SI text: (1) Cite the previous work in the manner used elsewhere in the supplement rather than including its full citation in the text, and (2) subscript "FRET" in E_{FRET} as done in the main text.

(Remarks on code availability)

Reviewer #4

(Remarks to the Author)
I co-reviewed this manuscript with one of the reviewers who provided the listed reports. This is part of the Nature Communications initiative to facilitate training in peer review and to provide appropriate recognition for Early Career Researchers who co-review manuscripts.

(Remarks on code availability)
I did not independently review or execute the supporting code as part of this assessment.

Open Access This Peer Review File is licensed under a Creative Commons Attribution 4.0 International License, which permits use, sharing, adaptation, distribution and reproduction in any medium or format, as long as you give appropriate credit to the original author(s) and the source, provide a link to the Creative Commons license, and indicate if changes were made.

In cases where reviewers are anonymous, credit should be given to 'Anonymous Referee' and the source.
The images or other third party material in this Peer Review File are included in the article's Creative Commons license, unless indicated otherwise in a credit line to the material. If material is not included in the article's Creative Commons license and your intended use is not permitted by statutory regulation or exceeds the permitted use, you will need to obtain permission directly from the copyright holder.
To view a copy of this license, visit https://creativecommons.org/licenses/by/4.0/

Note to all reviewers: our responses are in light blue, and any changes in the text are shown as a gray-highlighted text.

Reviewer #1: This manuscript (NCOMMS-25-94084-T, "Unraveling single-molecule reactions via multiplexed in-situ DNA sequencing") submitted by Hazra et al. introduces SPIN-Seq (Single-molecule Phenotyping and In-situ Sequencing), a novel single-molecule platform that directly links the functional phenotypes of individual DNA molecules to their underlying sequences on the same surface and within the same microscope field of view. The authors develop a transient hybridization-based in situ sequencing strategy that enables accurate reading of up to five nucleotides per molecule and apply this technique to investigate how DNA sequence variations influence transcription factor binding and transcription initiation dynamics by E coli RNA polymerase. This work integrates substantial methodological innovation with biologically meaningful insights into how DNA sequence information is translated into functional phenotypes in protein-DNA interactions. However, to strengthen this manuscript and improve clarity, additional experiments are required, and several protocols need to be described in greater detail. In particular, the optimization procedures are insufficiently explained. After appropriate revision, this manuscript could be suitable for publication in Nature Communications. Major and minor comments are detailed below.

Major comments

1. Overall, many figures are too small to read clearly. The authors should provide higher-resolution data images and enlarge axis labels and annotations throughout the manuscript.

We agree. We increased the number of figs from 5 to 8 to allow more space for individuals panels, and enlarged axis labels and annotations, which has substantially improved clarity.

2. Several dwell time distributions exhibit double-exponential decay. The authors should explain the origin of the double-exponential decay in dwell times and clarify the meanings of the short (t1) and long (t2) dwell time components. This explanation is important for understanding why t2 was chosen for comparing the seal-binding dwell times.

This is an important point, and we thank the reviewer for raising it. We note, however, that **we only used t2 to compare seal-binding times in the initial proof-of-concept experiments** in Figs. 2A and 2B. In fact, both t1 and t2 lifetimes decrease as we move from a complementary to a 1-nt mismatch seal.

After the proof-of-concept experiments, we modified our conditions to increase sampling of bound states (Fig. 2C); for the dwell-time analysis of the complementary and 1-nt mismatch gap-seal binding in Fig. S2, all dwell times were fitted to a single exponential decay (for consistency). We also note that our base-calling algorithm uses the localisation count (not lifetimes), and thus includes short-binding events.

Regardless, we agree that understanding the origin of double-exponential decays is important for understanding the hybridization mechanism in DNA gaps. However, this origin is complex, reflecting that DNA **hybridization in DNA gaps is a complex process** affected both by base-pairing and base-stacking at the seal ends. To explore the hybridization mechanism in gaps, we performed single-molecule binding-unbinding kinetics in DNA gaps (where seal binding is stabilised by stacking at both seal ends), on DNA overhangs (where seal binding is stabilised by stacking at one end), and on single-stranded DNA (with no seal binding stabilisation due to stacking). Our experiments use smFRET, helpful for capturing hybridization intermediates.

Seal binding to complementary **single-stranded DNA** showed a single dwell time of ~1 s (**Fig. R1**). In contrast, **heterogeneous behaviour emerges when seal binding is stabilised by stacking** either on one end or both ends. Seal binding to **DNA overhangs** revealed that some molecules consistently showed a mixture of short and long dwells (Category 1; lifetimes of ~3.5 s and ~10.3 s**; Fig R2**, left), whereas others consistently exhibit short dwell times (Category 2; ~0.8 s). Seal binding to **DNA gaps** also showed heterogeneous behaviour; as above, some gap molecules consistently show a mixture of short and long dwells (Category 1; ~1.9 s and 17.8 s; **Fig R3**, left), whereas others consistently exhibit shorter dwell times

(Category 2; 1 s and 4.2 s **Fig R3**, right). These results show that the introduction of base stacking is correlated with the onset of dwell-time heterogeneity, and with the increase in the duration of the long dwell-time component.

FIGURE REDACTED

The results above from part of ongoing work focusing on the mechanism of seal hybridization in DNA gaps, which we will discuss in a separate manuscript.

We changed the discussion of Fig 2A-2B to state that both lifetimes decrease as a mismatch is introduced. However, we prefer not to discuss the origin of the lifetimes in the present ms (and have not included the results shown here), since this will detract attention from the sequencing and functional aspects of the work, and will break the manuscript flow (especially since the origin is not as clear-cut); as we noted, knowledge of the exact lifetimes origins does not affect either our sequencing, or the connection of sequence to function.

FIGURE REDACTED

3. Fig. S6B represents dwell times of R-seals depending on U-seal concentrations. The authors should clearly state the conclusion drawn from this analysis. Specifically, what is the optimal concentration of U-seals based on the dwell time data and how was the optimal concentration determined?

The optimal U-seal concentration range was determined by comparing the dwell-time distribution and localization-count of R-seal binding in presence of varying concentrations of complementary and mismatch U-seals. Specifically, we kept R-seal at 100 nM, and titrated it with 100-600 nM complementary seal (Fig S6) or mismatch seal (Fig S7) to optimise the contrast between the binding of complementary and mismatch U-seals. As seen in Fig S6C-D, both the R-seal dwell time and the average number of localizations per molecule decrease substantially as we increase the concentration of complementary U-seal.

FIGURE REDACTED

If we compare the slope of the dwell-time vs U-seal concentration for complementary and mismatch seal, we see that the dwell-time decrease for mismatch U-seal is not as sharp as that of the complementary U-seal. A similar trend is seen for the average localization counts vs U-seal concentration plot. However, at higher concentrations of the mismatch U-seal (500-600 nM), R-seal binding diminishes. We thus concluded that the optimum U-seal concentration range is 3- to 4-fold higher than the R-seal concentration, and selected 300 nM U-seal and 100 nM R-seal for our 1-base sequencing.

The above comparison is already described in SI section “Optimisation of sequencing by competitive inhibition”; we edited the same SI section to clarify that this is a comparison for the **same** concentration range.

In addition, although the authors state that the dwell times were fitted with a double-exponential decay function in Fig. S6B, only the first distribution (top left) appears to be properly fitted. The remaining datasets should be refitted appropriately, and the values of t1, t2 and average dwell time should be displayed.

We had fitted most distributions with a mono-exponential decay since the long dwell times were much less abundant (y-axis: log scale). We agree, however, that this was inconsistent, and have now fitted all distributions using a bi-exponential model; we also revised Fig. S6B and Fig. S6C.

Along the same line, Fig. S6C appears to use average dwell times. The authors should explicitly define these values as average dwell times and explain why average dwell time was chosen instead of t1 or t2. This same clarification is required for Fig. S7C and S12C.

The bi-exponential dwell-time distribution reflects heterogeneity in seal dissociation due to base stacking at gap ends (see answer to Major comment 2). To compare the stability of different gap-seal pairs, we use the mean lifetime of the bound state, obtained from the first moment of the dwell-time distribution: $<\tau>= \int_0^\infty tP(t)dt = \frac{a_1\tau_1+a_2\tau_2}{a_1+a_2}$. This average dwell time represents the expected lifetime of a randomly chosen hybridization event, and provides a statistically well-defined and physically meaningful measure of overall duplex stability. This approach accounts for both kinetic components and their relative populations, rather than assuming a single dissociation rate. We added the formula and part of this explanation in the revised section on SI section on “Optimisation of sequencing by competitive inhibition”.

4. Regarding R-seal/U-seal optimization assays in Fig. S6 and S7, at 0.6 uM U-seal concentration, mismatched U-seals completely suppress R-seal binding in Fig. S7A, while complementary U-seals show similar R-seal binding in Fig. S6A. This result appears counterintuitive, as complementary U-seals would be expected to suppress R-seal binding more efficiently than mismatched U-seals. The authors should explain this observation.

As discussed in the SI, at high mismatch U-seal concentrations, R-seal binding is indeed affected. In addition, in this specific imaging round at 0.6 μM mismatched U-seal, the imaging quality was lower than the lower concentrations, and the bound states are not seen as clearly on the fluorescence time-trace, but they are being picked up by the localisation algorithm. Regardless, the binding pattern that emerges from the complementary and mis-matched U-seal concentration-dependence experiments (which were run on separate days and on different coverslips) clearly identified the 100nM/300nM R-seal/U-seal ratio as the optimal for the competitive assay. Notably, in our actual sequencing assay, the competition between R-seal and the different U-seals is examined on the *same* day, *same* coverslip, and same single molecule, minimising differences between experiments, and allowing reliable base-calling, e.g., see Fig. S8.

5. It is clever to use formamide to increase transient bindings of R-seals for long gaps. However, although optimization experiments are shown in Fig. S10, different formamide concentrations are used depending on gap size. The authors do not explain how the optimal formamide concentration was determined depending on gap size. The rationale underlying this choice should be described.

As we move to longer gaps, the length of the gap-seal hybrid increases, making seal binding less transient. This stability increase may lead to information loss (due to fluorophore bleaching while stably bound), to difficulties in discriminating between stably hybridized molecules and non-specifically surface-adsorbed molecules, and to the need for more extensive washing for seal removal for each interrogation.

Making binding more transient addresses these issues. In the case of a 13-nt gap, we destabilised the hybrid by increasing the formamide concentration; since too much destabilisation makes binding difficult to detect, we identified an optimum formamide concentration range (15-20%); using of these concentrations maximises the number of binding events while keeping the dwell time detectable with our current temporal resolution. We added similar text to the SI section on “Optimisation of sequencing by competitive inhibition”.

6. Fig. S12 appears to address U-seal concentration optimization for R-seal binding of 5-nt sequencing. However, the explanation is insufficient. The criteria for determining the optimal U-seal concentration are not described.

(In the SI of the submitted ms, S12 refers to 3-base sequencing, so we address the question on that basis.) The criteria for selecting the optimal U-seal concentration are the same as we stated in our response to Major comment #3. We added this short explanation in the paragraph explaining Fig. S11 and S12.

Moreover, the binding kinetics in Fig. S12A do not show a consistent trend: some traces show suppression of R-seal binding at high U-seal concentrations, while others do not. Does not the single mismatched U-seal suppress R-seals? Additionally, mismatched U-seals do not appear to alter dwell times across concentrations. The authors should clarify what conclusions are drawn from Fig. S12.

The gallery of traces was added to show the full range of behaviours and the temporal/spatial heterogeneity of the binding landscape. For up to 1.5 μM, the mismatch U-seal does not significantly affect R-seal binding for all molecules, showing a clear trend. At higher concentrations (which also correspond to the longest incubations), 2 out of 10 molecules (#4 and #8 from top to bottom) show some step change in their ability to respond to R-seal binding due to unknown reasons (e.g., due to loss of one of the flanking strands, or binding to surface, or chemical damage). Loss of ability to respond to R-seal binding in a sequence of interrogations affects the method throughput, but does not affect base-calling accuracy.

Re the minimal change in R-seal dwell times over a concentration of mismatched U-seal: the main effect that we expect from the competition between R-seal and U-seal binding is the decrease in the on-rate, which in turn decreases the number of binding events, and the number of detected R-seal localisations (as seen in Figs. S11D and S12D). In addition to this, we see that the presence of complementary U-seals also decreases the R-seal dwell times, which means that they actively displacing R-seals (we added text to that effect in the SI). In contrast, 1-nt mismatched U-seals are much less able to destabilise R-seal binding, hence the minimal change in R-seal dwell times across U-seal concentrations.

Re the conclusion from Fig. S12: this figure, along with Fig. S11, uses the number of U-seal localisations as a function of U-seal concentration to define the ratio of R-seal/U-seal concentration that should be used in the competitive inhibition assay for 3-nt sequencing.

7. The authors use purified CAP and E coli RNA polymerase. The activity of the purified proteins should be validated using additional biochemical assays, such as EMSA for CAP and in vitro transcription assay for E coli RNA polymerase.

We thank the reviewer for the suggestion. We measured the CAP DNA-binding activity using EMSA assays (**Fig. R4**); the activity was ~20%, within the range of 15-25% typically obtained for purified CAP (Kapanidis PNAS 2004; Kapanidis JMB 2001). Re RNAP, we used an RNAP preparation and conditions we have characterised extensively using *in vitro* transcription (Duchi…Kapanidis, Mol Cell 2016; Dulin…Kapanidis, Nat Comm 2018; Wang…Kapanidis, NAR 2025). We added this information to the sections of SI describing our protein preparations.

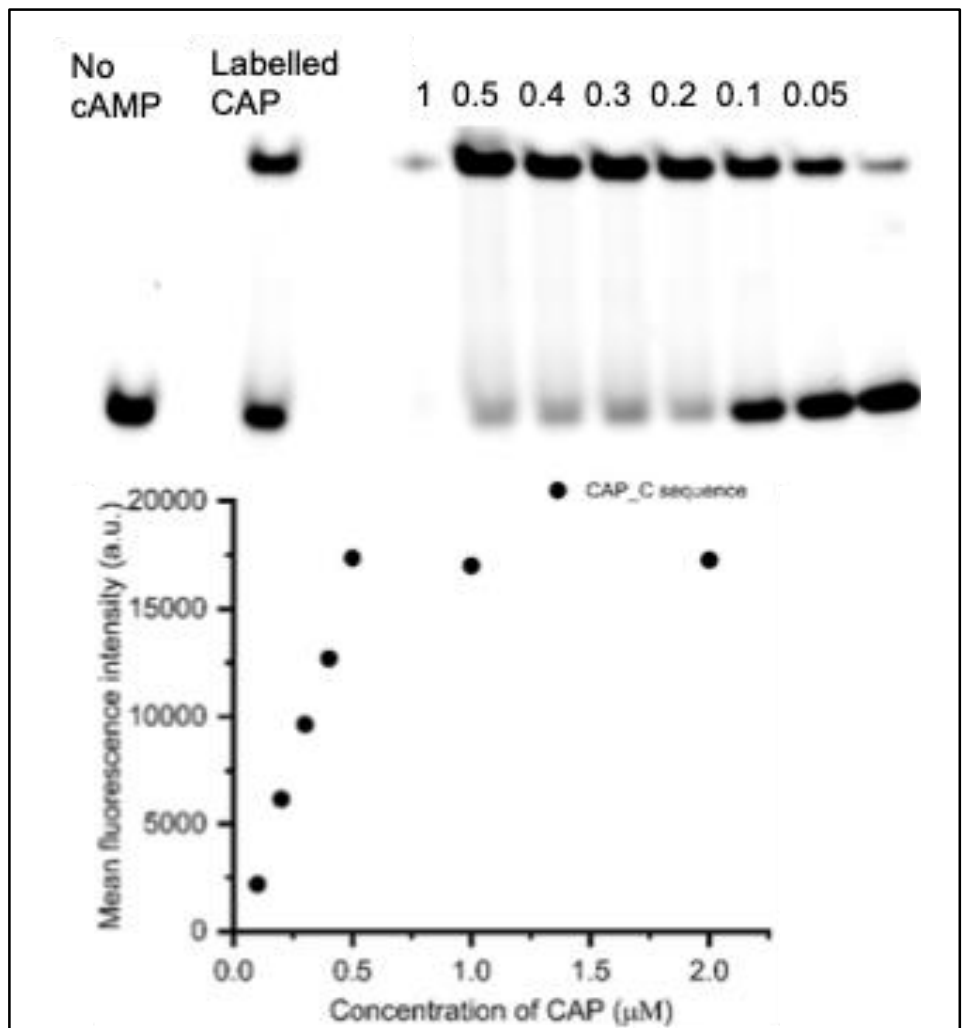

Fig. R4. **A**. Electrophoretic mobility shift assay (EMSA) for measuring DNA-binding activity for CAP. Assay was performed by adding 50-2000 nM CAP monomer to 50 nM CAPcons DNA in CAP-DNA binding buffer. Top bands: CAP-DNA complex; bottom bands: free DNA. **B.** Fluorescence intensity of CAP-DNA complex band as a function of CAP concentration.

8. Regarding the in-situ hybridization for a gap, the authors report ~70% efficiency for 100 nM flanking strands and >90% efficiency for 1 or 2 uM unlabeled flanking strands. However, the 70% efficiency is measured using only one labeled flanking strand. When both strands must be simultaneously hybridized, the actual gap-formation efficiency is likely lower than 70%. For more accurate quantification, both strands should be

labeled with different fluorophores and analyzed using three-color colocalization.

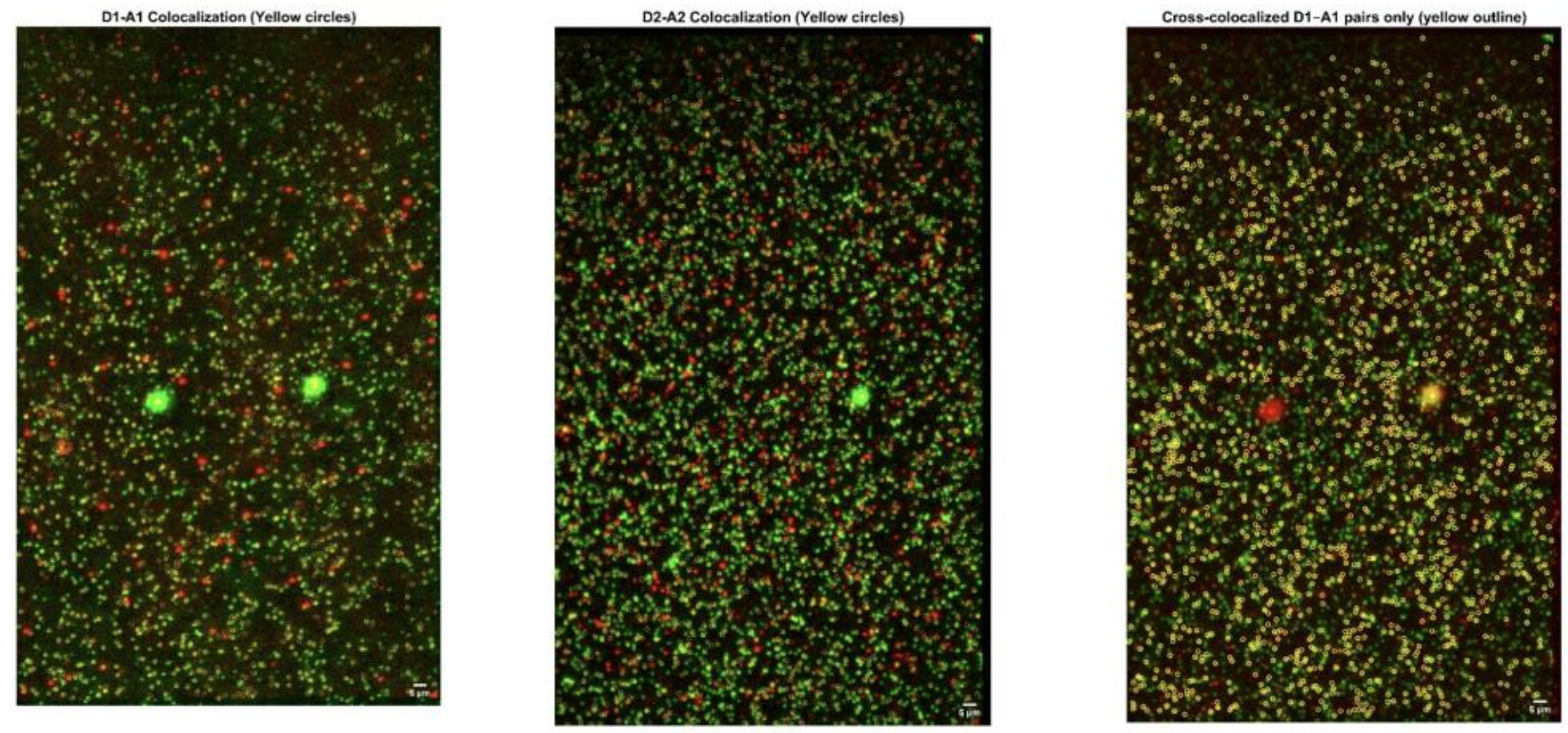

Fig. R5: Colocalization assay with two flanking strands. Left, merged channels of the gap-deposition image, we obtained the colocalized spots. Middle, merged channels of the reannealed gap formation. Right, cross colocalization between the colocalized spots of gap-deposited image and the reannealed colocalized spots.

As we stated in the original ms, "non-colocalised molecules in the in-situ formed gaps likely stem from non-specific binding of the Cy3B-modified flanking strand on the surface". To test this, we determined what fraction of flanking-strand pairs on a surface-immobilised DNA gap reforms after a cycle of flanking-strand removal and reannealing. For this, we first formed the gapped DNA in-situ by annealing the bottom DNA strand with the flanking-strand pair, with each flanking strand labeled with a spectrally distinct dye (Cy3B and Atto655); we then imaged the resulting hybrids using 532/640 nm excitation, and performed colocalization analysis. We then removed the flanking strands (by incubating with 20 mM NaOH for 30 s, followed by washing) and re-annealed the labelled flanking strands to the immobilised strand (incubating for 15 min with 100 nM of each strand in assay buffer with 10% dextran sulphate), followed by imaging. The co-localization between green and red channels was 72% for imaging round 1 (**Fig. R5, left**), and 68% for imaging round 2 (**Fig. R5, middle**). However, the cross-colocalization efficiency of gap molecules showing green-red colocalization in both imaging rounds was 94% (**Fig. R5, right**), even at 100 nM flanking strands; this value represents the true efficiency in forming the DNA gap in situ. The efficiency should approach 100% at the operational concentration of 1-2 μM flanking strands. We also note that, even with some immobilised DNA having only one flanking strand (e.g., 1-5%), our assay works well as evidenced by our high base-calling accuracies.

We added the text above and the cross-colocalisation panel in the SI section "In-situ DNA gap preparation and sequencing".

9. For clarity, the authors should provide a clearer description of how the scattered plots for localization maps were generated.

Reference localizations were first identified from a master Picasso .hdf5 file and used as coordinates of interest for region-specific super-resolution reconstruction. For each selected reference index, the corresponding x–y coordinates were extracted and defined as the center of a spatial neighbourhood. Raw single-molecule movies (.tif) were loaded using the one_channel_movie interface and localized using Picasso's least-squares fitting routine (lq_fitting) with GPU acceleration (when available) and a fitting window of 5 pixels. Previously processed localization files (.hdf5) were loaded directly without re-fitting. All analyses were restricted to a predefined rectangular region of interest to reduce computational load and exclude irrelevant image areas. The x and y coordinates of the localizations were compiled into a two-dimensional array, and a KD-Tree spatial index was constructed to enable efficient spatial queries. For each reference coordinate, localizations within a radius of 2 pixels were identified using a radius-based neighbor search (query_ball_point). The matched localizations were extracted while preserving associated metadata (e.g., frame number, photon counts, and localization precision) and exported as independent hdf5 files for each region and movie. The resulting per-region localization datasets were subsequently rendered using Picasso's visualization tools. For comparative analysis, localizations corresponding to different DNA sequences were plotted using distinct color channels to enable direct visual discrimination in the reconstructed super-resolution images.

This entire paragraph above was included in the “Image and time-series analysis” of the SI.

10. The quencher-dye seal strategy demonstrated in Fig. 3E-3H works particularly well for long gaps and significantly reduces background fluorescence. This approach is highly suitable for studying promoter escape by E coli RNA polymerase. These experiments convincingly demonstrate both sequence dependence (Fig. 5H) during transcription initiation and the robustness of both quencher-dye and fluorescent seal strategies.

We thank the reviewer for their positive assessment.

11. In Fig. 5, the authors claim that C rather than G at +7 from the TSS enhances the promoter escape. In E coli genes or promoters, is cytosine statistically enriched at the +7 position? A genome-wide analysis demonstrating a preference for C over G at this position would significantly strengthen the biological relevance of this finding.

We thank the reviewer for raising this point. The reviewer suggests that organisms may be geared towards “avoiding” pausing, and toward fast promoter escape and subsequent transcription. However, this is not necessarily the case; often, a process is regulated by being slow under a set of conditions and fast under a different set of conditions. Some genes may also be constitutively in a low-expression state since this will allow production of the required amount of RNA and proteins. Moreover, in the context of our work on the initiation pause element (IPE; Bauer…Kapanidis, to be resubmitted), we had performed a gene ontology analysis of genes whose primary promoters either tend to contain the IPE (i.e., show a positive fold-enrichment) or avoid the IPE (negative fold-enrichment) in *E. coli* (**Fig. R6A**) and *M. tuberculosis* (**Fig. R6B**). Our findings suggested a complex landscape, where genes involved in growth, cell wall/membrane synthesis, and metabolism tend to contain the IPE, and genes involved in responding to cellular stress avoid the IPE.

FIGURE REDACTED

Minor comments

1. In the purification of the E coli RNAP holoenzyme, the location (subunit) of the HIS tag should be specified.

Info was added (β’ subunit).

2. Exposure times differ across experiments (Fig. 2A-2B, Fig. 2C-2D, and Figs. 3-4). The authors should explain the rationale for these differences.

Fig 2A-2B used 100-ms since it was exploratory and wanted to capture faster events. Once the dwell-times at different conditions were established, it was clear that longer exposures could be used; we thus used exposures of 250 and 300ms for the interrogation steps in Figs 3-4.

3. Depending on the gap size, the imaging buffers vary. For clarity, the authors should provide a summary table listing buffer compositions used for each gap size.

We added a new table (Table S4) in the supplement.

4. In Fig. 3F, the labels "A, G, C, and T" on the x-axis are misaligned and should be corrected.

Thanks for catching this – now corrected.

5. High concentration of (80 mM or 150 mM) MgCl2 and 5~10% of dextran sulphate are used probably to enhance the binding of DNA. These are not usual buffer conditions for DNA hybridization. For readers, the authors need to explain the rationale for using such buffers.

Our assay relies on maximizing contrast between the binding of a gapped DNA to its complementary seal and the binding of the same gapped DNA to seals with 1-nt mismatch. To achieve this, we need both high sequence specificity and a high number of binding-unbinding cycles per interrogation cycle. High $MgCl_2$ concentrations increase DNA duplex stability (Owczarzy et al, Biochemistry **2008**, 47, 5336) and use of 5-10% dextran sulphate as a crowding agent accelerates DNA hybridization (Ku et al, Biochem Biophys Res Commun. **2004** 315, 30; Wahl et al, PNAS **1979**, 76, 3683).

We added such a note (along with references) to the section "Real-time in-situ sequencing assay" in the SI.

6. Through the manuscript, R-seal concentrations are unclear. The R-seal concentration represents the total concentration of all R-seals? For example, in Fig. 3E, 500 nM is used for 64 R-seals. Then does 500 nM represent the concentration per individual R-seal or the total concentration of all R-seals combined? This should be clarified explicitly.

The total concentration of R-seals is 500 nM, i.e., for an R-seal library with 64 individual R-seals, each sequence is represented at a concentration of 500/64 nM, i.e., ~8 nM.

7. At line 130 on page 4, the sentence "we tracked R-seal binding to the gap while it is sequentially added mixed with one of the four U-seals" is grammatically strange and should be rewritten for clarity.

Agreed. The sentence now reads "we sequentially added different R-seal/U-seal mixtures to the gap and tracked R-seal binding", which should be clearer.

Reviewer #2: In this manuscript, Hazra et al. describe a new method called Single-molecule Phenotyping and In-Situ Sequencing (SPIN-Seq). This method allows for measuring a functional output on a single DNA followed by DNA sequencing to correlate the sequence with function at a single-molecule level. They use this method to examine sequence-specific transcription factor binding behavior and sequence dependences of RNA polymerase open complex dynamics. This method is promising and has clear applications to many biological systems. In general, however, I felt that the method needs to be explained in more detail both in the details of the experiments and the analysis so that it can be replicated by other single-molecule labs. I have several questions to help clarify the method and suggestions for additions that would bolster some of their conclusions.

Major concerns/comments:

1. The authors report accuracy for specific sequences, but they do not discuss the robustness for differences in base pairing potential. How robust is the sequencing method relative to base-pairing potential and repetitive sequences? Would the authors show how well their method sequences A-rich or T-rich regions or sequences with low GC-content?

We expect that the assay to perform well for A/T rich sequences. Despite their lower stability, such sequences will still form stable hybrids with fully complementary seals (with stability increased by stacking at both ends of the gap), whereas the 1-nt mismatch will substantially decrease binding, regardless of the exact composition. For example, in the CAP study, 5 out of 7 known bases were A/T, and there was excellent discrimination between the least binding sequencing trace and the remaining 3; the functional results validated the entire approach.

To further test our ability to address an A/T-rich region, we constructed an 8-nt-gapped DNA where all gap bases were adenines (AAAAAAAA, or “8A”). To read the base at position 3 on the 8A DNA gap, we interrogated it using four seals, each having A, T, G or C at seal position 3, and T for the remaining bases. Using the conditions for non-competitive 1-base sequencing (i.e., assay buffer with 5% formamide and 10% dextran sulphate), we observed strong binding for the fully complementary seal (i.e., having T at seal position 3), and weak or no binding for the three 1-nt mismatched seals (**Fig. R7**). Using localisation counting and our base-calling algorithm, we identified the base A in position 3 in ~850 gap molecules with ~97% accuracy. These results clearly establish that we can identify AT-rich sequences with high accuracy.

We modified the main text with the text of the paragraph above (end of section of “Sequencing single bases via transient DNA hybridization”), and added the results as a SI figure.

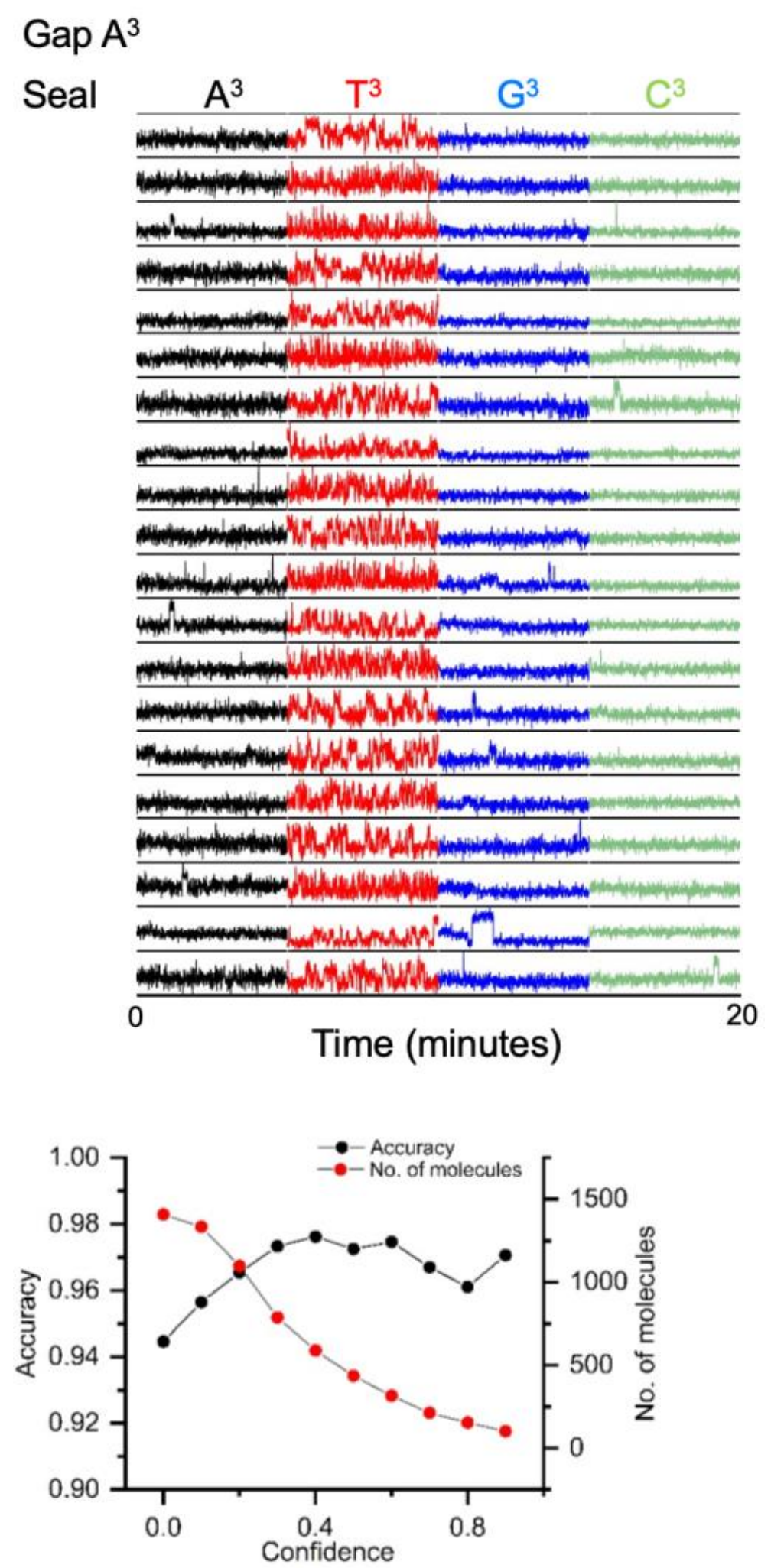

Fig. R7: Sequencing an 8-nt gapped DNA containing a stretch of 8 A bases. **A.** Representative traces obtained from four sequential interrogations using four seals possessing A, T, G and C at 3rd position and all T sequences at rest of the positions. We observed many more binding-unbinding cycles for seal T^3 (complementary seal) compared to the other three 1-nt mismatched. **B.** We performed localization counting and base calling for all gapped DNA molecules, showing ~97.3% accuracy at a confidence of 0.3, calling ~850 molecules.

2. Does SPIN-Seq work at higher temperature? How does the accuracy and robustness of the sequencing method change at higher temperature? I could imagine functional assays that require 37C, so it would be important to know if sequencing can be performed at higher temperature as well.

We had not explored sequencing at higher temperature, since the two parts of SPIN-Seq do not have to occur at the same temperature, i.e., the functional assay can be done at any temperature supported by the single-molecule microscope (e.g., 20-45˚C for the Nanoimager microscope we use), and then the sequencing is done close to room temperature (since the Nanoimager equilibrates at a temperature slightly higher than room temperature). For our RNA polymerase work, we performed both the transcription assay and the subsequent sequencing assay at 29˚C.

Following the reviewer's suggestion, we tested the ability and performance of gap-based sequencing at 37ºC using the A8 gap sequence used in our response their major point #1. The assay worked very well (**Fig. R8**), matching the performance at lower temperatures, with 777 out of 791 molecules being identified correctly (98.2% accuracy). We have not included the figure in the manuscript but we will include it in a future manuscript on the hybridization mechanism in DNA gaps.

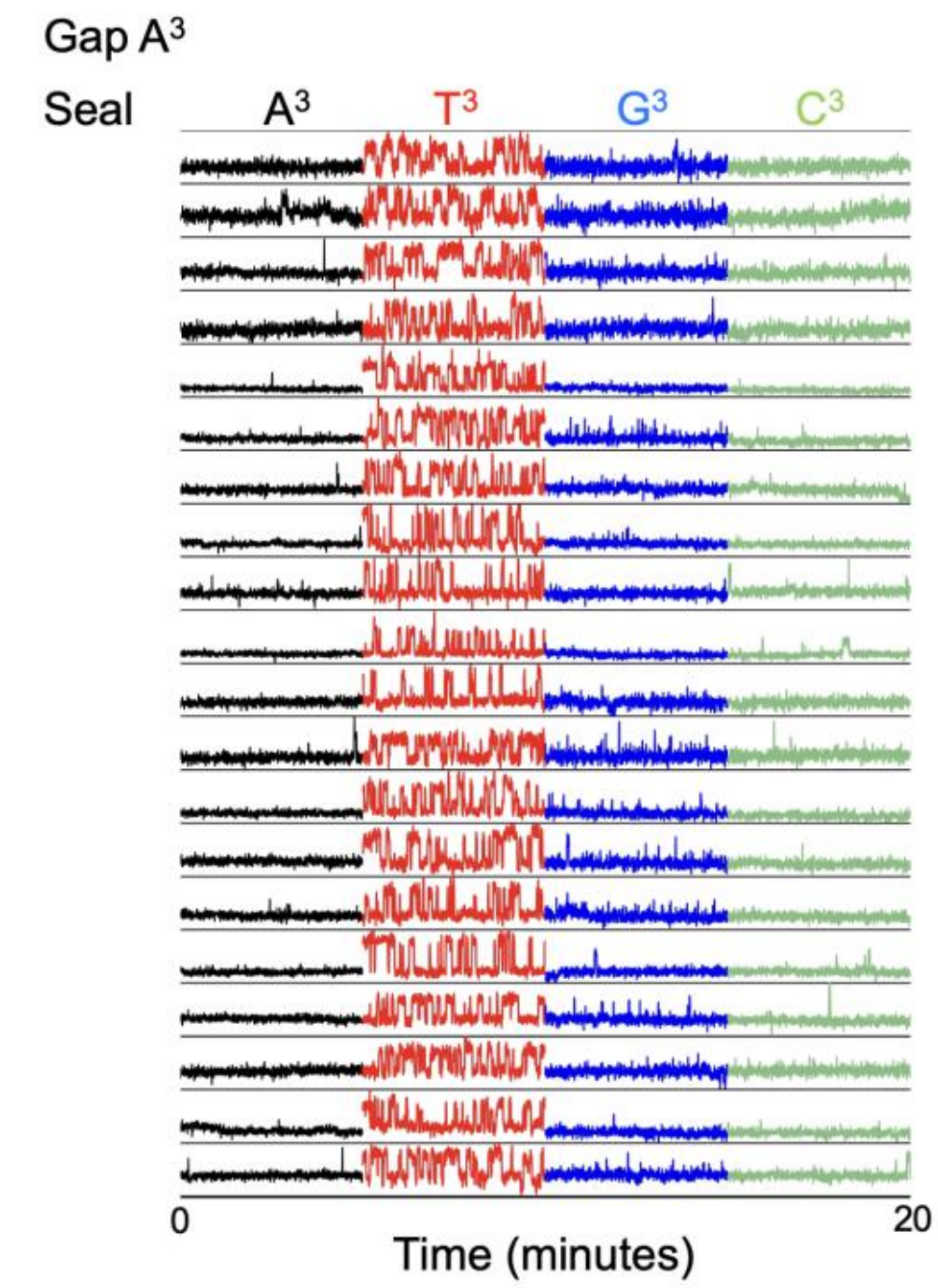

Fig. R8: Sequencing an 8-nt Gap-DNA at 37ºC. Representative traces obtained from sequential interrogations using four seals possessing A, T, G and C at 3rd position and all T sequences at all other positions. We observed many more binding-unbinding cycles for seal T^3 (complementary seal) compared to the three 1-nt mismatched seals.

3. The authors claim that this method can sequence 5 bases with high accuracy on the same surface (1024 possible sequences). What would the read depth be for each sequence? How many molecules can they image at once? Even with a large camera field of view around 10,000 molecules, the read depth per sequence would be ~10 molecules. Would the authors comment on this and how they would interpret the functional assay per sequence when the read depth is so low?

We agree that this is an important question. Currently, SPIN-Seq examines a single field-of-view (FOV) of 120x50 µm for functional activity and DNA sequence. Out of 2,000-2,500 detected molecules per FOV, we find that 300-600 molecules provide usable time-series for both function and sequence. For a 16-variant library, the assay takes ~1 hr/FOV (with ~40 min required for 2-nt sequencing; i.e., eight 5-min interrogations), offering a coverage of ~100 molecules per variant in 4 replicates (~4 hr microscopy time). This part of our work also showed that we do not necessarily need large sequence libraries to extract unique mechanistic information using SPIN-Seq.

To cover a library of 1024 sequences at read depth of 50 molecules per sequence (sufficient for identifying patterns of reactions and doing basic kinetic analysis), we will require ~50,000 usable molecules per library, which corresponds to 100-150 FOVs with our current throughput. Working with lower coverage (e.g., 10 molecules per sequence) is feasible with our current throughput, but can only provide information if the molecules are somehow pooled to see the importance of a base at a specific position but in a mixed context in terms of the neighbouring bases, which is not ideal.

To cover such libraries, we are currently working on extensions that increase throughput. Automated microfluidics will allow operation for extended periods, as shown for multiplexed methods (MUSCLE/SPARXS) that collected data for up to 7 days; the gap-interrogation step can also be shortened; e.g., 50% shortening of that step still maintains both high molecular throughput and high base-calling accuracies (>98%; see new Fig. **S26A**). Additional improvements include multi-colour versions to read bases in a single interrogation, and imaging larger FOVs (both currently in progress); note that earlier published microscopes were able to collect 15,000 molecules per FOV. In cases of cyclic reactions or transient protein binding, a unique way to increase throughput is by working at higher densities of gapped molecules, which can leverage resolving closely spaced molecules via high-precision localisation, already used in our analysis (**Fig. 2I; Fig. 4D**); we can currently image ~25,000 spatially resolved molecules in our 120x50 µm FOV. SPIN-Seq can also operate by sequencing only functionally important molecules of large libraries, e.g., by scanning the surface rapidly to identify the top 5-10 active molecules, sequence only these molecules, and use their features to further engineer a library.

We have added most of this response to the Discussion session.

Similar discussion of limitations and future extensions of SPIN-seq and related methods (MUSCLE/SPARXS) is also included in a recent *Science* review we have co-authored on multiplexed sequence-resolved single-molecule methods (Kapanidis et al, *Science* (2026) 391, 458). References to the review have been added.

4. In figure 2D, the authors show "a map of colocalizations around the interrogated gap DNA molecule". It is unclear that this is a single DNA molecule examined, so please indicate on the figure with an X or in some way to show where the immobilized gap DNA is located. What is the scale in XY? A localization grid would help show the magnitude of the localization. I cannot tell at this resolution what the scale bar says.

The map is indeed from is a single gapped DNA molecule, and we do have the XY position of the interrogated gap molecule (albeit only as a single Cy3B localisation after the deposition of gapped molecules; more localisations can be obtained if needed).

We have now generated localisation grids to help display the results more clearly in Fig panels 2D, 2F, and 3D of the original ms (note that figure numbers had changed), and added the single localisation for the interrogated gap molecule. We also improved the figure clarity and resolution.

5. For the competitive inhibition assay, would the authors comment on why they chose to label the R-seal and not the U-seal to probe when colocalization decreases as opposed to increases for detection?

The R-seal and U-seals exclude each other: when the R-seal binds to the gap, a U-seal cannot bind (and vice versa). We introduced the competitive inhibition version of the assay to have only <u>one</u> labelled strand per variant library, complemented by a larger set of unlabelled strands (which are easier to prepare and cheaper to buy). The absence of probes from U-seals also make their gap binding more stable, since adding fluorophores and quenchers to seals can decrease binding.

We added a clarification sentence to the main text in the section "Sequencing single bases using competitive inhibition of gap hybridization" (end of 1st paragraph).

<u>Minor comments:</u>

1. A discussion of the advantages, limitations, and comparison to other single-molecule based sequencing methods would be very useful in the conclusions section. For example, how long does the sequencing steps typically take? How does this change as more bases are sequenced? What materials are needed that are consumed in the experiment? Can probes be reused? What about cost?

We agree with the reviewer. To address their comment, we made a major revision and expansion of the Conclusions section (now named "Discussion"), where we cover advantages (mainly in the context of single-molecule based sequencing methods – see our response to <u>Reviewer 3, major comment 1</u>), and limitations (such as the time consideration and limited throughout, see our response to <u>Reviewer 2, major comment 3)</u>.

Re materials, the costs are fairly low for the competitive inhibition assay (which the one we used for our experiments with CAP and RNAP), since only one labelled seal strand is used for the sequencing; the rest of the seals are unlabelled and very inexpensive. For example, the total cost for the 16-variant library sequencing would be ~400 GBP. Moving to a 256-variant library (4-base sequencing) increases only the number of unlabelled seals, hence the price increases by less than 50 GBP; adding a quencher to the R-seal will add ~100 GBP. Costs can further be reduced by 50%, if needed, by doing the DNA modifications in house. Re reusing of the probes, we are working towards a universal probe set compatible with *any* sequence context in the functional assay.

2. The description of the base calling analysis in the supplement (starting at line 341) is hard to understand and needs clarification. Why are the authors fitting the first derivative of the PDF (line 353)? How is the threshold determined “algorithmically”?

We agree that the description needs clarification. The histogram of localization counts (LC) per molecule (as in **Fig. S5A**) is typically bimodal or skewed, consisting of a low-count population corresponding to background or weak-binding (as for mismatch seal binding in the non-competition experiment) and of a higher-count population due to repeated specific binding of the complementary seal. As we move from low LC values to high LC values, we encounter a transition point (i.e., an LC value) where the frequency stops decreasing fast, marking the transition from weak to strong binding, and defining an LC threshold that can differentiate between mismatch seal binding and complementary seal binding.

Rather than directly fitting the LC histogram (which is sensitive to binning and noise), we first compute the first derivative of the LC histogram to identify the transition point; the global minimum in the first derivative corresponds to the point of steepest decline between mismatch-dominated and complementary-dominated populations. We then fit the first derivative function with an exponential decay (Ae^{-Bx}) from its global minimum (x_{start}) onward, and use x_{start} + 3*B as the LC value where the histogram counts stabilize. At least one LC value should be higher than this threshold for the non-competitive version of the assay; further, at least three LC values should be higher than this threshold for the competitive inhibition version of the assay.

We merged the paragraphs above with our current text in the “Base calling” SI section. We also included a summary of our base-calling approach in the Main text (“Sequencing single bases via transient DNA hybridization” section).

What does it mean that “molecules show persistent localization clustering over time”? Why were localization bundle thresholds of 5 and 2 chosen for single or multiple base calling? What does it mean that the longest bundle should be smaller than 50% of the frame number? Please explain and clarify this section.

In true transient hybridization, binding events are temporarily distributed across many frames, reflecting repeated short-binding events. In contrast, “persistent clustering” refers to cases where many localizations occur within a continuous block of frames; such behaviour signifies non-specific binding of a seal to the surface, and can artificially inflate localization counts and bias base calling. To exclude these artifacts, we analyze the temporal distribution of localization frames for each molecule.

A “bundle” is defined as a sequence of consecutive-frame localizations, representing a contiguous temporal binding event. The bundle thresholds were chosen based on the different gap lengths used for single- and multiple-base calling experiments. For single-nucleotide identification, an 8-nt gap was employed, whereas for multiple-base sequencing a longer, 13-nt gap was used. Since the seal dwell time increases with duplex length, the 13-nt gap exhibits longer binding than the 8-nt gap. Longer dwells reduce the number of independent binding events observed within a fixed acquisition window due to increased site occupancy.

In the 8-nt (single-base) experiments, we observed a relatively high number of transient binding events per molecule, allowing us to apply a more stringent requirement of at least five independent bundles to ensure robust base discrimination. In contrast, for the 13-nt (multiple-base) experiments, seal-binding events were longer, and therefore fewer in number; accordingly, a minimum of two independent bundles was sufficient to confirm transient behavior while preserving adequate molecule yield. We empirically tested higher bundle thresholds and found that increasing the requirement did not significantly improve base-calling accuracy, but instead substantially reduced the number of analyzable molecules. Therefore, the selected thresholds represent a balance between statistical robustness and data retention.

For each molecule we compute the duration (in frames) of each contiguous localization run and the longest such run. If the longest bundle occupies more that 50% of the total movie frames, it likely corresponds to a surface-adsorbed fluorophore (or labelled seal) rather than a transiently hybridized seal in our experimental

protocol. The 50% cut-off was chosen as a conservative threshold to exclude persistent artifacts while retaining molecules exhibiting repeated transient binding.

We added segments of these responses in the “Base Calling” section of the SI.

3. For the CAP-binding assay analysis, the authors state that “Traces were then fitted using a cluster-based approach for dwell-time analysis”. What is a cluster-based approach? Has this been used previously? Please explain.

We thank the reviewer for catching this error. Although we performed the trace analysis both using an HMM approach (by using hmmlearn and diagonal covariance fitting approach with two-state detection method) as well as a cluster-based approach, we finally kept only the HMM fitting procedure incorporated in the traceanalysis software. We have defined minimum number of frames of 5 to be considered as binding.

We have corrected our description in the Methods section accordingly.

4. Do the authors perform each sequential step by hand or do they use a pump system or some kind of microfluidics? If doing the washes by hand, are the traces spliced together on the same time axis with the washing steps removed? Would the authors comment on the any decrease in the density of molecules in a field of view during sequential washing and sequencing steps due to drift or photobleaching?

Although the assay is amenable to use of microfluidics, the results presented were collected without microfluidics. Solutions (seals, wash buffers) were added manually. The traces are spliced together on the same axis, using the movie-collection time rather the experimental time. As discussed, drift is corrected using the presence of fiducial marks; further, there is no influence of photobleaching per se since the seals exchange continuously, and thus are robust to photobleaching.

However, prolonged exposure of our surfaces to light can slightly decrease the number of molecules that continue to respond to transient binding, a known issue in DNA-PAINT (doi: 10.3390/molecules23123165).

To quantify the loss of molecules during the interrogation (due to washing, photoinduced damage or other reasons), we assessed the loss of binding capability of DNA molecules over repeated interrogations (**Fig. R9**). Overall, the vast majority (~97%) of molecules retained their binding capability for all eight interrogation cycles (40min), showing that our assay is robust to a large number of seal interrogations.

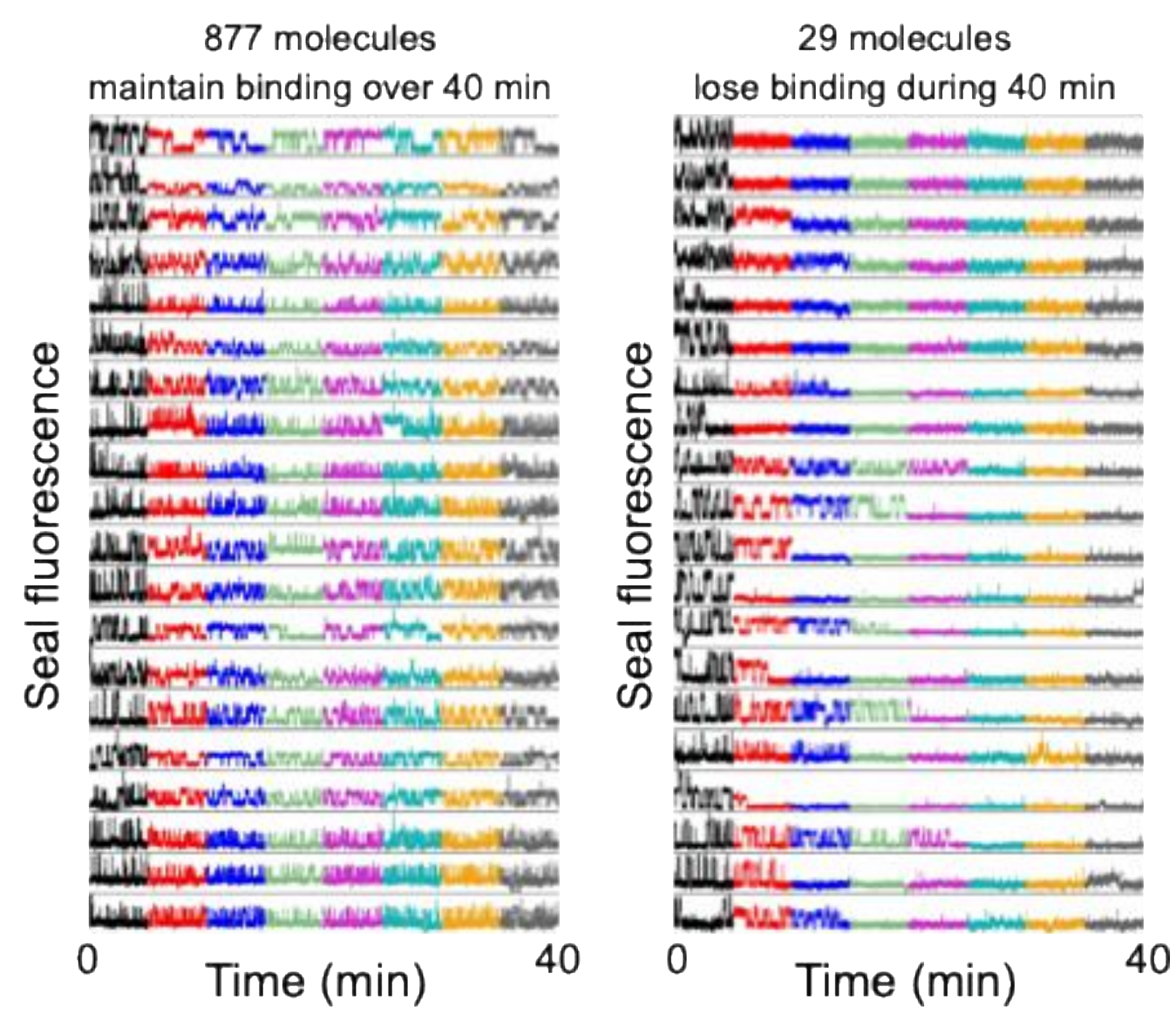

Fig. R9: Binding capability of DNA molecules over repeated interrogations. Gap-A^3 molecules were probed using an ATTO647N-labeled complementary strand (seal T^3) across eight interrogation cycles, each lasting 5 min, followed by a washing step; 877 molecules (~97%) exhibited characteristic binding–unbinding events across the interrogation traces (left panel), while 29 molecules (~3%) lost the binding–unbinding signature during one of the interrogation cycles (right panel).

We commented on the loss of molecules at long timescales in our Discussion, and included the figure in the SI.

5. In general, all of the figures have parts that are very small and hard to see. The text in some figures is almost illegible. Please increase sizes and resolution so the reader can inspect them easily.

We increase the number of figs from 5 to 8 to allow more space for the individuals panels, and thus allow enlargement of axis labels and annotations, which has substantially improved clarity.

6. Do the authors typically use multiple fields of view to generate enough data for sequence depth? Was a single field of view used in the non-equilibrium experiments to generate the kinetic data in Figure 5F or was the data aggregated from multiple movies? Please explain.

For the development of the sequencing assays, a single field of view per experiment was sufficient. For the non-equilibrium experiments with RNA polymerase, time-traces were aggregated from 24 separate movies (collected from independent experiments), which gave 1948 transcriptionally active molecules. Out of the 1710 transcriptionally active molecules that gave a useable sequencing read, 1447 were used for the pause-time analysis, and 263 were stably scrunched. We added this information in the SI section *Data analysis for transcription assay and in-situ sequencing*.

Typos/Clarity:

1. In the supplement, the immobilization density is reported as 0.2 molecules per mm2 but later in the text it says 0.2 per um2. (Lines 145-152) Which is correct?

The correct density is 0.2 molecules per μm^2; the typo it is now fixed.

2. The authors report TIRF at an angle of 56° (Line 170), but the glass-water critical angle is 63°. Would the authors clarify?

TIRF excitation was achieved using an objective-based configuration corresponding to a nominal setting of ~56° for the TIRF-setting lens on the Nanoimager, an angle different from that actually achieved at the glass-water interface. The nominal TIRF-setting lens angle of 56° was chosen after maximizing the S/N for the single-molecule measurement on the specific Nanoimager; notably, different Nanoimager versions have a slightly different setting for achieving TIRF excitation. We clarified this in the SI text.

3. Line 334-339: What is the background subtraction referring to? What image registration is being performed?

We thank the reviewer for catching this. These sentences were there by mistake, and have been removed.

4. The authors should state that they use a “kernel density estimation” to acquire the probability density function in the supplement (Line 352).

We have now incorporated this.

Reviewer #3: In this work, Hazra et al. demonstrate a new approach to multiplexed single-molecule imaging that utilizes transient hybridization of DNA oligonucleotides to identify the sequences of individual molecules within a randomized library. This is a strong and innovative manuscript that introduces new capabilities in single-molecule biophysics by directly linking equilibrium non-equilibrium dynamics to DNA sequence at the level of individual molecules. The technical execution is impressive, and the biological applications are compelling. I do not have any significant technical concerns, but there are a number of changes that could be made to make this complex work more compelling and clear to the reader.

Major comments

1. Although the introduction summarizes limitations of prior sequencing-linked single-molecule approaches (Refs 7-9, 10-11), the new capabilities introduced by SPIN-Seq could be more clearly articulated. For example, it is noted that other methods exhibit the shortcoming of “dependence on a solid support sub-optimal for single-molecule imaging”. It would be helpful to explain why this restricted the biological questions that could be investigated and how SPIN-Seq avoids these limitations.

We thank the reviewer for raising this. The single-molecule methods we note (MUSCLE/SPARXS) use MiSeq Illumina chips both for the functional assay and the sequencing, with the latter being done on Illumina sequencers after amplification of single DNA molecules.

A major issue with the MiSeq solid supports is their poor compatibility with labelled proteins, which tend to bind non-specifically to the solid support. As a result, single-molecule assays that rely on singly- or doubly-labelled proteins are difficult to perform on MiSeq (and, to our knowledge, none has been reported). SPIN-Seq, on the other hand, is fully compatible with labelled proteins, as shown in our ms (use of labelled CAP).

Since MiSeq surfaces differ from standard glass, adopters of MUSCLE/SPARXS may have to re-optimise their surface-immobilisation and functional assays (even for unlabelled proteins) for the MiSeq surface, a step that is not straightforward, especially for studies using complex protein systems; such optimisation sometimes takes years to complete. SPIN-Seq just uses standard glass surfaces and existing conditions and protocols for functional assays.

The dependence on a commercial platform also creates uncertainty about the actual composition of the solid support, and creates an uncertain supply chain; notably, Illumina has announced that MiSeq and its consumables are now obsolete (https://www.illumina.com/systems/sequencing-platforms/miseq/applications.html - although there will be support until 2029). It is unclear whether the miSeq replacement will support MUSCLE/SPARXS to the same degree. No such vulnerabilities exist for SPIN-Seq.

The difficulty in working with complex biological systems on MiSeq chips may also reduce the number of functional molecules/complexes per FOV, reducing the throughput of MUSCLE/SPARXS for non-equilibrium reactions or real-time interactions involving proteins. So far, no such studies have been published for MUSCLE/SPARXS. We also note that the throughput of MUSCLE/SPARXS for non-equilibrium studies (even if the system is fully functional on MiSeq chips) will be much lower than the impressive throughput they demonstrated for dynamics, since all non-equilibrium observations need to come from a single FOV.

We have added a brief mention of the low compatibility with labelled proteins in the Introduction, and a summary of SPIN-Seq advantages in the Discussion.

2. While the results section clearly explains how 3–5 nt sequencing is achieved, the manuscript does not explicitly discuss the practical limits, costs, and trade-offs associated with further scaling of the method. It would strengthen the work to more clearly discuss the cumulative limitations imposed by multiple interrogation cycles (e.g., molecule loss, surface stability), such as whether mismatch discrimination degrades with increasing gap length or sequence degeneracy, and the expected trade-off between library size, and fidelity of quantifying the upstream kinetic measurements.

We agree with the reviewer and we made a major revision and expansion of the Conclusions section (now named "Discussion"), where we cover limitations and practicalities. Regarding the specific points raised:

- *cumulative limitations imposed by multiple interrogation cycles*. Most time in the assay is taken by the *in situ* sequencing, with ~20 min required for interrogating a single position (when means ~100 min of interrogation time for a 5-nt sequencing). We have not experienced issues with deterioration of surface quality; however, we have experienced a small loss of responsive molecules after many cycles of imaging (a known issue in DNA-PAINT; see doi: 10.3390/molecules23123165), which reduces our throughput. Shortening the interrogation (see our response to Rev2, major point 3) should largely address this issue.

- *whether mismatch discrimination degrades with increasing gap length or sequence degeneracy*: our results show that we maintain a high base-calling accuracy (~97%) for all positions within a 5-nt sequence. Further, we have new results on an all-A sequence (see response to Rev2, major point 1), showing high base-calling accuracy with a weaker hybrid. As a result, we are confident that we have the base-calling accuracy needed to address a 5-nt library when our throughput becomes high enough to cover it. While there may be room and scope to go to even larger libraries, we believe that there is already a very large number of questions and systems that can be addressed by libraries of 4^5 members or less, so that additional increase is not necessary for the impact of the method.

- *expected trade-off between library size, and fidelity of quantifying the upstream kinetic measurements:* we have covered this point our response to Rev2, major point 3.

We have included part of these responses to the revised Discussion section.

3. While the CAP binding and RNAP initiation examples convincingly demonstrate the power of the approach, both systems involve bacterial proteins acting on naked DNA substrates. It would strengthen the manuscript to briefly discuss potential challenges that may arise when extending SPIN-Seq beyond these demonstrated systems.

Although the two systems studied are involved in bacterial transcription, the method is fully compatible with a much wider repertoire of systems and interactions. These include other protein interactions with nucleic acids (e.g., binding of eukaryotic DNA-binding proteins on nucleosomal DNA templates), interaction of nucleic-acid aptamers with their targets, and interactions of RNAs with RNA-binding proteins.

The prerequisites are to be able to prepare a variant library compatible with the functional assay, and to form a DNA gap around the variable region after the functional assay is complete. In case that the variant sequence is processed chemically (e.g., due to protein-driven DNA cleavage or DNA modification, and thus cannot be used for gap-based sequencing), then sequencing can be performed first, followed by formation of the reaction substrate on the surface, followed by the local chemical reaction.

Extensions of SPIN-seq can also analyse epigenetic modifications, e.g., by bar-coding different DNAs with different sequences and modifications to create a coded variant library, performing the functional assay, and then decoding the individual molecules to connect sequence and modification to function. In general, the use of bar-coding will enable exploration of sequence complexity much beyond what is available by randomizing short DNA regions.

In summary, there is a large number of systems immediately amenable to the method, with SPIN-seq extending the approach to provide a general framework to connect function to DNA sequence and other DNA properties than can be coded.

We added most of these comments in the revised Discussion.

4. The approach by which promoter/RNAP traces were sorted into classes should be articulated in more detail. For example, what was the cutoff for "extensive cycling" that identified a molecule as Class II? Was the sorting done manually based on the HMM idealizations or were cutoffs defined and the sorting done

automatically? Additionally, details of the HMM implementation (e.g., allowed number of states, initialization/constraints, and whether parameters were shared across sequences) should be briefly summarized in the main text or methods.

Single-molecule FRET trajectories were idealized using a hidden Markov model (HMM) hmmlearn with diagonal-covariance Gaussian emissions. A single HMM was trained on all trajectories, with shared emission and transition parameters and learned from the full dataset. Emission means were initialized near the peaks of the empirical FRET efficiency distribution, and transition probabilities were initialized uniformly. We kept minimum state length of 2 frames or 0.4 s. The state number was chosen based on biologically interpretable FRET levels and validated for robustness, since automatic state-selection methods frequently overfit noise in single-molecule FRET trajectories. We fixed the number of states to four based on known FRET levels observed in the data (E*~ 0.2, 0.38, 0.5, and 0.6).

Following HMM idealization, individual trajectories were classified manually based on quantitative features extracted from the idealized state sequence. Overall, we found four distinct classes of traces. Class I molecules give traces where E* starts from ~0.2 and reaches ~0.6. Class II molecules give traces where E* cycles between ~0.2 and ~0.38 values; these molecules can have high or low frequency cycling, but we didn't further separate them into different sub-classes; we required at least two cycles during the trace in order to place a molecule in this Class. Class III molecules give traces where E* shows a transition from ~0.2 to ~0.4 and then returns to ~0.2 without exhibiting further transitions. Class IV molecules give traces that exhibit a transition from ~0.2 to ~0.38 without returning to ~0.2 or showing any other transition during the time of data-acquirement (or before photobleaching).

The text above was added to the Methods (section on "Data analysis for transcription assay and in-situ sequencing").

5. The sequence identification is based on the slow component of bi-exponential fits to seal dwell time distributions, but the fast component dominates (e.g. 70-80% weight in Fig. 2A-B). What does this fast component represent, and does it give any information that could either complicate or enhance analysis of binding?

Please see response to Major Point 2 of Rev1. We present results that show that the introduction of base stacking is correlated with the onset of dwell-time heterogeneity, and the increase in the duration of the long dwell-time component. The mechanistic landscape is complex, however, and we will be addressing it in a separate ms.

6. A number of figure panels are too small to see clearly, such as the right-hand side of Fig. 1D and panels C and D of fig. 3. It is impossible to see the circles and squares in Fig. 2G. I do not think they are necessary here anyway, as fig. S9 is sufficient to drive home this point.

We agree. We increased the number of figs from 5 to 8 to allow more space for individuals panels, and to enlarge axis labels and annotations, which has substantially improved clarity.

<u>Minor comments</u>

1. Several supplementary figures (e.g. S4-S5) contain key quantitative validation of the base-calling and analysis pipeline. The authors may consider briefly summarizing the main quantitative conclusions from these figures in the results section of the main text to make the robustness of the method more immediately apparent to readers.

This is a good suggestion. We thus expanded the discussion of Figs 2D, S4 and S5 in the Results section by briefly summarising the use of localisation count and related algorithms for base-calling, as well as the mapping of localisations.

2. For clarity, the correspondence between the four transcriptional classes (Class I–IV) and the mechanistic states depicted in Fig. 5A could be made more explicit. For example, visual cues such as arrows or labels linking Class I–IV to Fig. 5A, a color-coded legend, or a simplified schematic adjacent to Fig. 5E would help guide the reader

This is an excellent suggestion. In the restructured Fig. 5, we introduced simple schematics that summarise the main reaction paths visualised as Class I-IV behaviors, and annotated the traces of original Fig. 5E with them. We used the same schematics for the propensity heat maps.

3. The localization plots are not explained clearly when they are introduced in the text, only in the methods. It would be helpful to explain them when they are introduced, including specifying what is meant by a “localization”, and to make their panels large enough that axes can be labeled (e.g. “distance from molecule centroid” or whatever is appropriate).

We have now defined what we mean by “localisations” and “localisation count” in the main text (in the same discussion covered in minor point #1). We have also generated localisation grids to help display the map of localisations more clearly in Fig panels 2D, 2F, and 3D of the original ms.

4. Pg. 3 line 118: What is meant by “binds the gap well”? It doesn’t appear that there is anything special about the R-seals that make them bind better than other oligonucleotides with the same complementarity pattern.

Since R-seal is essentially a variant library itself, we wanted to state that we need to ensure that R-seal binding always provides measurable binding, which that can then be perturbed by the complementary U-seal to allow the base to be called. In any case, we changed the phrasing to “binds the gap efficiently in order to generate a strong signal”.

5. Pg. 5 line 186: “two DNA strands were hybridized”. This makes it sound like two strands were hybridized to each other, rather than two additional strands being hybridized to the immobilized strand. The language should be clarified.

Indeed. We changed this to “two DNA strands were hybridized efficiently to the surface-immobilized strand in situ”, which should be clear.

6. Pg. 7 line 276. I couldn’t follow what was meant by “...and a 2 A/U nt at the…”.

We changed the text to read “have two A/U nucleotides at the RNA 3'-end”, which should be clear.

7. Y-axes are simply labeled “fluorescence” or “intensity” in a number of figure panels. It would be helpful to specify what the fluorescence is from. For example, in Fig. 4G the y-axis would be labeled “CAP fluorescence” and in 4H it would be labeled “R-seal fluorescence”.

We followed the reviewer’s good suggestion. The y-axis now reads either “seal fluorescence”, “R-seal fluorescence”, or “CAP fluorescence”.

8. It would be helpful to briefly explain in the main text the approach of obtaining a library of one strand of DNA and then extending the other with DNA polymerase in order to obtain a library with only base-paired sequences.

Agreed. We added an abridged version of the variant library protocol in the main text.

9. Fig. 5H-J and S25 show percentages, not fractions (as the captions suggest).

Thanks, this is now corrected.

10. In the plots showing dwell time distributions and fits, the number of molecules analyzed should be provided (in the figure, caption, or a supplementary table).

We have provided the number of molecules N in the captions of 10 SI figs, as requested.

11. Supplement pg. 3 line 81: The concentration of the glycogen stock should be provided.

The concentration was 1 mg/ml, and has been added to the text.

12. Supplement pg. 9 line 349: Why is “all acceptor channels” plural? It would be more clear to identify the channels by their color, since the detected fluorophore is not acting as a FRET acceptor in all cases.

We agree that the phrase is confusing. The plural reflected that the localisations come from 4 sequential seal interrogations. We have now changed “all acceptor channels” to “red-emission channel”.

13. Fig. S3: The x-axis of panel B says “mFOLD server” while the caption says “DINAmelt server”.

The x-axis should say “DINAmelt server”; this is now fixed.

14. Fig. S5A: It would be helpful if the definition of the y-axis of this plot was more specific.

Agreed. We now revised the definitions of both axes to make them more specific, and added them to the plots in the figure panels. X-axis: sum of localisation counts per Gap-G^3 molecule. Y-axis: number of Gap-G^3 molecules.

15. Fig. S6B and a few other places. Many of these plots appear to have a slow component that is not being adequately fitted, even though it is stated that the fits are bi-exponential. What is the significance of this slow component, and is it problematic for it to not be fitted?

Re the S6B fits, we have now fitted the slow component as well. Please also see response to Reviewer 1 comment #3.

Re the origin of the double-exponential decays: the origin is related to the presence of the DNA strands that are flanking the DNA gaps, and provide base stacking at the seal ends. However, the process of hybridization within the gaps is very complex and we are addressing it in separate work that will appear in a future manuscript. For more details and related experiments, please see our response to Reviewer 1 comment #1.

16. Fig. S6-S7, S10-12, S14-S15: It would be helpful to state in the captions what concentration was ultimately selected for the experiments in the main text and why.

In the original ms, the chosen concentrations had all been mentioned in the text of the SI. However, we did follow the reviewer’s suggestion since it does help link the results in the figure to our choice of DNA concentrations.

17. Fig. S10C caption: The plots are left and right, not top and bottom.

Corrected.

18. Fig. S12: Panel D is not labeled in the figure.

Fixed.

19. Fig. S17: The cyan tint of the “no sequence” bars is barely visible. A brighter color or thicker lines would be more clear.

We increased the contrast, recolored the figure and increased the size to make the cyan bars more visible.

20. Fig. S21B: The meaning of the red line is not specified, and it is difficult to see.

Agreed. We defined the red line (NTP addition point), enlarged it, and added a similar pointer to the Rif control. We also enlarge the figure panels.

21. Fig. S21A: The plots and their sequence labels are too small to see clearly. It would be helpful to indicate on the figure what of the categories described in the caption each sequence was assigned to.

(Presumably this is for Fig S23A). Agreed. We enlarged the plots and the sequence labels, increased contrast, and added labels to mark the examples of the behaviours we discuss in the legend.

22. Methods lines 145 and 152: It would be more clear if consistent units were used (mm2 vs µm2).

The unit mm^2 (should have been μm^2) was incorrect, and it has now been fixed.

Reviewer #1: This revised manuscript (NCOMMS-25-94084-T, "Unraveling single-molecule reactions via multiplexed in-situ DNA sequencing") submitted by Hazra et al. has addressed most of the previous comments. However, several issues should be clarified for publication.

1. Regarding the interpretation of double-exponential decay in the lifetime distributions, the authors showed that this behavior arises not only from base-pairing during hybridization, but also from stacking effects at the gap. This is very important mechanistic information. Even if the authors do not add revised data, they should explicitly mention in this manuscript that the heterogeneous behavior originates from the stacking effects at the gap.

We now explicitly mention the role of stacking in the behaviour (in the legend of Fig. 2C).

2. In Fig. S6B, the authors stated that average dwell times represent the expected lifetime of randomly chosen hybridization events. However, in the interpretation of the data in Fig. 2A and 2B, they used t2. The same criterion (average dwell times) should be applied consistently. Alternatively, the authors should clearly explain why different lifetime criteria were used for different datasets.

In the text, we mention that both lifetimes are shortened. The decrease in t_2 is more visible since it is a longer lifetime. We note again that the lifetimes are not used to perform sequencing - our method uses localisation number.

3. The rationale for selecting 100nM/300nM as the optimal condition is not sufficiently clear. In Fig. S7, the dwell time and location graphs must include the corresponding complementary data for clear comparison.

The rationale is explained in the SI (*Methods* section "*Optimisation of sequencing by competitive inhibition*", 2nd paragraph). To aid comparison, we added the points of the complementary experiments from Fig. S6 to panels C and D of Fig. S7; we also updated the legend of Fig. S7 to clarify the rationale.

4. Regarding the R-seal/U-seal optimization at 0.5 uM R-seal, the authors claimed that 1.5 uM U-seal is optimal. However, this conclusion is not clearly supported by the current presentation. To make this conclusion more quantitative, the authors should add and additional graph showing the ratio between complementary and 1-nt mismatch U-seals. The ratio can be easily calculated from the data in Fig. S11 and S12.

(We assume this refers to Fig S12). To aid comparison, we added the points of the complementary experiments from Fig. S11 to panels C and D of Fig. S12. We also updated the legend of Fig. S12 to clarify the rationale.

5. For "G" in the gap in Fig 2F, the dwell times for "G," "A," and "T" are overall high and are not dramatically distinguished from that for "C." In addition, this trend does not appear to be reflected in Fig. 2E and Fig. S1. The authors should explain this.

The absence of large difference in off-rates for G in the gap is likely due to the high stability of 1-nt mismatched seals that proceed to a hybridised seal that stacks both at its 5' and 3' end. Instead, the 1-nt mismatched seals are primarily discriminated by on-rate rate differences, which strongly suggests the presence of partially hybridized states that dissociate before proceeding to a state with full stacking on both 5' and 3' ends. Discrimination based mainly on the on-rate is also seen for other bases in the gap, albeit to a lesser degree. This discussion is relevant to the mechanistic studies we referred to while discussing DNA seals with different levels of stacking; we will expand on this aspect in a future manuscript.

6. In the analysis, 2,000–2,500 molecules were detected in a single FOV, but only 300–600 of them were actually used for sequencing. The authors need to explain why the remaining 75–85% of molecules were excluded from the analysis.

The numbers quoted are estimates from the two protein-DNA systems we studied. Regarding the SPIN-seq assay on transcription initiation (the most demanding of the two), the main factor

reducing throughput is the presence of free DNA in our preparations and the dissociation of the open complex. Since the open complex is immobilised via the biotin on the dsDNA, any free DNA present during the preparation and deposition of open complex will show E_{FRET} similar to the open complex, but obviously the free DNA will not lead to any FRET changes due to transcription; further, some immobilised open complexes will dissociate to free DNA and RNA polymerase during the incubation and washes (since the open complex has a half-life of tens of minutes). We also removed smFRET traces obtained from the edges of the imaged surface due to poor signal-to-noise ratio. Finally, ~10% of all transcriptionally active molecules did not yield a sequence due to various factors (e.g., incomplete gap formation, irreversible sticking to the surface, etc.).

We added a short clarification after our mention of 300-600 usable molecules.

Reviewer #2: The authors have sufficiently addressed our concerns in the revised manuscript. Minor comment: We noted that the figure legend for Figure 2 is missing panel D (likely a typo for C,D).

Indeed. (C) should be (C,D), and it has been changed.

Reviewer #3: The authors have significantly improved the clarity of the manuscript in this revision, as well as adding details important for replication of the study and assessment of the method's robustness. My only remaining questions concern major comment 4 from the original review, which was about assignment of traces in the transcription initiation experiment. While the authors added a lot of useful information in response to this comment, the provided details raise a few further questions/comments.

1. The authors state that "Emission means were initialized near the peaks of the empirical FRET efficiency distribution", but the FRET efficiency distribution is not shown anywhere. A histogram showing the distribution these choices were based on should be shown.

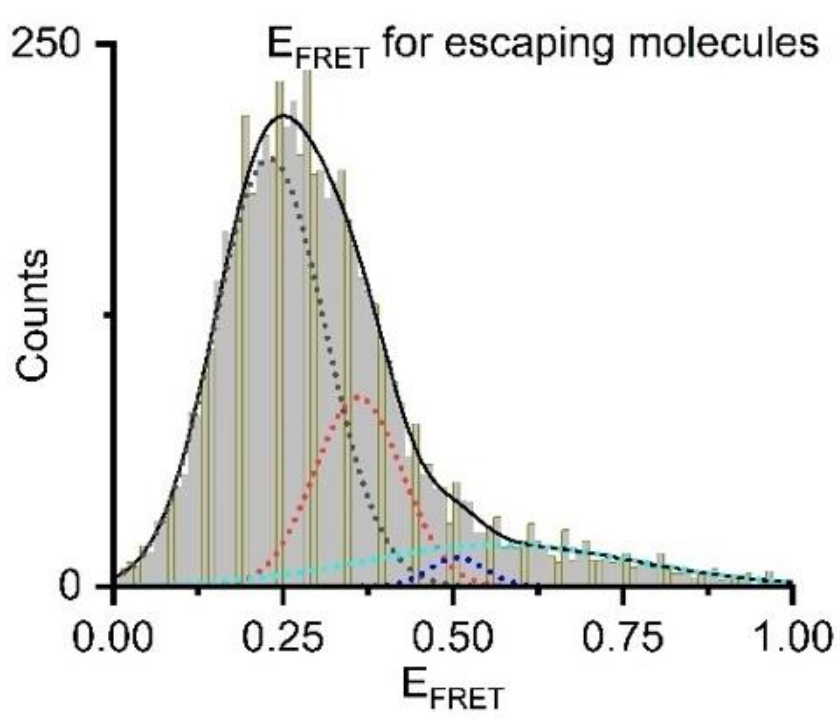

To sample all main FRET states from open complex to an elongation complex (which also includes the main states occupied by cycling or paused molecules), we combined all traces corresponding to escape events (i.e., the ones showing transitions that sampled the E_{FRET}~0.6 state) and plotted the E_{FRET} histogram prior to donor or acceptor bleaching; we then fitted the distribution using a sum of Gaussian distributions. Since the E_{FRET} values corresponding to different initial-transcription stages were known from our previous work (Duchi et al, *Mol Cell* 2016; 63:939. doi: 10.1016/j.molcel.2016.08.011.), we used them as *initial* values for fitting. We also note that the histogram and heatmap of E_{FRET} for individual sequences is shown in Fig. S23.

We added the explanation above in the SI (smFRET data analysis) and the E_{FRET} distribution as **Fig. S22D.**

2. By stating that emission means were "initialized" at empirical values (presumably the EFRET = 0.2, 0.38, 0.5 and 0.6 values mentioned later in the paragraph, which should be clarified), the authors imply that these values were allowed to float during idealization. From the authors' statement that all trajectories were idealized "with shared emission and transition parameters", it is not clear whether only the initial guesses were identical for all traces, or whether the emission means were kept identical across all traces but allowed to float relative to their initialized values (e.g. the algorithm could find that an initial guess of 0.38 is better modeled as 0.41, giving all molecules an idealized value of 0.41 when their traces dwell at FRET efficiencies in that ballpark). These details should be made clear.

In our analysis, emission means were initialized near the major biologically relevant peaks of the E_{FRET} distribution. A single global HMM was trained by fitting all trajectories together, such that emission and transition parameters were shared across all traces. As such, while emission means were allowed to vary during training, they were not fitted independently for each trajectory. Instead, they were optimized globally, resulting in a single set of emission means common to all molecules. For example, an initial value of 0.38 could converge to a nearby value (e.g., ~0.40) if supported by the data. Since emission means are defined globally, individual FRET values falling between idealized state levels (e.g., ~0.40) were assigned to the nearest state based on the emission distributions. To ensure robustness, the fitting procedure was repeated with multiple random initializations (100 restarts), and the solution with the highest likelihood was selected.

We added these clarifications in the SI (smFRET data analysis).

3. What fraction of the traces were ambiguous, and how were they handled? This is particularly important if EFRET values were allowed to float individually for traces, as some traces may have ended up with states at FRET efficiencies that were ambiguous relative to the initial model, such as ~0.45.

As discussed above, emission means were determined using a single global HMM fit to all trajectories. A few trajectories (17 traces, ~0.8% of all transcriptionally active molecules) exhibited paused E_{FRET} values in the 0.45-0.50 range, which did not clearly correspond to the main defined paused states. These traces were classified as "ambiguous". Given their low abundance and lack of clear functional state assignment, these traces were excluded from further analysis; inclusion of these traces did not affect the overall distribution of FRET states or our any of conclusions.

We added this discussion in the SI (smFRET data analysis).

4. The authors state that the state number was "validated for robustness". They should briefly describe what this validation entailed.

The FRET traces in transcription are heterogeneous due to effects of varying sequences. To find the optimal number of states, we fitted the traces with HMM using 2 to 5 states. To find the best fit, we used the metrics of log-likelihood (logL) and change in log-likelihood (ΔL). LogL measures how well a given model explains the observed FRET data, and is computed as the logarithm of the probability of observing the data under the fitted model; higher log-likelihood indicates a better fit to the data. On the other hand, ΔL quantifies the improvement in model fit when increasing the number of states: a large and positive ΔL implies that the addition of a state significantly improves the model, a ΔL ~ 0 means little or no improvement, and a negative ΔL indicates overfitting. Model complexity was also evaluated using the Akaike Information Criterion (AIC) and Bayesian Information Criterion (BIC), which quantify the trade-off between goodness-of-fit and model complexity. Both criteria are defined as a function of the log-likelihood and include a penalty term that increases with the number of model parameters (i.e., the number of states).

$$AIC = -2logL + 2p$$

$$BIC = -2logL + p\log(N)$$

$$p{:}\ number\ of\ independent\ parameters, N{:}\ number\ of\ observations$$

While increasing the number of states generally improves the likelihood, AIC and BIC penalize additional model complexity. Model selection is based on relative comparisons, with the lowest AIC or BIC indicating the optimal trade-off between fit and complexity.Model selection analysis revealed a substantial increase in log-likelihood when increasing from two to three states (ΔL ≈ +21.9), indicating that a three-state model captures the dominant structure of the data. Increase to four states showed some improvement (ΔL ≈ +9.5), suggesting the presence of additional, less-populated states. The improvement from 4 to 5 states was smaller (ΔL ≈ +7.2), indicating diminishing returns. Consistent with this, the AIC reached a minimum at four states, while the BIC (which imposes a stronger penalty for model complexity) favoured a three-state model. Overall,

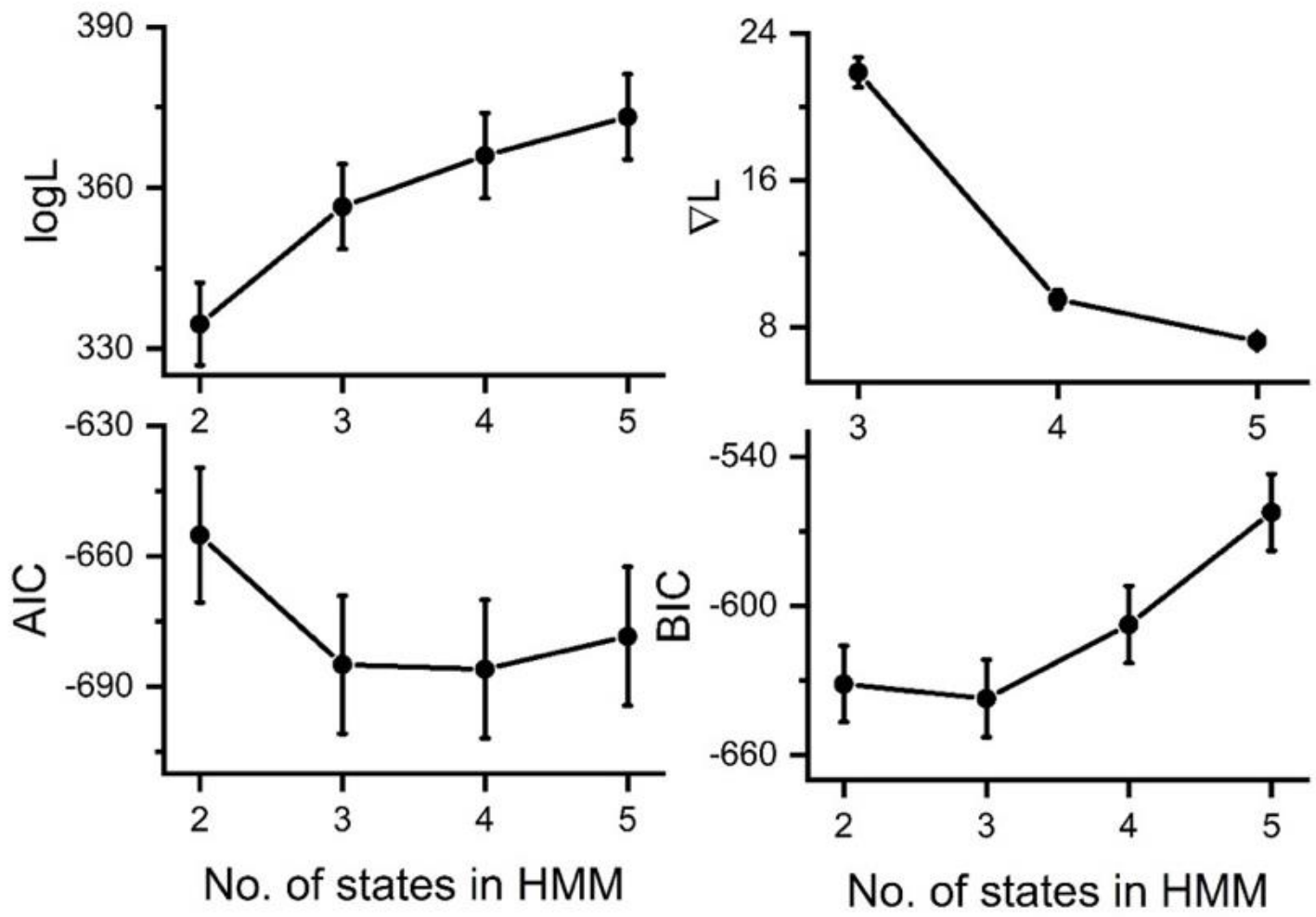

these results indicate that the three-state system is the dominant behaviour, while a four-state model captures the presence of a less abundant FRET state (the ~0.51 state).

We also tested the method by repeating the analysis on randomly subsampled datasets of four independent experiments and found that the identified FRET states and their approximate positions were preserved; we observed similar trends in logL, ΔL, AIC and BIC values.

Together, these analyses demonstrate that the selected state number provides a robust and reproducible description of the data.

We included a version of the above in the SI (smFRET data analysis), and the plots as **Fig. S22E**.

5. The second paragraph describing HMM (“Following HMM idealization…”) states that Class III molecules showed transitions from 0.2 to 0.4, but 0.4 is not listed as one of the FRET efficiencies used to initialize the model. Is this a typo, or does it refer to the 0.38 state? If emission means were allowed to float for molecules individually during HMM, can an EFRET ~0.4 be reliably distinguished from EFRET ~ 0.38?

Yes, the 0.4 state is indeed the ~0.38 pausing state. The ~0.4 was used as an approximation of the state, but it was used inconsistently, and causes confusion – so we now refer everywhere to the ~0.38 state.

Point-by-point response to Reviewers

Reviewer #1 (Remarks to the Author): Dear editor - all issues that I raised are addressed and figured out. This manuscript is ready to publish in Nature Communications.

Response: we thank the reviewer for their time and helpful comments.

Reviewer #3 (Remarks to the Author): The revised supplementary information provides methodological clarity regarding HMM initialization, global fitting strategy, ambiguous trace handling, and state-number validation. The transcription initiation analysis has been substantially improved by these additions. I offer just two aesthetic suggestions for the new SI text: (1) Cite the previous work in the manner used elsewhere in the supplement rather than including its full citation in the text, and (2) subscript "FRET" in E_{FRET} as done in the main text.

Response: We thank the reviewer for their time and helpful comments. We now cite the past work as for the Main text references. We have also revised the SI to use the E_{FRET} term.

Reviewer #4 (Remarks to the Author): I co-reviewed this manuscript with one of the reviewers who provided the listed reports. This is part of the Nature Communications initiative to facilitate training in peer review and to provide appropriate recognition for Early Career Researchers who co-review manuscripts.

Reviewer #4 (Remarks on code availability): I did not independently review or execute the supporting code as part of this assessment.

Response: we thank the reviewer for their time and helpful comments.
